# Nuclear morphometrics and chromatin condensation patterns as disease biomarkers using a mobile microscope

**DOI:** 10.1371/journal.pone.0218757

**Published:** 2019-07-17

**Authors:** Karthik Damodaran, Michele Crestani, Doorgesh Sharma Jokhun, G. V. Shivashankar

**Affiliations:** 1 Mechanobiology Institute and Department of Biological Sciences, National University of Singapore, Singapore, Singapore; 2 Institute of Molecular Oncology, Italian Foundation for Cancer Research, Milan, Italy; Pennsylvania State Hershey College of Medicine, UNITED STATES

## Abstract

Current cancer diagnosis involves the use of nuclear morphology and chromatin condensation signatures for accurate advanced stage classification. While such diagnostic approaches rely on high resolution imaging of the cell nucleus using expensive microscopy systems, developing portable mobile microscopes to visualize nuclear and chromatin condensation patterns is desirable at clinical settings with limited infrastructure. In this study, we develop a portable fluorescent mobile microscope capable of acquiring high resolution images of the nucleus and chromatin. Using this we extracted nuclear morphometric and chromatin texture based features and were able to discriminate between normal and cancer cells with similar accuracy as wide-field fluorescence microscopy. We were also able to detect subtle changes in nuclear and chromatin features in cells subjected to compressive forces, cytoskeletal perturbations and cytokine stimulation, thereby highlighting the sensitivity of the portable microscope. Taken together, we present a versatile platform to exploit nuclear morphometrics and chromatin condensation features as physical biomarkers for point-of-care diagnostic solutions.

## Introduction

Recent developments in mobile camera have paved the way for the use of mobile microscope as a useful low cost tool for cellular and tissue imaging [1, 2]. A number of studies have demonstrated its application in visualizing multi-cellular systems such as microbes and tissues [1, 3, 4]. In addition, several studies have also shown the ability of mobile microscopes to capture static features of the cell, nucleus as well as single DNA molecules [2, 5–7]. Further it has also been used to measure the dynamic features of the cell such as its motility [8]. These methods have opened new avenues for mobile microscopes to be used as tools for clinical diagnostics to assess different types of human samples. Some examples include parasite detection in whole blood samples [9–12], detection of soil-transmitted worms and cysts in stool samples [13–15], detection of pathogens in urine samples [16], diagnosis for tuberculosis using sputum samples [9, 17, 18], measuring sperm concentration and motility [19] as well as cancer cell detection using brush biopsy for oral cancer [2]. Several efforts have also been made to exploit the advancement in digital connectivity for the purpose of tele-consultation [20–22]. These technologies are making mobile microscopes versatile and low cost platforms for telemedicine and point-of-care solutions.

While the aforementioned works have made breakthroughs in image acquisition of tissues, single cells and even DNA, acquiring high resolution images of the nucleus and chromatin using a low cost mobile microscope is not well developed. Nuclear morphological features and chromatin condensation patterns are extensively used as biomarkers for various pathological conditions [23–34]. Conventionally, pathologists would manually scrutinize patients’ samples for defects in nuclear shape, size and envelope as well as abnormalities in chromatin condensation and texture for diagnostic purposes. While advances in digital image acquisition and processing helps pathologists with detailed nuclear and chromatin analyses, conventional microscopy systems are bulky, complicated and expensive, limiting their use as point-of-care solutions in remote regions where high-end infrastructure is lacking. As such, a push for mobile microscopy systems capable of acquiring high resolution nuclear and chromatin features is critical for opening up the existing bottleneck in efficient large-scale tests for cancer and aging related diseases.

In this study, we developed a small portable device which can be attached to a mobile phone and function as a mobile fluorescent microscope capable of high resolution nuclear and chromatin imaging for disease diagnostics. We use a conventional wide-field microscope to establish a group of nuclear morphological and chromatin textural signatures in normal and cancer cells as a benchmark to assess the detection capabilities of our mobile microscope. We imaged the same cell-types on the mobile microscope and showed that we were able to extract the nuclear morphological as well as chromatin textural features for discriminating between the different cell types. Importantly, our mobile microscope was able to distinguish subtle changes in chromatin texture patterns upon extracellular perturbations in both normal and cancer cells. Collectively, our results highlight the potential of using the mobile microscopy system as a convenient tool for large-scale diagnostic programs in remote regions.

## Materials and methods

### Design of the smartphone microscope

#### Illumination scheme

The components (S4 Fig) of the smartphone microscope were designed by Autodesk Tinkercad and subsequently printed with a 3D printer (Ultimaker 3, Ultimaker). The assembled microscope consists of a diffused axial illumination scheme, in which the light emitted by an UV star LED (LZ4-04UV00, LED Engin) is filtered by a low-bandwidth DAPI emission filter (#84–094, Edmund Optics). The light beam is perpendicularly delivered onto the sample using a Precision Aspheric Lens (#69–852, Edmund Optics, f = 7.5 mm) as a condenser lens. The UV LED, the DAPI emission filter and the condenser lens are accommodated together with a heatsink (ATS1302-ND, Digi-Key) bound to the UV LED in a small box. The UV LED is powered up with an 18V 40W AC/DC converter (#1145-1001-ND, Digi-Key) connected to the mains electricity in order to provide a stable illumination of the sample. A 100 Ω resistance is used to limit the current flowing through the UV LED.

#### XYZ moving stage

The removable sample tray is inserted in a translational stage for XY horizontal movement, giving the possibility to explore a ~ 20 x 20 mm surface area of the sample. The XY translational stage (S4D and S4E Fig) is made of 2 dovetail rail carriers where screws are permanently inserted in small cases. Screws are driving the movement of the carrier where the sample tray is inserted. In particular, the Y translational stage is directly connected to the sample tray, while the X translational stage is attached to the Y stage (S4D and S4E Fig). The small box and the XY translational stage are both mounted on the same custom-designed Z translational stage for focus adjustment (S4F Fig), where a screw driving for the vertical movement is inserted in a guide below the Z stage. The vertical movement for focus adjustment is transmitted via 3 gears coupled with the Z screw (S4A–S4C Fig), which can be moved from the external side of the microscope. The final XYZ translational stage is inserted in 2 mechanical guides present on 2 inner sides of the box to prevent it from tilting (S4F Fig).

#### Lens system

The DAPI fluorescent signal obtained is collected by 2 lenses, a 60X oil immersion microscope objective and a Nurugo Micro (Nurugo) coaxially placed in front of the smartphone camera. A DAPI emission filter (#84–095, Edmund Optics) is interposed between the 2 collecting lenses for background rejection. The 2 lenses and the DAPI emission filter are accommodated inside a 3D printed case. The final mobile microscope consists thus of a small box of 10.5 x 15.5 x 16 cm in dimensions.

#### Cell culture and sample preparation for the mobile microscope

All experiments were performed in accordance with relevant guidelines and regulations at National University of Singapore (NUS). NIH/3T3 mouse fibroblast (CRL-2522, passage 30), MCF-7 breast cancer cells (HTB22) and BJ human fibroblast (CRL-2522) were obtained from ATCC, grown in high-glucose DMEM media (Gibco, Life Technologies) supplemented with 10% v.v. FBS (Gibco, Thermo Fisher Scientific) and 1% penicillin−streptomycin (Gibco, Thermo Fisher Scientific) and maintained at 37°C and 5% CO_2_. HTERT-HME1 human epithelial cells were obtained from ATCC (CRL-4010) and grown in MEBM medium supplemented with Clonetics MEGM bulletkit (Lonza, CC-3150). Cells were isolated by trypsinization (3–5 min) and plated in the custom-made PDMS microwell or on a 20 x 20 mm coverslip (Cat No. 01 010 40, Trade 21).

Microwell were fabricated by plasma bonding a poly(dimethylsiloxane) (PDMS,Sylgard 184 Silicone elastomer kit, Dow Corning, Midland, MI, USA) layer of few mm in thickness onto a 20 x 20 mm coverslip (Trade 21, 01 010 40). Prior to bonding, the ~ 10 x 10 mm PDMS layer was punched in the middle with a 5 mm biopsy puncher to create the well. PDMS was obtained by mixing silicone elastomer and curing agent at a 10:1 v.v. ratio, followed by degassing and polymerization at 80°C for 2 hours. Once PDMS was polymerized, it was cut in ~ 10 x 10 mm squares and punched to create the hole, taped and sterilized in autoclave for 20 min at 120°C before proceeding with plasma bonding. Cells were fixed with 4% parafoldehyde (PFA) (Sigma, 252549–500 ml) for 10 minutes.

### Compressive force experiment

For compressive force experiments, around 10000 cells were plated on a 20 x 20 mm coverslip and allowed to attach such that the confluency is around 90%. The experiment was performed as described previously with slight modifications [35]. A pluronic acid-treated 18x18 mm coverslip was placed on the cells. The compressive force was applied by placing parafilm-wrapped metallic nuts (mass = 23g) on the coverslip. This corresponds to each cell experiencing a force in the order of micro-newtons (calculation shown below). For recovery, the metallic nut was removed and the cells were allowed to recover for one hour before being fixed for immunofluorescence staining.

The force was computed as follows:

Mass = 23g (23 x 10^-3^kg); force applied (weight) = mass x acceleration due to gravity = 23 x 10^−3^ x 10 = 230mN;

$$\frac{Force}{cell}=\frac{Compressive\;force-buoyant\;force\;due\;to\;1ml\;media}{number\;of\;cells}$$

Buoyant force = weight of volume of media displaced; volume of media displaced by a single nut = 1ml (1g); mass equivalent to 1/20^th^ of the nut that is submerged in the media during the experiment = 1.1g; number of cells = 10,000.

$$\frac{Force}{cell}=\frac{(23-1.1)\;X\;10^{‐3}\;X\;10}{10000}=21.9\;\mu N$$

For compressive force experiments, fixation was performed by treating the cells with 4% PFA for 20 minutes in the presence of the load. Thereafter, the load as well as the coverslip were removed. The samples were subsequently covered by gently placing a new 18x18 mm coverslip (Trade 21, 01 070 32) on top. Nail polish was used to permanently seal the edges of the 2 coverslips which also enables moisture retention by the samples.

Coverslips and the inner microwell surface were coated with a PBS solution of Fibronectin (Sigma F1141-2MG) 1:150 dilution for 45 minutes at 37°C prior to cell seeding. For nucleus staining a DAPI solution (ThermoFisher Scientific R37606) in PBS was employed as recommended by the supplier.

For experiments involving tumor necrosis factor α (TNF-α) and Cytochalasin-D (Cyto-D) the stimulation was applied for 30 min with a working concentration of 0.4 nM and 1 nM respectively prior to cell fixation.

1 μm (T7282, Life Technologies) and 10 μm (F8829, Life Technologies) DAPI fluorescent beads were sonicated for 30 min and injected inside a 3.5 mm microwell. In particular, 15 μl of bead solution 1:6 dilution in distilled water were injected in the microwell and allowed to evaporate. Upon evaporation, 15 μl of distilled water were injected as mounting medium prior to imaging.

### Image acquisition workflow for Mobile microscope

The cell sample of interest was loaded on the sample tray and inserted in the translational stage of the mobile microscope. A drop of immersion oil standardized for 23°C usage was added on the coverslip before imaging. Every fluorescence image utilized for the analysis was recorded in a lossless digital negative (DNG) format with an integration time of 1.3 s. ISO sensitivity was left auto when PDMS microwell was employed, while was set to 400 or 800 when the sealed coverslip was employed, due to the different optical path. Smartphone autofocus was set to infinity. Multiple frames (N ≥ 4) of each region of interest were captured. Image averaging (N ≥ 4) was performed with Adobe Photoshop CC 2018 in lossless DNG format after monochrome conversion, white balance adjustment (with Photoshop plugin Camera Raw 10.3) and alignment of the nuclear edges. DNG images are indeed displayed with no compression and demosaicking (i.e. color reconstruction) steps (Abobe Photoshop: Digital Negative (DNG). http://helpx.adobe.com/photoshop/digital-negative.html) [7]. The averaged image was converted to 8-bit TIFF format for background subtraction using a rolling ball algorithm and median filtering (radius = 1 pixel) for further noise reduction utilizing a custom macro in ImageJ/Fiji.

### Imaging using conventional wide-field microscope

Cells were seeded on glass bottom dishes (Ibidi 81158) and allowed to attach overnight. After fixing and staining the nuclei, images were obtained using an Applied Precision DeltaVision Core microscope. Wide field images were obtained using 60X objective (air, NA 0.7) with a pixel size of 0.2150 μm. These 512 × 512 12-bit images were deconvolved (enhanced ratio, 10 cycles) and saved in tiff format.

### Image analysis

Unless otherwise specified, custom-written codes in MATLAB (The MathWorks, Natick, MA) were used for data analysis and graphs were plotted using Origin (OriginLab, Northampton, MA) and MATLAB (The MathWorks, Natick, MA).

### Autocorrelation length-scale (spatial correlation)

In order to quantitatively characterize the DNA intensity pattern manifested in each nucleus, single nuclei were first segmented from DAPI images and their autocorrelation length-scales were determined using a modified version of the method described previously [36]. As illustrated in S1 Fig, the largest possible circle was cropped from the nucleus and its intensity was scaled from zero to one. The mean intensity was then subtracted from each pixel such that intensities in the image would vary with a mean of zero. This zero-mean normalized circular image was finally used for the following analysis.

2D image correlation was used to calculate the correlation coefficients between the image and a copy of itself, shifted by n pixels in every possible direction (-diameter of the circle < n < diameter of the circle). As the copy is shifted, the correlation coefficient decreases and the rate of decrease is dependent on the sizes of the structures inside the nucleus. The distance at which the correlation coefficient becomes zero corresponds to the number of shifts whereby, on average, the bright structures inside the nucleus are matched with the dark structures. Since we had the correlation coefficients for shifts in every direction, the mean was taken and plotted as a function of distance (shifts). The distance at which this mean curve crossed the x-axis is a measure of the average length-scale of the pattern inside the nucleus.

### Principle Component Analysis (PCA)

Data from all the cells being analysed were first concatenated. The combined dataset vector for each parameter was then standardized by subtracting its mean and dividing by its standard deviation. As a result, each parameter would be a vector of values from all the cells with a mean of 0 and a standard deviation of 1.

The in-built PCA function in MATLAB was then applied as follows:

[coeff,score,latent] = pca(standardized_CombinedData)

The output matrix ‘coeff’ contains the loading coefficients of each parameter on each principle component. The output matrix ‘score’ contains the projection value of each data point in the principle component space (feature space). And the output matrix ‘latent’ contains the principle component variances.

Since the number of nuclei from each sample was known, while plotting the data in the feature space, data from different samples were given different colours. This enabled us to visualize the space occupied by each sample in the feature space.

## Results and discussions

### Nuclear morphometric and chromatin textural features discriminate different cell types imaged on a conventional wide-field microscope

**Fig 1:**
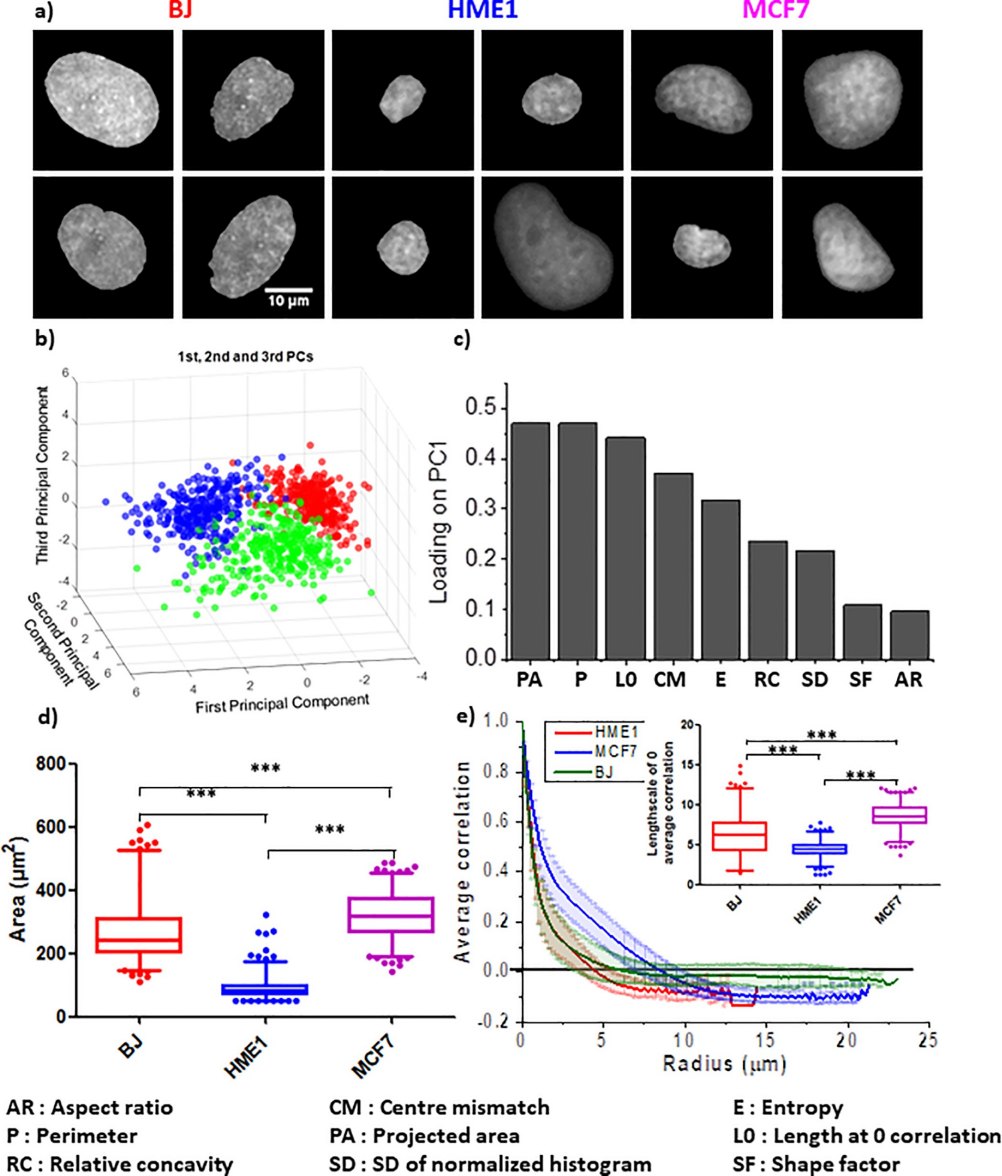
Establishing nuclear shape and chromatin features as a measure for diagnosis. **a**) Representative wide-field images of the nuclei acquired using Deltavision microscope. Scale bar: 10**μ**m. b) PCA plot showing the segregation of BJ, HME1 and MCF7 nuclei in a feature space based on a linear combination of their morphometric and textural features. c) The loading coefficient of each parameter used to obtain the first principle component of the PCA plot in (b). d) Whisker box plots (2.5% to 97.5%) showing the distribution of nuclear projected area for BJ, HME1 and MCF7. e) Spatial autocorrelation of DAPI intensity as a function of length-scale for BJ, HME1 and MCF7 nuclei (see methods for more details). The lines show the average of all the nuclei of each cell line and the bars represent the standard deviation. The whisker box plots (2.5% to 97.5%) in the insert shows the distribution of length-scales at which the correlation coefficient drops to zero. N: BJ = 300; HME1 = 389; MCF7 = 321.

DAPI stained nuclear images were obtained from three different cell lines for this study i.e. normal human foreskin fibroblast (BJ), immortalized normal human mammary epithelial cells (HME1) and metastatic human breast cancer cell line (MCF7). The representative images are shown in Fig 1A. Each nucleus from an image was cropped using custom program (as described in methods) and saved as an individual 12 bit image for further analysis. Nuclear morphometric and chromatin textural features are the two main types of information which can be obtained from DAPI intensity images [32, 37]. To explore the feasibility of automatically distinguishing between the above-mentioned three cell lines, just from DAPI intensity images, the following nine parameters were extracted from single nuclei imaged under a widefield microscope (described in Table 1): (1) Projected Area, (2) Aspect Ratio, (3) Perimeter, (4) Shape Factor, (5) Relative Concavity, (6) Centre-Centroid Mismatch, (7) Entropy, (8) SD of Normalized Intensities and (9) Autocorrelation Length-scale. While single parameters can sometimes distinguish a cell type from another one, it is not likely to be successful when there are more than two cell types involved. At the same time, it is challenging to simultaneously visualize more than two or three parameters on a single graph. A dimension reduction approach, Principle Component Analysis (PCA), was therefore adopted and each nucleus was projected in the resulting feature space (Fig 1B). Interestingly, we found that nuclei from each of the three cell lines occupied distinct regions in the feature space (Fig 1B). Around 83% of the variance in the data was explained by the first three principle components (PC1, 2 and 3) (S2 Fig). As shown in Fig 1C), the morphometric parameter with the highest loading coefficient on PC1 was the Projected Area while the textural parameter with the highest loading coefficient was the Autocorrelation Length-scale. Graphs for these individual parameter are shown in Fig 1D and 1E). It’s worth noting that while HME1 nuclei have smaller projected areas compared to BJ or MCF7 nuclei, BJ and MCF7 nuclei cannot be well separated from one another based on their projected areas only (Fig 1D). The same is true for the length-scale of internal structures. The average decorrelation curve for each cell type intersects the x-axis at different points (Fig 1E). However, when individual nuclei are considered, though HME1 and MCF7 separates well, there is significant overlap between each of them and BJ (insert in Fig 1E). Decorrelation curves for individual nuclei of each cell type have been plotted in S3 Fig and the loading coefficients of PC2 and 3 are shown in S2 Fig. This highlights the feasibility of using a combination of morphometric and textural features from DAPI images to efficiently segregate nuclei of different cell types. Taken together, this suggests that using a combination of nuclear morphometric and chromatin textural features we can distinguish between different cell types such as fibroblasts and epithelial cells as well as normal and cancerous cells. Although high quality widefield images are very useful, such sophisticated microscopy systems are not always available in clinical settings, especially in under-developed and remote rural areas. It is therefore highly desirable to develop cheaper systems with the capability of discriminating normal cells from cancerous ones, based on nuclear and chromatin features. It would also be preferable for such a device to have a small and portable design.

**Table 1:** Description of parameters.

Parameter	Description	Type
Projected Area	Projected area of the nucleus	Nuclear morphometric
Aspect Ratio	Length of the major axis/length of the minor axis of the nucleus	Nuclear morphometric
Perimeter	Perimeter of the nucleus	Nuclear morphometric
Shape Factor	Circularity given by $=\frac{{Perimeter}^{2}}{4\pi \;x\;Area}$	Nuclear morphometric
Relative Concavity	$=\frac{Convex\;area-area}{Convex\;area}$	Nuclear morphometric
Centre-Centroid Mismatch	Distance separating the centroid of the nucleus and the centre of mass of the chromatin intensities	Nuclear morphometric & Chromatin Textural
Entropy	Statistical measure of randomness in the distribution of intensities given by -sum(p.*log2(p)), where p contains the normalized histogram counts. The in-built MATLAB function J = entropy(I) was used for the calculation.	Chromatin Textural
SD of Normalized Intensities	Standard deviation of the distribution of normalized intensities	Chromatin Textural
Autocorrelation Length-scale	Length-scale of internal chromatin structures (see methods for more details)	Chromatin Textural

### Implementation of fluorescent imaging of nuclei on a mobile microscope

**Fig 2:**
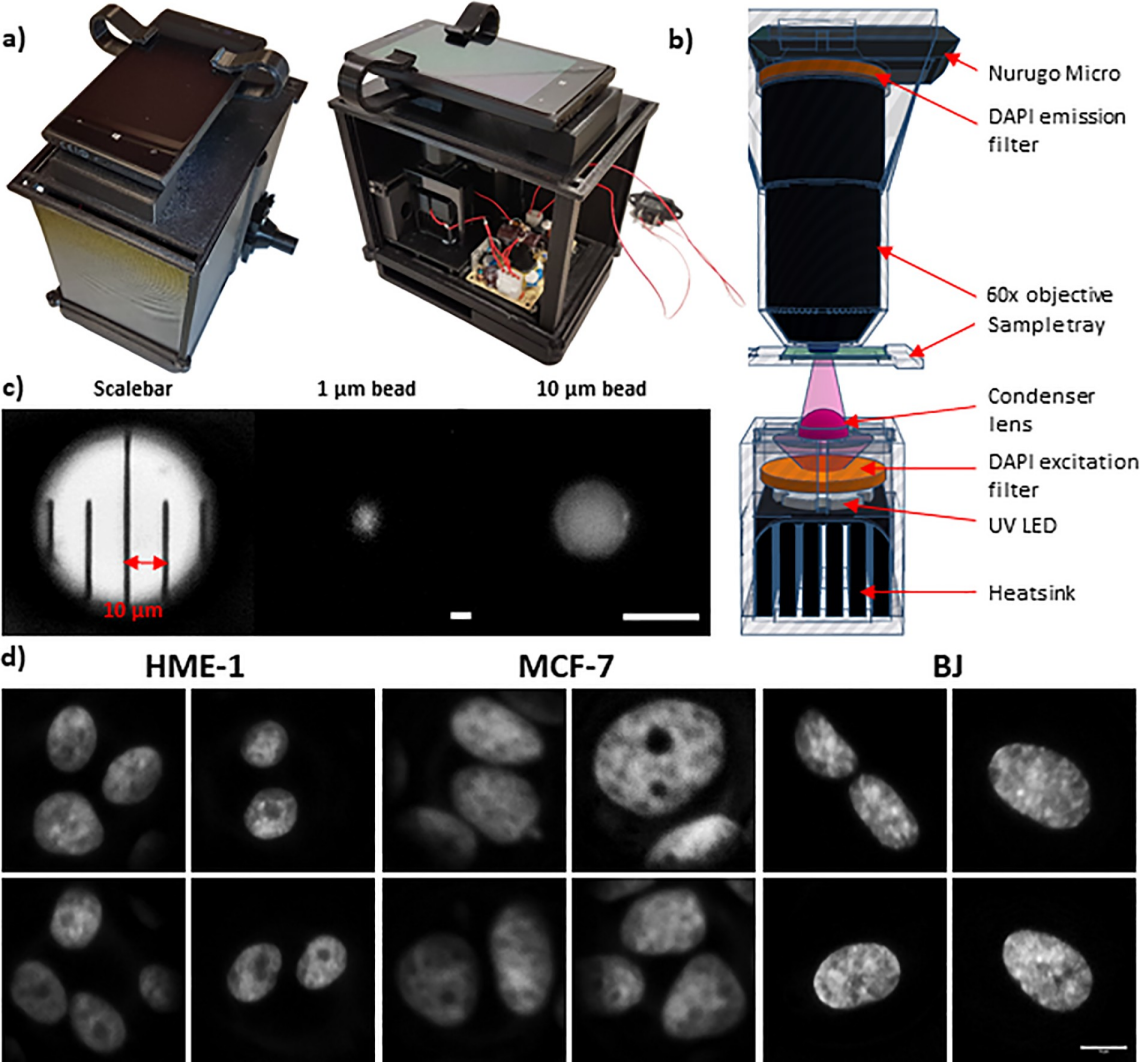
Implementation on mobile microscope. a) Top and side view of the mobile microscope. b) TinkerCAD schematic illustration for the optical path. Colour code of the components: grey transparent for 3D printed parts, black for collecting lenses and heatsink, grey for UV LED, orange for DAPI filter set, purple for UV light beam and condenser lens, green for the sample and blue for the drop of immersion oil. c) Calibration of the mobile fluorescent microscope. Graduated scale bar with 10 **μ**m pitch. Picture of a 1 **μ**m DAPI fluorescent bead. Picture of a 10 **μ**m DAPI fluorescent bead. Pixel size = 0.043 **μ**m. Scale bar: 10**μ**m. d) Representative images of the nuclei acquired using mobile microscope. Scale bar: 10**μ**m.

In order to address the above mentioned problem, we developed a simple and portable mobile phone microscope that is capable of acquiring wide-field fluorescent images. The actual image of the device is shown in Fig 2A. The optical path and the schematic diagram are shown in Fig 2B (described in detail in the methods section). Incorporation of a fluorescent light source and the appropriate filters enabled us to obtain clear fluorescent images of nuclei. The microscope was calibrated using a ruler with markings 0.01mm (10μm) apart, 1μm beads as well as 10μm beads (Fig 2C) and the pixel size was found to be 0.04μm. Using this device, we were able to capture chromatin textural patterns at submicron resolution from intact nuclei, a rich mine of useful features for diagnostic purposes. The representative DAPI stained nuclei images obtained using the mobile microscope after processing are shown in Fig 2D. We next performed similar analysis on images acquired using the mobile microscope.

### Nuclear morphometric and chromatin textural features discriminate different cell types imaged on the mobile microscope

**Fig 3:**
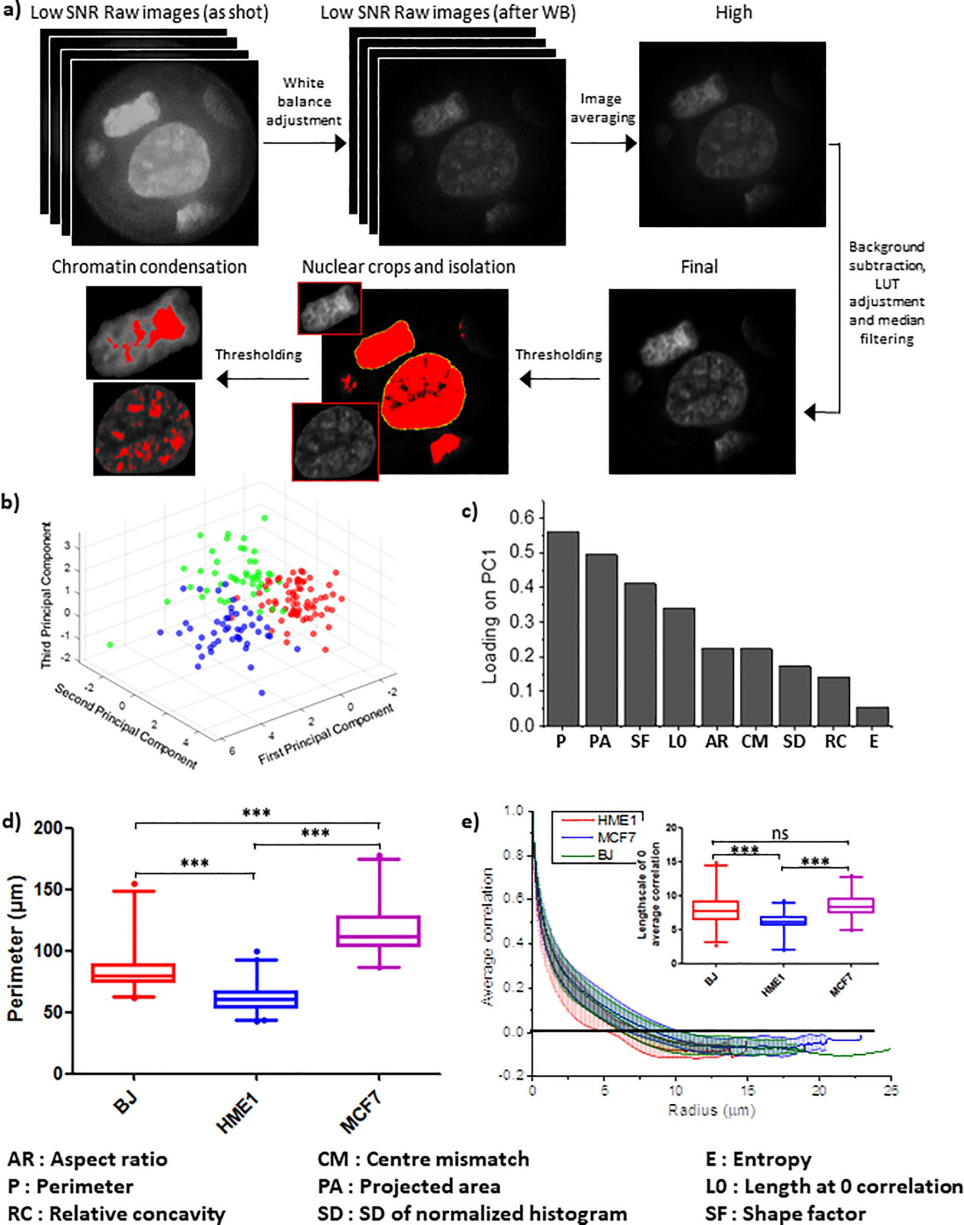
Nucleus segmentation and analysis of chromatin features in mobile microscope. a) Image processing workflow for the raw and low signal to noise ratio (SNR) images acquired using the mobile microscope. Series of mobile phone images (N ≥ 4) of representative MCF7 nuclei were captured in DNG format and converted to black and white mode. White balance was performed using Adobe Photoshop CC 2018 plugin Camera Raw 10.3. Averaged image was formed upon alignment of the nuclear edges. Final image was generated upon background subtraction, LUT adjustment and median filtering for final noise reduction. Thresholding was then performed for single nucleus isolation and chromatin texture identification. b) PCA plot showing the segregation of BJ, HME1 and MCF7 nuclei in a feature space based on a linear combination of their morphometric and textural features. c) The loading coefficient of each parameter used to obtain the first principle component of the PCA plot in (b). d) Whisker box plots (2.5% to 97.5%) showing the distribution of nuclear perimeter for BJ, HME1 and MCF7. e) Spatial autocorrelation of DAPI intensity as a function of length-scale for BJ, HME1 and MCF7 nuclei (see methods for more details). The lines show the average of all the nuclei of each cell line and the bars represent the standard deviation. The whisker box plots (2.5% to 97.5%) in the insert shows the distribution of length-scales at which the correlation coefficient drops to zero. N: BJ = 54; HME1 = 83; MCF7 = 53.

Raw low signal to noise ratio (SNR) images were first obtained using the mobile microscope. This was further processed to obtain the final tiff image which was then further segmented to generate individual nucleus crops and thresholded to analyse chromatin texture (Fig 3A and detailed in methods section). After processing and segmenting individual nuclei from DAPI images taken by the mobile microscope, they were passed through the same analysis pipeline as the images taken by the conventional wide-field microscope. Consistent with the results from images taken on the conventional microscope, nuclei imaged on the mobile microscope also occupied distinct regions in the feature space (PCA plot) according to their cell type (Fig 3B). Around 75% of the variance in the data was explained by the first three principle components (PC1, 2 and 3) (S5 Fig). Although this was marginally less than that observed with data from the conventional microscope (around 83%), there was a clear separation of the different cell types within PC1, 2 and 3 (Fig 3B). The loading of the different parameters on PC1 was also slightly different with images from the mobile microscope. The top morphometric parameter was Perimeter and the top textural parameter was the Autocorrelation length-scale (Fig 3C). Graphs for these individual parameters are shown in Fig 3D and 3E). Decorrelation curves for individual nuclei of each cell type have been plotted in S6 Fig and the loading coefficients of PC2 and 3 are shown in S5 Fig. Interestingly, while differences in the loading coefficients reflect differences in illumination, optical path, camera sensitivity and post-acquisition image processing between the conventional and the mobile microscope, each cell type still occupies a distinct region in the PCA-based feature space (Fig 3B). This highlights the potential and practicality of using the simple low-cost mobile microscope as a diagnostic tool to identify abnormal nuclei based on nuclear morphometric as well as chromatin textural features. Due to the possibility of different cell types having characteristically different nuclear features, it might be relatively easier to segregate nuclei from different cell types. It is more challenging to discriminate between nuclei of a certain cell type and nuclei of the same cell type from an altered (abnormal, potentially diseased) microenvironment. This suggests a need for the mobile microscope device to be sensitive enough to pick up subtle changes in the nuclear and chromatin features within the same type of cell in order to distinguish cells that are exposed to external stimulus from the control cells.

### Sensitivity of the mobile system assessed by extracellular cues-induced subtle nuclear changes

To assess whether the mobile system is sensitive enough for the aforementioned discrimination capabilities, we selected a normal cell line (BJ), stimulated it with different types of extracellular signals and checked whether the mobile system was able to differentiate between the treated cells and the non-treated ones. Three different treatments were chosen; the actin depolymerising pharmacological drug, Cytochalasin D, the soluble cytokine, TNF-α, and a mechanical stress applied in the form of a compressive load. These treatments were chosen as they roughly imitate what cells experience under physiological conditions. The sensitivity assessment was also performed on a cancer cell line (MCF7).

**Fig 4:**
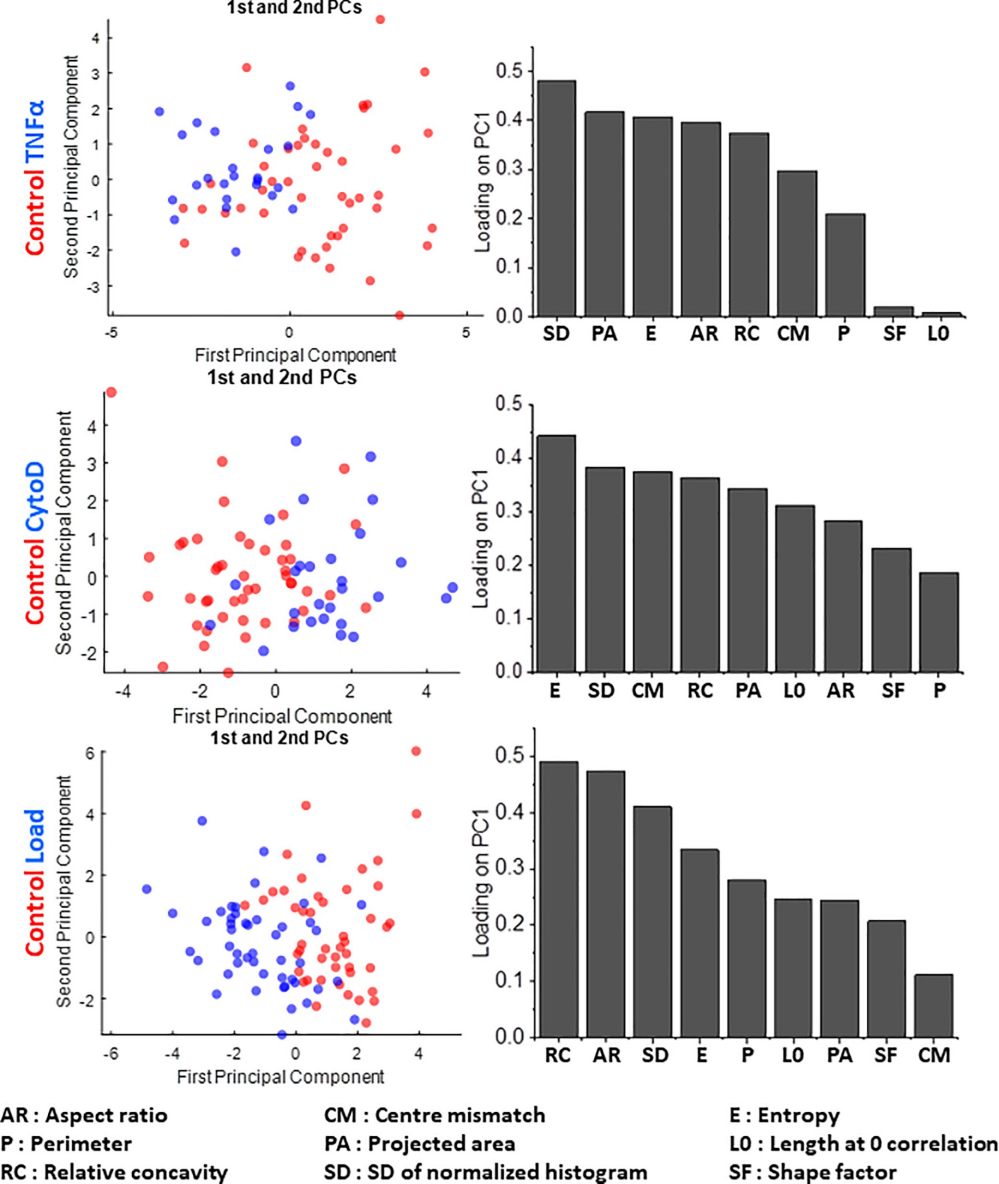
Sensitivity in detecting changes in nuclear and chromatin features upon perturbations in a normal cell line (BJ). PCA plots (left side) showing the segregation of BJ control cells from those subjected to TNFα (top row), CytoD (middle row) and compressive load (bottom row), together with the respective loading coefficient of each parameter (right side) used to obtain the first principle component. N: BJ control = 54; BJ TNFα = 36; BJ CytoD = 40; BJ Load = 54.

**Fig 5:**
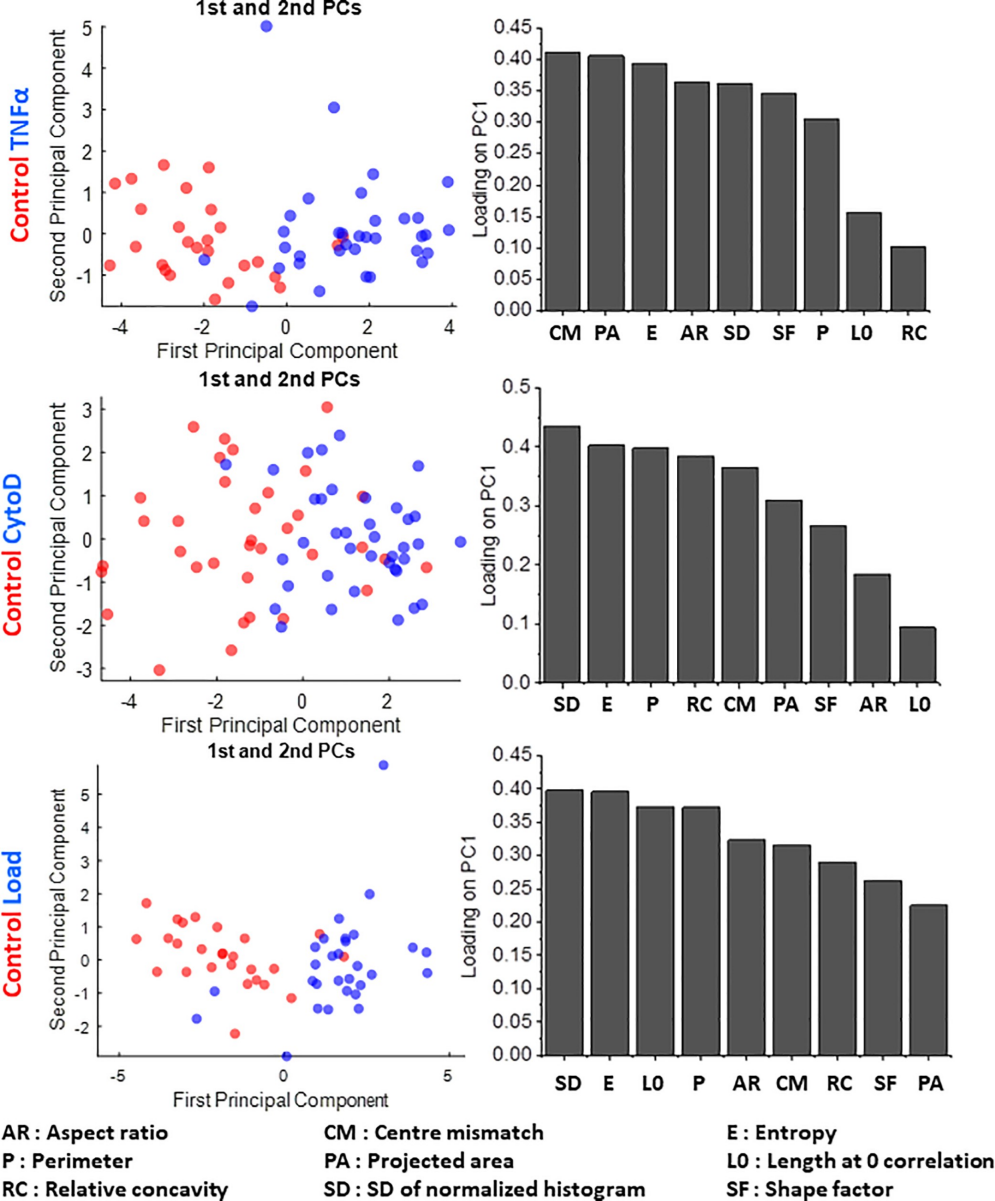
Sensitivity in detecting changes in nuclear and chromatin features upon perturbations in a cancer cell line (MCF7). PCA plots (left side) showing the segregation of MCF7 control cells from those subjected to TNFα (top row), CytoD (middle row) and compressive load (bottom row) respectively, together with the respective loading coefficient of each parameter (right side) used to obtain the first principle component. N: MCF7 control = 52; MCF7 TNFα = 45; MCF7 CytoD = 55; MCF7 Load = 48.

We found that the mobile system was able to discriminate between the Cytochalasin D-treated, TNF-α treated and compressively loaded cell from their untreated control both with BJ and MCF7 cells (Figs 4 and 5). We further observed that the loading coefficients for each parameter on PC1 (which effectively segregated the nuclei) were different in each case. With BJ cells, the top two parameters were SD of Normalized Intensities and Projected Area for TNF-α treatment, while they were Entropy and SD of Normalized Intensities for CytoD treatment, and finally Relative Concavity and Aspect Ratio for compressive loading (Fig 4). With MCF7 cells, on the other hand, the top two parameters were Centre-Centroid mismatch and Projected Area for TNF-α treatment, SD of Normalized Intensities and Entropy for CytoD treatment, and finally SD of Normalized Intensities and Entropy for compressive loading (Fig 5). These differences indicate that in different cell types, different features are differentially sensitive to various input signals. This stresses on the need for measuring a number of nuclear morphometric as well as chromatin textural features for effective segregation of subtly altered nuclei from unaltered ones. These results highlight the potential of using this mobile system as a tool to detect subtle morphometric and textural nuclear changes induced by an altered physico-chemical extracellular microenvironment in pathological conditions such as cancer.

## Conclusions

We have developed a mobile microscope with sub-micron resolution and fluorescent capability. The resolution was suitable for measuring nuclear morphological as well as in-situ chromatin textural features. We demonstrated that these features can be used to segregate cells from different cell types into different regions of a combined feature space. We further assessed the sensitivity of the device by demonstrating its ability to detect extracellular cues-induced subtle nuclear and chromatin changes in a normal as well as a cancer cell line. This highlights the possibility of using this versatile mobile microscope for disease diagnosis where subtle changes in chromatin in response to pathological extracellular stimuli can be picked up at an early stage. Combining the progress in data analytics such as machine learning and communication systems with affordable and high quality mobile microscopes will open up a new paradigm with the possibility of remote expert consultation, thereby facilitating early therapeutic interventions.

## Supporting information

S1 Fig Schematic description of spatial correlation. Graphical description of the steps involved in extracting the textural correlation length-scale from nuclear images.
(PDF)

S2 Fig Discriminating between different populations of cells–Deltavision. a) Plot showing the percentage of total variance explained along each principle component for a dataset consisting of features from HME1, BJ and MCF7 nuclei, imaged under the conventional wide-field microscope. The red dots represent the percentage of dataset variance explained along individual principle components while the blue dots represent the cumulative sum. b,c) The loading coefficient of each parameter used to obtain the second and third principle components of the PCA for HME1, BJ and MCF7 nuclei, imaged under the conventional wide-field microscope (Fig 1B).
(PDF)

S3 Fig Characteristic spatial correlation (Radial average) in different types of cells–Deltavision. N: BJ = 300; HME1 = 389; MCF7 = 321.
(PDF)

S4 Fig Components of mobile microscope. 3D Tinkercad illustration of the mobile fluorescent microscope. a-c) 3 gear system for vertical movement to adjust focus. a) The lateral gear movable by the user (blue transparent) has a bore. The lateral gear is thus inserted in a shaft and hold in place with an external cap (grey transparent). b) The lateral gear interacts with other 2 gears (blue transparent) inserted in the same manner in the base of the microscope. One of these 2 gears drives the movement (c) of the screw (grey colour in all the Figs) inserted in the plane (yellow colour in all the Figs) where the XY stage lays. d and e) XY moving stage. d) X stage (red) and Y stage (blue) were designed as dovetail rail carriers where the carriers (filled colours) are inserted inside cases (transparent colours) where the screw is permanently inserted. e) In green colour is the sample holder, permanently joint to the Y carrier and the sample tray. The Y carrier carries directly the sample and the X carrier carries the X moving stage. f) Integration of the XYZ moving stage inside the box. (g) Final mobile microscope with the integration of the optical path components.
(PDF)

S5 Fig Discriminating between different populations of cells–Mobile Microscope. a) Plot showing the percentage of total variance explained along each principle component for a dataset consisting of features from HME1, BJ and MCF7 nuclei, imaged under the mobile microscope. The red dots represent the percentage of dataset variance explained along individual principle components while the blue dots represent the cumulative sum. b,c) The loading coefficient of each parameter used to obtain the second and third principle components of the PCA for HME1, BJ and MCF7 nuclei, imaged under the mobile microscope (Fig 3B).
(PDF)

S6 Fig Characteristic spatial correlation (Major-Minor axes) in different types of cells—Mobile Microscope. N: BJ = 54; HME1 = 83; MCF7 = 53.
(PDF)

S1 File Compilation of raw data. (PDF)
