# Supplementary figures and images for: Nuclear morphometrics and chromatin condensation patterns as disease biomarkers using a mobile microscope
Source: PLoS One. 2019 Jul 17;14(7):e0218757. doi: 10.1371/journal.pone.0218757 (PMC6636717; doi:10.1371/journal.pone.0218757)

**Supplementary Figure S1: Schematic description of spatial correlation**

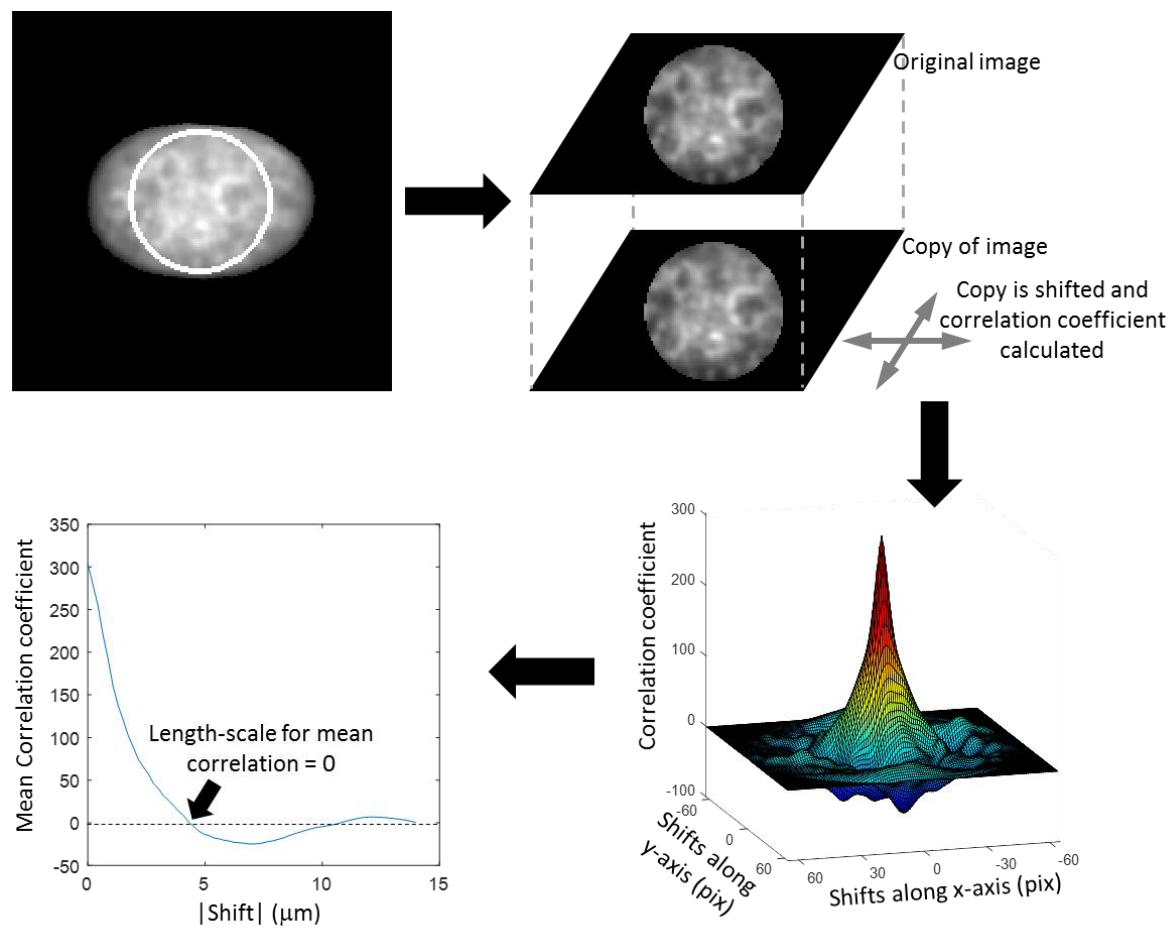

Supplement: S1 Fig — Graphical description of the steps involved in extracting the textural correlation length-scale from nuclear images. (PDF) [file pone.0218757.s001.pdf]

**Supplementary Figure S2: Discriminating between different populations of cells - Deltavision**

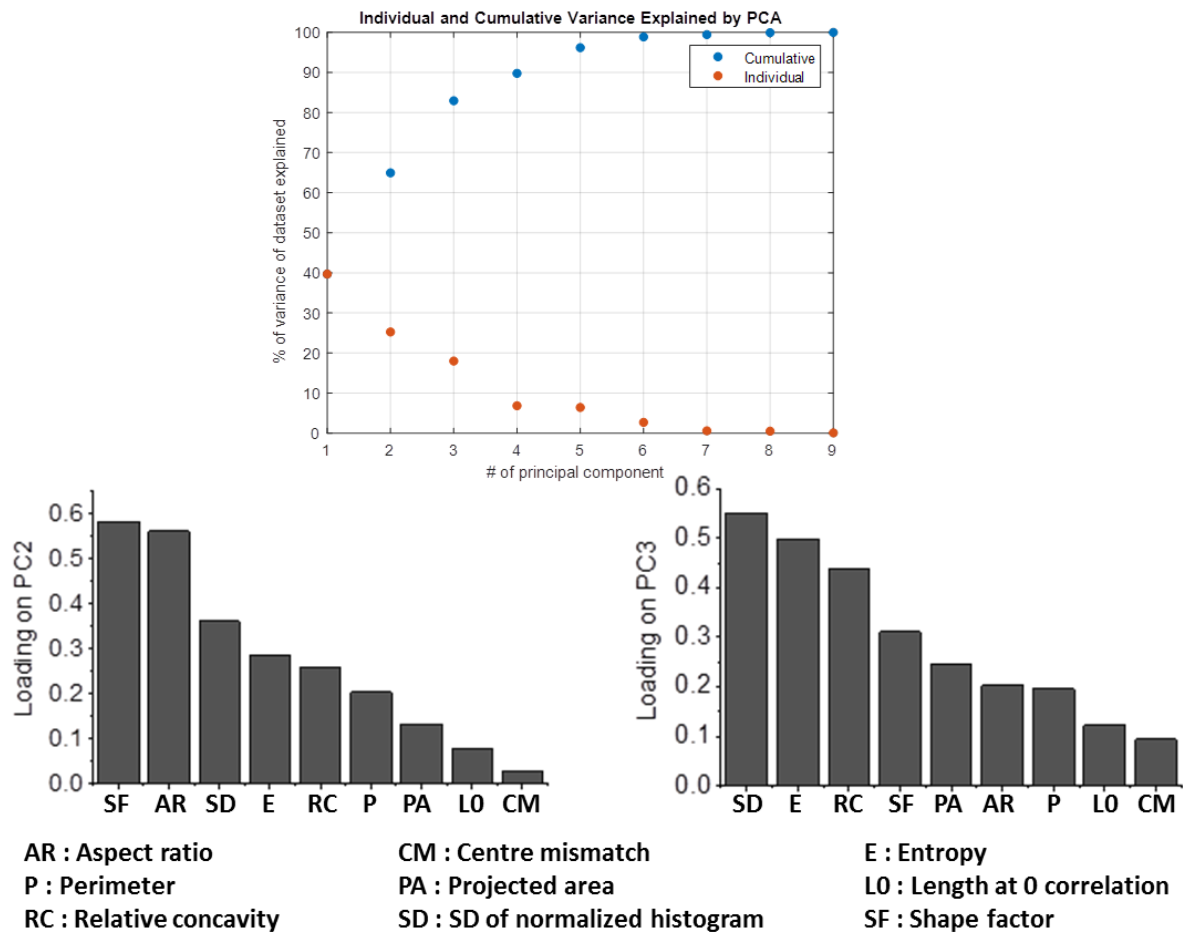

Supplement: S2 Fig — a) Plot showing the percentage of total variance explained along each principle component for a dataset consisting of features from HME1, BJ and MCF7 nuclei, imaged under the conventional wide-field microscope. The red dots represent the percentage of dataset variance explained along individual principle components while the blue dots represent the cumulative sum. b,c) The loading coefficient of each parameter used to obtain the second and third principle components of the PCA for HME1, BJ and MCF7 nuclei, imaged under the conventional wide-field microscope (Fig 1B). (PDF) [file pone.0218757.s002.pdf]

**Supplementary Figure S4: Components of mobile microscope**

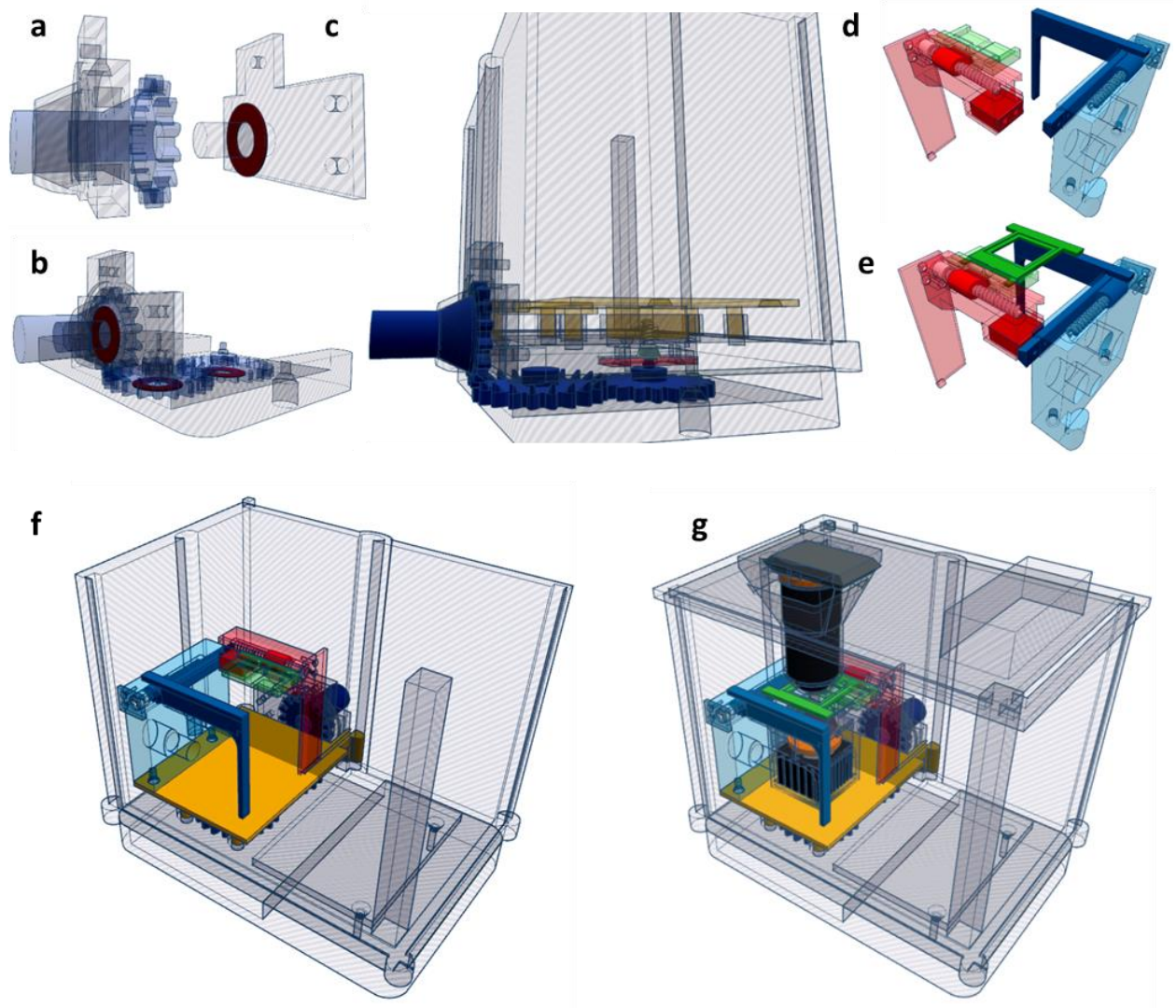

Supplement: S4 Fig — 3D Tinkercad illustration of the mobile fluorescent microscope. a-c) 3 gear system for vertical movement to adjust focus. a) The lateral gear movable by the user (blue transparent) has a bore. The lateral gear is thus inserted in a shaft and hold in place with an external cap (grey transparent). b) The lateral gear interacts with other 2 gears (blue transparent) inserted in the same manner in the base of the microscope. One of these 2 gears drives the movement (c) of the screw (grey colour in all the Figs) inserted in the plane (yellow colour in all the Figs) where the XY stage lays. d and e) XY moving stage. d) X stage (red) and Y stage (blue) were designed as dovetail rail carriers where the carriers (filled colours) are inserted inside cases (transparent colours) where the screw is permanently inserted. e) In green colour is the sample holder, permanently joint to the Y carrier and the sample tray. The Y carrier carries directly the sample and the X carrier carries the X moving stage. f) Integration of the XYZ moving stage inside the box. (g) Final mobile microscope with the integration of the optical path components. (PDF) [file pone.0218757.s004.pdf]
