# Supplementary material for: Nuclear morphometrics and chromatin condensation patterns as disease biomarkers using a mobile microscope
Source: PLoS One. 2019 Jul 17;14(7):e0218757. doi: 10.1371/journal.pone.0218757 (PMC6636717; doi:10.1371/journal.pone.0218757)

Supplementary Figure S3: Characteristic spatial correlation (Radial average) in different types of cells - Deltavision

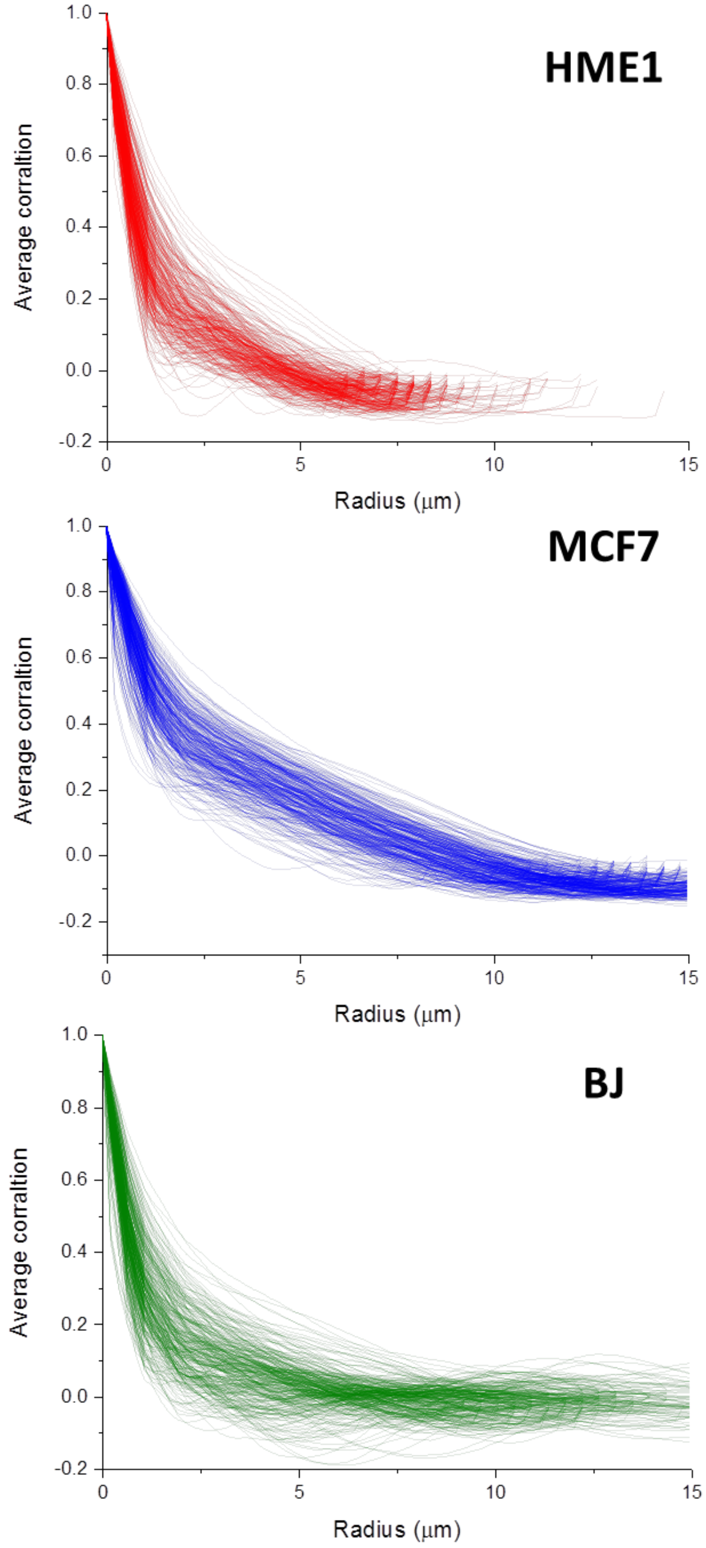

Supplement: S3 Fig — N: BJ = 300; HME1 = 389; MCF7 = 321. (PDF) [file pone.0218757.s003.pdf]
