# Supplementary material for: Nuclear morphometrics and chromatin condensation patterns as disease biomarkers using a mobile microscope
Source: PLoS One. 2019 Jul 17;14(7):e0218757. doi: 10.1371/journal.pone.0218757 (PMC6636717; doi:10.1371/journal.pone.0218757)
Supplement: S5 Fig — a) Plot showing the percentage of total variance explained along each principle component for a dataset consisting of features from HME1, BJ and MCF7 nuclei, imaged under the mobile microscope. The red dots represent the percentage of dataset variance explained along individual principle components while the blue dots represent the cumulative sum. b,c) The loading coefficient of each parameter used to obtain the second and third principle components of the PCA for HME1, BJ and MCF7 nuclei, imaged under the mobile microscope (Fig 3B). (PDF) [file pone.0218757.s005.pdf]

Supplementary Figure S5: Discriminating between different populations of cells – Mobile Microscope

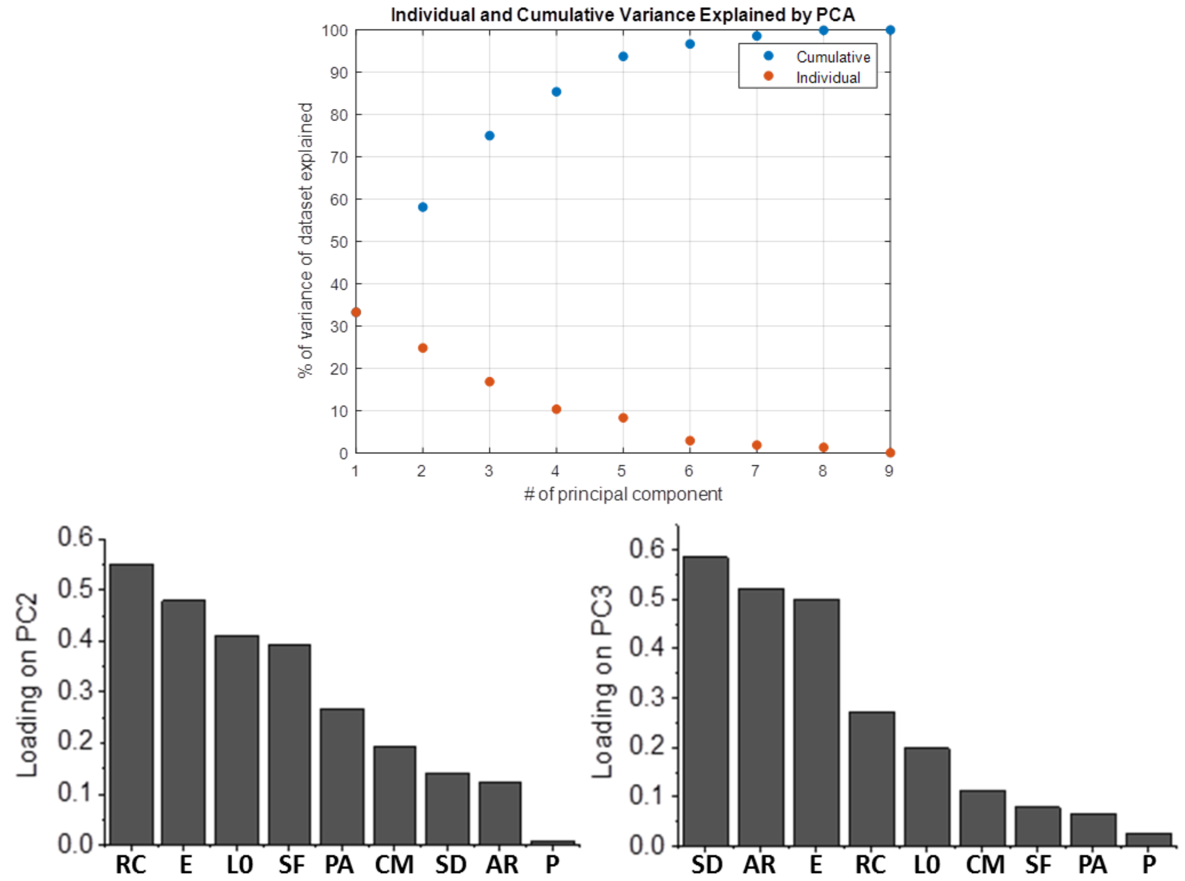

AR : Aspect ratio
P : Perimeter
RC : Relative concavity
CM : Centre mismatch
PA : Projected area
SD : SD of normalized histogram
E : Entropy
L0 : Length at 0 correlation
SF : Shape factor
