# Supplementary material for: Nuclear morphometrics and chromatin condensation patterns as disease biomarkers using a mobile microscope
Source: PLoS One. 2019 Jul 17;14(7):e0218757. doi: 10.1371/journal.pone.0218757 (PMC6636717; doi:10.1371/journal.pone.0218757)

Supplementary Figure S6: Characteristic spatial correlation (Major-Minor axes) in different types of cells - Mobile Microscope

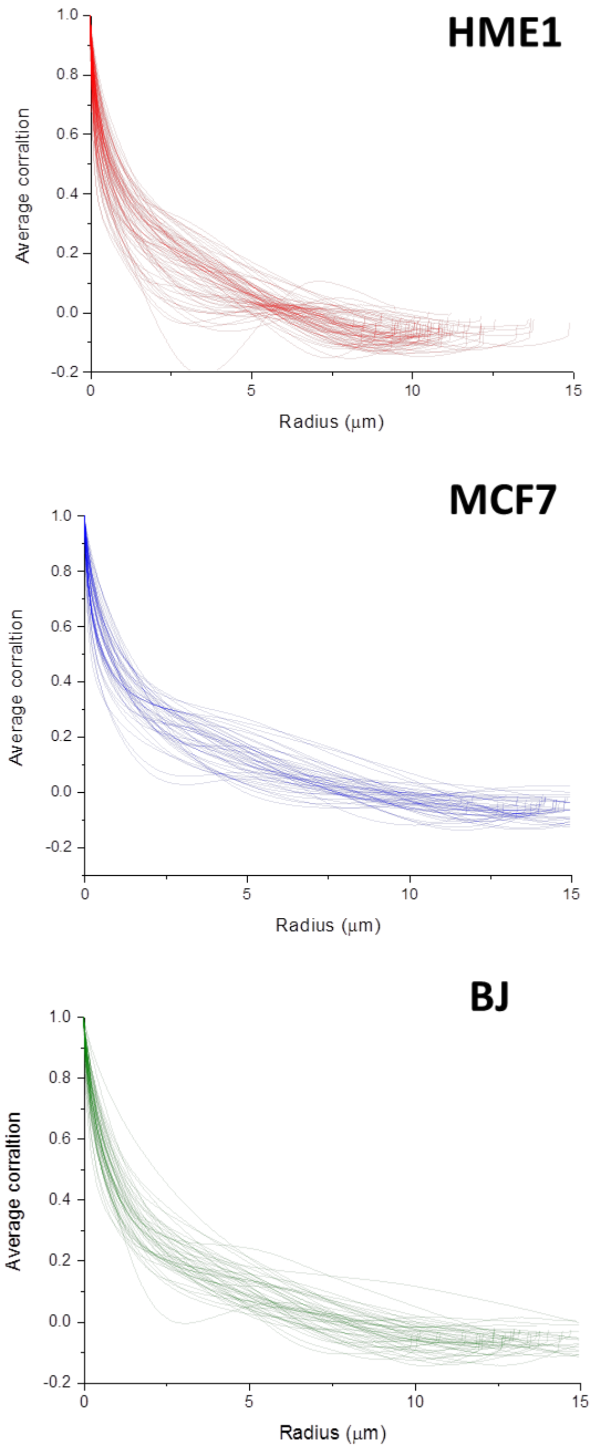

Supplement: S6 Fig — N: BJ = 54; HME1 = 83; MCF7 = 53. (PDF) [file pone.0218757.s006.pdf]
