# Supplementary material for: Nuclear morphometrics and chromatin condensation patterns as disease biomarkers using a mobile microscope
Source: PLoS One. 2019 Jul 17;14(7):e0218757. doi: 10.1371/journal.pone.0218757 (PMC6636717; doi:10.1371/journal.pone.0218757)
Supplement: S1 File — (PDF) [file pone.0218757.s007.pdf]

Features extracted from nuclei of different cell lines imaged on a conventional wide-field fluorescence microscope (DeltaVision)

Cell line	'Pro. Area(um^2)'	'Perimeter(um)'	'A.R.'	'Shape Factor'	'Centre Mismatch'	'S.D of Normalized Int'	'Entropy'	'Relative concavity'	'LenghtAt0Corr'
'Delta HME1'	80.70885	32.31149	1.36046	1.0294	0.11896	0.18014	7.47548	0.02458	4.085
'Delta HME1'	96.28668	34.71734	1.14842	0.99613	0.02175	0.17359	7.38473	0.01976	2.58
'Delta HME1'	87.4577	33.16332	1.27239	1.00071	0.02224	0.16922	7.31227	0.0207	3.87
'Delta HME1'	86.903	33.31468	1.31728	1.01631	0.06169	0.14457	7.17734	0.01879	3.655
'Delta HME1'	84.77665	32.42372	1.08098	0.98682	0.06977	0.17191	7.48544	0.01873	4.3
'Delta HME1'	121.1557	39.71372	1.3552	1.03592	0.10327	0.20732	7.72816	0.01835	4.945
'Delta HME1'	136.8722	41.70699	1.19992	1.01133	0.04472	0.20867	7.68581	0.02019	5.59
'Delta HME1'	91.8953	34.337	1.19855	1.02099	0.11993	0.18752	7.50623	0.02069	4.3
'Delta HME1'	87.55015	33.29318	1.24209	1.0075	0.0735	0.17803	7.46933	0.02119	3.87
'Delta HME1'	90.69345	33.82122	1.18679	1.00367	0.04331	0.1995	7.58183	0.02339	4.3
'Delta HME1'	83.85215	32.38631	1.13406	0.9954	0.09385	0.16667	7.33039	0.02473	5.16
'Delta HME1'	91.1557	34.2108	1.21907	1.02172	0.1673	0.17358	7.42841	0.02328	4.945
'Delta HME1'	104.1449	36.13634	1.15616	0.99779	0.05814	0.17354	7.45179	0.02001	4.515
'Delta HME1'	78.49005	31.25541	1.2733	0.99043	0.07741	0.16641	7.34397	0.01736	1.29
'Delta HME1'	85.74738	32.97133	1.24971	1.00889	0.17042	0.15891	7.35466	0.01956	4.085
'Delta HME1'	60.8321	28.38731	1.49264	1.05416	0.15665	0.21672	7.57612	0.03448	3.655
'Delta HME1'	88.47465	32.8219	1.11633	0.96894	0.09158	0.17061	7.37711	0.01289	4.515
'Delta HME1'	95.17727	34.49331	1.22698	0.99478	0.05227	0.14996	7.25454	0.01999	4.515
'Delta HME1'	82.74275	32.27602	1.22116	1.00189	0.10685	0.15837	7.30254	0.02452	4.3
'Delta HME1'	86.20963	32.66194	1.09405	0.98473	0.10578	0.18679	7.44206	0.01997	4.515
'Delta HME1'	89.6765	33.4497	1.2182	0.99288	0.09764	0.15078	7.23653	0.01722	4.73
'Delta HME1'	115.5163	38.24871	1.167	1.00781	0.25528	0.19263	7.60508	0.02	5.59
'Delta HME1'	70.72425	29.9609	1.26773	1.01002	0.04594	0.16437	7.35159	0.0261	3.655
'Delta HME1'	108.0741	36.97011	1.16287	1.0064	0.06836	0.16865	7.40825	0.0238	5.16

**'Delta HME1'**	72.2959	30.46593	1.32335	1.02166	0.06924	0.16975	7.42271	0.02676	3.225
**'Delta HME1'**	134.145	41.43695	1.26036	1.01857	0.16107	0.20297	7.63635	0.0186	5.375
**'Delta HME1'**	84.8691	33.03174	1.30427	1.02307	0.09197	0.15082	7.2275	0.02754	4.3
**'Delta HME1'**	136.3638	41.62529	1.268	1.01113	0.05861	0.21652	7.73161	0.01732	5.375
**'Delta HME1'**	88.33598	33.22739	1.20279	0.99459	0.128	0.16056	7.31916	0.02	4.73
**'Delta HME1'**	93.14338	34.23918	1.12701	1.00158	0.16582	0.19894	7.59195	0.02232	4.73
**'Delta HME1'**	97.4423	34.85967	1.09647	0.99241	0.04382	0.16802	7.40392	0.01999	4.3
**'Delta HME1'**	95.9631	35.05338	1.18842	1.01893	0.12703	0.1621	7.37312	0.02352	5.16
**'Delta HME1'**	82.69652	32.24398	1.31477	1.00046	0.07816	0.18211	7.46836	0.01757	3.655
**'Delta HME1'**	79.1372	31.43687	1.13618	0.99377	0.08362	0.19656	7.50587	0.02339	4.3
**'Delta HME1'**	94.02165	34.49159	1.16895	1.00691	0.23181	0.19545	7.63724	0.02586	3.01
**'Delta HME1'**	95.2235	35.05188	1.41558	1.02676	0.18112	0.18165	7.51364	0.01858	4.3
**'Delta HME1'**	211.9879	53.40664	1.42284	1.0707	0.28539	0.20759	7.72498	0.02217	6.45
**'Delta HME1'**	90.32365	33.75543	1.15164	1.00387	0.08487	0.16943	7.36881	0.02446	5.375
**'Delta HME1'**	78.53628	31.95953	1.27433	1.03495	0.11994	0.19744	7.57685	0.03136	3.87
**'Delta HME1'**	99.6611	35.42512	1.16344	1.00204	0.15086	0.16321	7.36335	0.02133	4.73
**'Delta HME1'**	90.50855	34.03106	1.2711	1.01824	0.06459	0.15598	7.26978	0.02198	4.3
**'Delta HME1'**	103.8676	36.39606	1.24802	1.01489	0.19865	0.19887	7.60836	0.02177	4.73
**'Delta HME1'**	88.1973	33.87991	1.44473	1.03567	0.04853	0.19673	7.63298	0.02354	3.87
**'Delta HME1'**	116.903	38.2399	1.09703	0.9954	0.07296	0.1725	7.40611	0.01672	5.805
**'Delta HME1'**	92.2651	33.89948	1.16876	0.99115	0.12973	0.18319	7.64702	0.0182	5.375
**'Delta HME1'**	79.09098	31.38183	1.11423	0.99088	0.14898	0.17017	7.45127	0.0178	3.44
**'Delta HME1'**	82.6503	32.48586	1.26875	1.01609	0.16419	0.17508	7.43393	0.02455	4.515
**'Delta HME1'**	91.20193	33.67889	1.17394	0.9897	0.09788	0.17991	7.47064	0.01792	4.085
**'Delta HME1'**	113.8522	37.74196	1.12862	0.99563	0.06526	0.18812	7.40917	0.02068	5.16
**'Delta HME1'**	134.5148	41.03318	1.17328	0.99607	0.12626	0.21842	7.71578	0.01556	5.59
**'Delta HME1'**	144.222	43.70219	1.22391	1.05382	0.35288	0.16944	7.40855	0.02864	6.45
**'Delta HME1'**	70.0771	29.52359	1.21119	0.98981	0.08636	0.18132	7.41924	0.02067	3.01
**'Delta HME1'**	117.6889	38.47984	1.17582	1.0012	0.03035	0.22071	7.77744	0.01699	5.16

**'Delta HME1'**	90.36987	33.73049	1.13924	1.00187	0.09648	0.18395	7.53807	0.0225	4.73
**'Delta HME1'**	103.2204	35.66678	1.11844	0.98074	0.02317	0.19786	7.58302	0.01673	4.73
**'Delta HME1'**	66.1942	28.78033	1.02782	0.99578	0.04748	0.1952	7.4479	0.02585	3.655
**'Delta HME1'**	90.0463	33.83713	1.33365	1.01184	0.13835	0.19022	7.57991	0.01963	4.085
**'Delta HME1'**	96.148	35.30773	1.42667	1.03179	0.18681	0.18316	7.40512	0.01979	3.87
**'Delta HME1'**	90.9708	34.99835	1.24995	1.07148	0.09314	0.18833	7.52684	0.03624	3.225
**'Delta HME1'**	103.7289	36.163	1.2247	1.00327	0.11929	0.15603	7.27635	0.01837	4.3
**'Delta HME1'**	92.31132	34.3297	1.11308	1.01596	0.13485	0.18927	7.55432	0.02633	4.73
**'Delta HME1'**	76.31748	30.92474	1.16493	0.99719	0.13302	0.19482	7.54279	0.02481	4.3
**'Delta HME1'**	69.3375	29.61238	1.15564	1.0064	0.10456	0.18935	7.52277	0.03226	3.87
**'Delta HME1'**	80.10792	31.66606	1.19587	0.9961	0.04726	0.17888	7.50706	0.01924	1.29
**'Delta HME1'**	149.5841	44.02233	1.09318	1.03098	0.04805	0.18339	7.49825	0.02764	5.805
**'Delta HME1'**	85.33135	32.65828	1.19924	0.99465	0.03015	0.16634	7.37789	0.01861	3.01
**'Delta HME1'**	89.63027	33.50474	1.11976	0.99666	0.09288	0.18081	7.5009	0.0217	4.515
**'Delta HME1'**	90.32365	33.46561	1.03079	0.9867	0.09894	0.16126	7.31955	0.01957	4.945
**'Delta HME1'**	75.90145	30.55838	1.16257	0.97904	0.03501	0.15777	7.35026	0.01853	4.73
**'Delta HME1'**	83.29745	32.59271	1.26006	1.01484	0.15412	0.16692	7.32133	0.02118	4.515
**'Delta HME1'**	66.564	28.84612	1.18077	0.99478	0.16597	0.18558	7.41857	0.02571	3.44
**'Delta HME1'**	93.1896	34.21596	1.24277	0.99972	0.0362	0.17088	7.37757	0.01659	3.87
**'Delta HME1'**	97.4423	34.67993	1.09917	0.9822	0.0283	0.16656	7.37823	0.01725	4.515
**'Delta HME1'**	73.31285	30.06044	1.18975	0.98085	0.07505	0.19983	7.55967	0.01674	3.44
**'Delta HME1'**	71.97232	30.09441	1.16845	1.00137	0.07914	0.1767	7.44533	0.01952	1.935
**'Delta HME1'**	66.79512	29.38663	1.41586	1.02883	0.08109	0.17032	7.42263	0.02431	3.44
**'Delta HME1'**	102.2035	35.9781	1.14244	1.00786	0.04588	0.17288	7.32123	0.02341	4.515
**'Delta HME1'**	107.9354	37.6637	1.34757	1.04586	0.02405	0.2053	7.64454	0.02911	2.795
**'Delta HME1'**	84.3144	32.71182	1.13457	1.00995	0.06586	0.15396	7.25791	0.0246	4.73
**'Delta HME1'**	154.1142	44.53596	1.26121	1.02416	0.04227	0.1902	7.61506	0.01768	5.805
**'Delta HME1'**	78.3976	31.51857	1.22233	1.00837	0.0187	0.20514	7.54735	0.02191	3.01
**'Delta HME1'**	95.2235	34.44171	1.14514	0.99132	0.1586	0.17089	7.35337	0.02045	4.73

**'Delta HME1'**	89.95385	33.91367	1.13108	1.01747	0.04164	0.19574	7.56389	0.03039	4.3
**'Delta HME1'**	110.8013	37.22983	1.17234	0.99547	0.27766	0.19355	7.57524	0.01923	5.375
**'Delta HME1'**	100.7705	35.68828	1.25899	1.00579	0.05244	0.19079	7.549	0.01978	4.3
**'Delta HME1'**	110.0617	38.59723	1.4022	1.07712	0.06244	0.16897	7.40159	0.03759	4.945
**'Delta HME1'**	86.76432	33.09947	1.23852	1.00483	0.05611	0.14447	7.13998	0.01779	4.085
**'Delta HME1'**	93.14338	34.77969	1.3286	1.03345	0.12743	0.21115	7.65663	0.02184	4.085
**'Delta HME1'**	75.76278	31.33023	1.43493	1.03101	0.05117	0.18793	7.53009	0.02149	3.44
**'Delta HME1'**	194.2375	50.78257	1.3408	1.05654	0.04901	0.19319	7.59746	0.02188	6.88
**'Delta HME1'**	102.2035	35.77729	1.16773	0.99664	0.22007	0.19315	7.5381	0.01733	4.945
**'Delta HME1'**	99.89222	35.22044	1.07057	0.98821	0.08164	0.17958	7.46037	0.02217	4.73
**'Delta HME1'**	70.86293	30.11935	1.28894	1.01874	0.07658	0.15527	7.22409	0.02481	2.795
**'Delta HME1'**	78.07403	31.35496	1.09112	1.00207	0.05384	0.1968	7.61119	0.02819	3.225
**'Delta HME1'**	67.99698	29.25849	1.15786	1.00185	0.10628	0.1939	7.47893	0.02583	4.3
**'Delta HME1'**	81.63335	32.23345	1.20266	1.01283	0.16179	0.18614	7.48146	0.02646	4.515
**'Delta HME1'**	69.3375	29.1927	1.18837	0.97807	0.05284	0.20416	7.59464	0.01832	3.655
**'Delta HME1'**	67.95075	29.56981	1.32501	1.02398	0.06474	0.1679	7.34507	0.02842	4.085
**'Delta HME1'**	82.04937	32.14444	1.27001	1.00214	0.0906	0.16861	7.31049	0.02042	4.085
**'Delta HME1'**	102.0186	35.82695	1.17835	1.00122	0.115	0.17957	7.45475	0.01824	4.515
**'Delta HME1'**	89.63027	33.3695	1.20215	0.98863	0.11681	0.17702	7.40122	0.01823	5.375
**'Delta HME1'**	116.2097	38.00684	1.10168	0.98917	0.09245	0.17211	7.5375	0.01605	5.375
**'Delta HME1'**	79.09098	31.8529	1.17149	1.02085	0.13049	0.18712	7.45296	0.02618	4.085
**'Delta HME1'**	83.06633	32.78105	1.38887	1.02946	0.0578	0.17621	7.41832	0.02284	3.655
**'Delta HME1'**	92.0802	34.09857	1.20696	1.00484	0.11841	0.19109	7.55955	0.0192	4.3
**'Delta HME1'**	81.63335	31.80495	1.1605	0.98608	0.09914	0.19534	7.51126	0.01834	4.3
**'Delta HME1'**	139.3222	42.39865	1.32479	1.02677	0.17004	0.22173	7.74194	0.02079	5.16
**'Delta HME1'**	89.53782	34.44515	1.25012	1.05448	0.03313	0.15908	7.43067	0.04487	4.3
**'Delta HME1'**	192.4347	49.26381	1.13147	1.0036	0.03553	0.18812	7.58445	0.01584	7.31
**'Delta HME1'**	106.3637	36.77102	1.27223	1.0116	0.10579	0.22479	7.74719	0.02002	4.73
**'Delta HME1'**	86.7181	33.33403	1.24088	1.01966	0.13571	0.16528	7.31115	0.02748	4.515

**'Delta HME1'**	90.32365	34.1377	1.29449	1.02673	0.09177	0.18282	7.48974	0.023	4.515
**'Delta HME1'**	104.8845	36.18966	1.1248	0.99368	0.16025	0.16598	7.39681	0.01945	5.805
**'Delta HME1'**	110.3853	37.31519	1.15433	1.00381	0.04397	0.17848	7.45123	0.0185	4.945
**'Delta HME1'**	80.70885	31.77635	1.09011	0.99558	0.0728	0.20525	7.57016	0.02294	4.515
**'Delta HME1'**	135.994	41.44383	1.2224	1.00506	0.07786	0.21916	7.68973	0.01868	5.375
**'Delta HME1'**	76.87217	31.21284	1.12202	1.00853	0.10508	0.17735	7.43217	0.02578	3.87
**'Delta HME1'**	102.4346	35.76288	1.11324	0.99359	0.17068	0.171	7.40455	0.02207	5.375
**'Delta HME1'**	124.4839	39.55011	1.22293	0.99994	0.09634	0.22512	7.74401	0.01894	5.16
**'Delta HME1'**	90.87835	33.78037	1.21113	0.99921	0.03647	0.17646	7.44061	0.01798	3.87
**'Delta HME1'**	174.3145	47.22819	1.13376	1.01826	0.33886	0.18767	7.60082	0.02027	6.665
**'Delta HME1'**	122.7274	40.48364	1.25396	1.06269	0.05621	0.14401	7.31865	0.03349	6.235
**'Delta HME1'**	95.73198	35.73988	1.23268	1.06179	0.02119	0.18344	7.47256	0.03674	1.505
**'Delta HME1'**	68.73658	29.33159	1.15958	0.99603	0.14062	0.18791	7.48916	0.01978	2.795
**'Delta HME1'**	107.0109	36.53452	1.21017	0.99259	0.07146	0.19603	7.52357	0.01531	4.945
**'Delta HME1'**	77.14952	30.79832	1.20271	0.97839	0.04517	0.17726	7.46436	0.01592	3.87
**'Delta HME1'**	83.62103	31.99329	1.01053	0.97408	0.08915	0.1668	7.38581	0.01631	4.73
**'Delta HME1'**	83.85215	33.29318	1.21058	1.05193	0.14944	0.17461	7.437	0.03613	4.515
**'Delta HME1'**	82.55785	31.70885	1.0939	0.96915	0.03794	0.20803	7.6587	0.01053	4.3
**'Delta HME1'**	77.98158	32.08574	1.32075	1.05056	0.01696	0.19423	7.55355	0.02654	3.655
**'Delta HME1'**	56.25583	27.35961	1.56549	1.05887	0.02438	0.17664	7.39397	0.02718	2.795
**'Delta HME1'**	129.1527	40.6206	1.07375	1.01667	0.21878	0.20031	7.58826	0.02478	5.805
**'Delta HME1'**	78.99853	31.79248	1.30175	1.01817	0.09961	0.19445	7.60002	0.02399	3.87
**'Delta HME1'**	90.64723	33.63632	1.21136	0.99324	0.01428	0.21112	7.61724	0.01901	4.3
**'Delta HME1'**	171.4023	49.95224	1.47726	1.15847	0.12789	0.1794	7.55046	0.03713	6.02
**'Delta HME1'**	65.77818	28.91363	1.145	1.01138	0.06849	0.18683	7.45382	0.02668	3.655
**'Delta HME1'**	83.34368	32.07155	1.10484	0.9821	0.0336	0.22167	7.70825	0.01797	4.73
**'Delta HME1'**	93.51318	33.83004	1.06074	0.97392	0.061	0.20136	7.51365	0.01557	4.73
**'Delta HME1'**	95.4084	34.92009	1.24868	1.01708	0.091	0.17591	7.43629	0.0255	1.29
**'Delta HME1'**	115.1465	37.84688	1.18227	0.98992	0.02548	0.19511	7.56564	0.01542	3.225

**'Delta HME1'**	102.8969	36.38187	1.33051	1.02367	0.07226	0.17428	7.47426	0.01938	4.085
**'Delta HME1'**	99.33752	34.99469	1.10174	0.98103	0.09563	0.20087	7.63423	0.01512	4.73
**'Delta HME1'**	85.33135	32.42007	1.02769	0.98019	0.07561	0.14444	7.22897	0.02069	4.945
**'Delta HME1'**	70.03087	29.46661	1.13909	0.98665	0.0954	0.1842	7.52527	0.02132	3.44
**'Delta HME1'**	190.5395	49.6994	1.29278	1.03159	0.29353	0.20037	7.66483	0.02114	6.665
**'Delta HME1'**	74.6996	31.70347	1.45858	1.07074	0.09021	0.18678	7.5043	0.03924	3.655
**'Delta HME1'**	85.70115	33.11709	1.14918	1.01838	0.12239	0.14785	7.19638	0.02112	4.73
**'Delta HME1'**	83.15878	32.14981	1.09881	0.9891	0.06542	0.18223	7.4877	0.01908	4.3
**'Delta HME1'**	92.6349	34.39205	1.30067	1.01609	0.18476	0.16781	7.4435	0.01957	4.515
**'Delta HME1'**	104.7459	36.06324	1.15221	0.98806	0.26644	0.22825	7.72688	0.01905	5.16
**'Delta HME1'**	82.04937	31.949	1.13681	0.98999	0.1054	0.17303	7.44399	0.01879	4.515
**'Delta HME1'**	80.2466	31.71422	1.21456	0.99741	0.10764	0.19644	7.53361	0.02142	2.58
**'Delta HME1'**	106.5024	36.80843	1.15384	1.01234	0.07024	0.19062	7.57943	0.02166	5.16
**'Delta HME1'**	68.18188	29.51133	1.33381	1.01648	0.04944	0.18268	7.43089	0.01928	3.44
**'Delta HME1'**	113.6673	39.3192	1.27973	1.08234	0.06033	0.1954	7.57359	0.0387	5.375
**'Delta HME1'**	262.3269	59.79537	1.57737	1.08463	0.07773	0.18262	7.62613	0.0202	4.085
**'Delta HME1'**	100.6781	35.67753	1.09988	1.00611	0.04307	0.17389	7.43413	0.02507	4.73
**'Delta HME1'**	100.0771	36.25717	1.38026	1.04531	0.02941	0.18447	7.37211	0.02257	3.655
**'Delta HME1'**	105.6241	36.13634	1.10875	0.98382	0.08086	0.17038	7.41028	0.01678	4.73
**'Delta HME1'**	68.87525	29.40254	1.13704	0.99884	0.02043	0.14281	7.05424	0.02551	3.87
**'Delta HME1'**	104.3298	35.91231	1.08869	0.98371	0.13019	0.1694	7.42586	0.01656	5.16
**'Delta HME1'**	93.83675	34.30497	1.18914	0.998	0.07048	0.20052	7.59483	0.01742	4.085
**'Delta HME1'**	63.4207	27.93925	1.13401	0.97946	0.06919	0.19562	7.5196	0.02	4.085
**'Delta HME1'**	182.635	48.848	1.30061	1.03968	0.06529	0.19208	7.6361	0.01985	6.235
**'Delta HME1'**	69.79975	29.30493	1.20071	0.97908	0.07871	0.20769	7.63164	0.01307	3.44
**'Delta HME1'**	84.17573	32.91091	1.3566	1.02396	0.01688	0.16555	7.36863	0.02307	3.87
**'Delta HME1'**	105.6704	36.34081	1.04419	0.99455	0.19066	0.15503	7.22397	0.01888	4.945
**'Delta HME1'**	108.3976	36.77274	1.14184	0.99271	0.11367	0.16439	7.40098	0.0221	5.805
**'Delta HME1'**	69.75352	29.54487	1.05435	0.99584	0.03358	0.19745	7.52978	0.02645	3.655

**'Delta HME1'**	68.82903	31.3513	1.51073	1.1364	0.03352	0.17021	7.35301	0.05038	4.515
**'Delta HME1'**	95.73198	34.81517	1.11639	1.00756	0.15084	0.18767	7.51267	0.0208	4.945
**'Delta HME1'**	62.6811	27.94635	1.12732	0.99152	0.04541	0.17564	7.31834	0.02796	3.87
**'Delta HME1'**	113.7597	37.78109	1.19472	0.99851	0.0737	0.1934	7.50932	0.01874	5.16
**'Delta HME1'**	106.9184	36.29286	1.0839	0.98035	0.19457	0.19853	7.52037	0.01491	4.945
**'Delta HME1'**	65.8244	28.87278	1.22535	1.00781	0.07139	0.19512	7.5418	0.02599	4.085
**'Delta HME1'**	92.58867	34.22671	1.22609	1.00684	0.10616	0.15042	7.17022	0.02197	5.16
**'Delta HME1'**	120.5548	39.57892	1.18398	1.03403	0.21345	0.18847	7.54127	0.02614	5.59
**'Delta HME1'**	61.71037	28.16328	1.45048	1.02282	0.00603	0.16924	7.35347	0.02341	3.01
**'Delta HME1'**	72.57325	30.00368	1.10928	0.9871	0.15741	0.174	7.4081	0.01814	3.87
**'Delta HME1'**	111.2174	38.18465	1.29753	1.04327	0.1968	0.1696	7.51355	0.02552	4.945
**'Delta HME1'**	86.57943	33.05668	1.2195	1.00437	0.17759	0.1611	7.31179	0.02142	3.225
**'Delta HME1'**	75.809	30.90883	1.2128	1.00285	0.0837	0.18865	7.49419	0.02148	4.085
**'Delta HME1'**	83.43613	33.16676	1.50573	1.04916	0.07117	0.17969	7.56208	0.02115	3.225
**'Delta HME1'**	88.10485	33.70899	1.32243	1.02632	0.19262	0.22584	7.58432	0.02506	3.87
**'Delta HME1'**	91.98775	33.85132	1.03814	0.99131	0.19941	0.16833	7.40216	0.02115	4.945
**'Delta HME1'**	101.3714	36.09377	1.19215	1.02268	0.12762	0.17508	7.45404	0.0236	4.73
**'Delta HME1'**	87.8275	33.43895	1.22178	1.01313	0.12467	0.15699	7.27824	0.02414	4.515
**'Delta HME1'**	93.32828	35.0321	1.47607	1.04643	0.04909	0.17626	7.47501	0.02228	3.87
**'Delta HME1'**	82.97388	32.72236	1.41121	1.02692	0.13118	0.16453	7.32156	0.02126	4.085
**'Delta HME1'**	136.7336	41.96499	1.01891	1.02492	0.04821	0.22079	7.67556	0.02441	5.805
**'Delta HME1'**	96.65648	34.6337	1.06795	0.98755	0.09115	0.18702	7.48021	0.01923	4.73
**'Delta HME1'**	80.84753	31.691	1.07014	0.98854	0.02579	0.20194	7.58125	0.024	4.945
**'Delta HME1'**	103.7289	36.01895	1.07992	0.9953	0.03075	0.15114	7.21679	0.02094	4.945
**'Delta HME1'**	97.8121	34.84548	1.10087	0.98785	0.07946	0.19371	7.59298	0.01673	4.73
**'Delta HME1'**	162.1111	45.29362	1.16074	1.00705	0.24722	0.18714	7.60115	0.01765	6.45
**'Delta HME1'**	94.80747	35.31311	1.39871	1.04669	0.07029	0.1885	7.53002	0.02473	4.515
**'Delta HME1'**	63.09712	27.88593	1.10754	0.98073	0.13101	0.17245	7.32003	0.0208	3.87
**'Delta HME1'**	98.87528	35.17787	1.21465	0.99596	0.03916	0.18633	7.51328	0.0161	4.73

**'Delta HME1'**	163.1743	45.87326	1.16549	1.02626	0.03957	0.20308	7.67413	0.02351	6.02
**'Delta HME1'**	106.3637	36.65557	1.09457	1.00526	0.09619	0.17377	7.39073	0.02293	6.235
**'Delta HME1'**	86.1634	32.68516	1.13873	0.98666	0.02045	0.16482	7.33549	0.01791	4.3
**'Delta HME1'**	73.12795	30.74522	1.40061	1.02864	0.10516	0.14797	7.12051	0.02225	3.655
**'Delta HME1'**	91.61795	34.34045	1.14191	1.02429	0.12426	0.1772	7.4693	0.03033	4.73
**'Delta HME1'**	85.47003	32.89135	1.00725	1.00726	0.04312	0.15863	7.27144	0.02838	4.945
**'Delta HME1'**	109.7382	36.96301	1.19756	0.99076	0.05708	0.19613	7.47977	0.0186	4.3
**'Delta HME1'**	98.82905	34.82226	1.03762	0.97638	0.13471	0.17758	7.44479	0.01701	5.16
**'Delta HME1'**	88.98313	33.54043	1.10518	1.00605	0.06037	0.19242	7.55227	0.02135	4.73
**'Delta HME1'**	79.64567	31.59146	1.1954	0.99717	0.12107	0.18364	7.45106	0.01767	4.085
**'Delta HME1'**	97.16495	35.5797	1.2649	1.03678	0.08914	0.21674	7.65068	0.0264	4.3
**'Delta HME1'**	60.73965	27.86293	1.26195	1.01712	0.10862	0.18331	7.40343	0.02305	3.225
**'Delta HME1'**	96.148	34.54491	1.21699	0.98768	0.16851	0.17649	7.41279	0.01562	4.945
**'Delta HME1'**	202.2344	51.65375	1.07941	1.04988	0.24275	0.19035	7.6153	0.02886	4.73
**'Delta HME1'**	105.9015	36.76758	1.19149	1.01582	0.09374	0.19903	7.62023	0.02261	4.945
**'Delta HME1'**	85.10022	32.72429	1.15378	1.00138	0.07085	0.20052	7.57739	0.02127	4.945
**'Delta HME1'**	95.13105	34.26756	1.12903	0.98228	0.01383	0.16088	7.31021	0.01625	4.085
**'Delta HME1'**	101.0941	35.65087	1.12838	1.00047	0.07738	0.1721	7.4404	0.0206	4.945
**'Delta HME1'**	95.2235	34.90224	1.27275	1.01801	0.1491	0.20072	7.66675	0.02184	4.515
**'Delta HME1'**	102.712	35.76482	1.16225	0.99102	0.06498	0.19047	7.53864	0.01638	4.73
**'Delta HME1'**	108.4901	37.00236	1.12162	1.00429	0.10373	0.18521	7.5358	0.01963	4.945
**'Delta HME1'**	79.04475	31.62177	1.24625	1.00668	0.10278	0.16103	7.12427	0.02453	3.655
**'Delta HME1'**	100.1696	35.32536	1.22106	0.99135	0.08486	0.18579	7.50555	0.01723	4.515
**'Delta HME1'**	89.81517	34.00096	1.30936	1.02429	0.0611	0.18007	7.48818	0.02116	4.085
**'Delta HME1'**	74.6996	31.01181	1.24114	1.02453	0.07472	0.18346	7.41591	0.02709	3.44
**'Delta HME1'**	87.36525	33.17751	1.33518	1.00263	0.11338	0.18164	7.48187	0.01818	3.655
**'Delta HME1'**	85.00777	33.43723	1.39716	1.04663	0.16174	0.19704	7.53989	0.02647	2.795
**'Delta HME1'**	187.9509	48.56871	1.11065	0.99876	0.20516	0.21184	7.63147	0.01669	6.88
**'Delta HME1'**	206.0248	51.46885	1.15062	1.0232	0.165	0.24694	7.8479	0.02302	7.095

**'Delta HME1'**	85.7936	32.92682	1.23548	1.00562	0.07	0.19162	7.6412	0.01955	4.085
**'Delta HME1'**	102.9893	36.1329	1.08607	1.0088	0.02864	0.15107	7.27198	0.02452	2.365
**'Delta HME1'**	109.1372	37.54459	1.30029	1.02781	0.08629	0.17114	7.42206	0.02919	4.73
**'Delta HME1'**	96.148	34.95212	1.16885	1.01111	0.11109	0.1845	7.48519	0.0253	4.515
**'Delta HME1'**	100.1696	35.30773	1.16682	0.99036	0.17878	0.19337	7.55007	0.01768	5.805
**'Delta HME1'**	77.658	31.5792	1.27942	1.02189	0.04049	0.18301	7.5187	0.02212	3.655
**'Delta HME1'**	100.0309	35.36277	1.18303	0.99483	0.0673	0.18552	7.49034	0.01993	4.515
**'Delta HME1'**	109.3684	38.09219	1.50873	1.05577	0.21827	0.22226	7.67282	0.0207	4.3
**'Delta HME1'**	90.27742	33.37488	1.01719	0.98186	0.18574	0.20198	7.57121	0.02154	4.73
**'Delta HME1'**	51.30975	25.30937	1.30823	0.99347	0.07315	0.21007	7.59597	0.02289	2.795
**'Delta HME1'**	150.6011	44.12015	1.25147	1.02858	0.06044	0.22227	7.69171	0.02221	5.375
**'Delta HME1'**	91.43305	34.3813	1.15717	1.0288	0.10716	0.17192	7.42179	0.03039	4.945
**'Delta HME1'**	62.6811	28.3413	1.3878	1.01975	0.05193	0.17127	7.30684	0.01739	3.01
**'Delta HME1'**	68.55168	29.04177	1.05343	0.97908	0.08722	0.1703	7.3416	0.01723	3.655
**'Delta HME1'**	107.0109	36.57537	1.2295	0.99481	0.16463	0.1877	7.53918	0.01948	5.59
**'Delta HME1'**	148.6596	43.25929	1.06542	1.00174	0.20723	0.18345	7.54766	0.02249	6.45
**'Delta HME1'**	111.8645	38.02275	1.02294	1.02845	0.17926	0.18569	7.53172	0.03277	5.805
**'Delta HME1'**	132.2035	41.16648	1.25296	1.02008	0.08574	0.17302	7.48304	0.01786	5.805
**'Delta HME1'**	110.3853	37.59985	1.1712	1.01918	0.15954	0.20872	7.67983	0.02491	6.02
**'Delta HME1'**	72.01855	29.90048	1.12219	0.98788	0.0932	0.18048	7.51692	0.01827	4.085
**'Delta HME1'**	100.262	35.58508	1.12661	1.00505	0.05444	0.16799	7.38777	0.02297	5.16
**'Delta HME1'**	104.4685	35.88909	1.11971	0.98114	0.09284	0.18764	7.55937	0.01611	4.515
**'Delta HME1'**	91.01703	34.26412	1.3076	1.02647	0.10511	0.16193	7.38739	0.02573	3.87
**'Delta HME1'**	105.6704	36.63944	1.14535	1.01096	0.08257	0.17979	7.44093	0.02474	4.3
**'Delta HME1'**	93.28205	34.23187	1.0854	0.99966	0.12831	0.17468	7.47155	0.02181	4.515
**'Delta HME1'**	91.3406	33.69673	1.05643	0.98924	0.14237	0.17977	7.43726	0.02227	4.945
**'Delta HME1'**	94.99237	34.30669	1.12128	0.98596	0.10584	0.19861	7.57212	0.01956	4.73
**'Delta HME1'**	121.202	38.95994	1.03558	0.99659	0.10637	0.173	7.40167	0.01981	5.805
**'Delta HME1'**	100.5856	36.28383	1.14606	1.04155	0.13602	0.17005	7.36111	0.03289	4.945

**'Delta HME1'**	225.3007	53.15617	1.01531	0.99801	0.10258	0.21289	7.7043	0.01635	7.74
**'Delta HME1'**	114.5918	39.85627	1.5334	1.10314	0.18773	0.21114	7.6386	0.03541	4.73
**'Delta HME1'**	108.5363	37.59791	1.15844	1.03644	0.15084	0.18541	7.49896	0.03295	4.945
**'Delta HME1'**	113.2975	37.96599	1.24256	1.01242	0.05331	0.17672	7.39757	0.02156	5.16
**'Delta HME1'**	84.1295	32.67269	1.16457	1.00974	0.14215	0.19226	7.53156	0.02203	4.3
**'Delta HME1'**	98.13568	35.00716	1.15771	0.99375	0.10511	0.20456	7.62676	0.01985	4.73
**'Delta HME1'**	61.15568	28.50836	1.38864	1.05754	0.14655	0.2341	7.69955	0.03501	3.87
**'Delta HME1'**	114.0833	38.17239	1.186	1.0164	0.06135	0.17929	7.50218	0.02063	5.375
**'Delta HME1'**	98.96773	34.91471	1.16664	0.9802	0.03533	0.17262	7.44301	0.01473	4.945
**'Delta HME1'**	94.25278	34.88461	1.1383	1.02746	0.03709	0.18003	7.42443	0.02951	4.3
**'Delta HME1'**	83.3899	32.35428	1.05673	0.99894	0.02138	0.16913	7.43802	0.02486	4.515
**'Delta HME1'**	127.1188	41.06328	1.39644	1.05557	0.05567	0.18469	7.50134	0.03067	5.16
**'Delta HME1'**	155.5934	44.3173	1.14201	1.00449	0.21807	0.18122	7.39225	0.01866	5.59
**'Delta HME1'**	89.26048	33.79284	1.09861	1.01808	0.06184	0.18608	7.50294	0.02573	4.73
**'Delta HME1'**	87.78127	33.28243	1.08094	1.0042	0.0716	0.17637	7.46106	0.02565	4.73
**'Delta HME1'**	122.6812	39.28717	1.05858	1.00118	0.10209	0.18153	7.49007	0.02391	6.02
**'Delta HME1'**	113.3899	37.3756	1.06939	0.98037	0.1121	0.19863	7.60615	0.01248	5.375
**'Delta HME1'**	105.1157	37.08922	1.44594	1.0414	0.19419	0.1956	7.5985	0.01856	3.01
**'Delta HME1'**	119.769	39.22482	1.26233	1.02227	0.12528	0.17402	7.48914	0.02042	5.375
**'Delta HME1'**	66.42533	28.56511	1.0488	0.97753	0.07218	0.14483	7.0337	0.01911	2.15
**'Delta HME1'**	98.08945	35.1869	1.13091	1.00445	0.05899	0.16638	7.3616	0.02167	5.375
**'Delta HME1'**	87.36525	33.11709	1.05541	0.99898	0.06235	0.20312	7.59252	0.02225	4.73
**'Delta HME1'**	93.05093	34.29422	1.22862	1.0058	0.07528	0.19841	7.57164	0.01853	4.3
**'Delta HME1'**	63.9754	28.42644	1.31518	1.00513	0.09777	0.19836	7.55257	0.02191	3.655
**'Delta HME1'**	86.5332	33.86745	1.08262	1.05481	0.16562	0.19206	7.54481	0.03256	4.73
**'Delta HME1'**	66.65645	30.83229	1.81528	1.13491	0.0233	0.17267	7.38131	0.04186	1.935
**'Delta HME1'**	109.1372	37.59275	1.11778	1.03045	0.04426	0.15775	7.34033	0.028	5.375
**'Delta HME1'**	108.3976	37.2423	1.05764	1.01822	0.06838	0.13776	7.13738	0.02414	5.375
**'Delta HME1'**	116.1172	38.56692	1.29974	1.01935	0.13935	0.19704	7.56938	0.01837	4.085

'Delta HME1'	75.34675	31.1363	1.41732	1.0239	0.01592	0.19	7.50543	0.0222	3.655
'Delta HME1'	80.84753	32.00404	1.31936	1.00817	0.06491	0.18503	7.46848	0.01962	4.515
'Delta HME1'	78.72118	31.74088	1.3698	1.01844	0.15579	0.18896	7.48857	0.02407	2.795
'Delta HME1'	86.20963	33.01411	1.09665	1.00608	0.03689	0.20101	7.59356	0.02151	4.3
'Delta HME1'	75.53165	31.47944	1.49436	1.04404	0.01969	0.16184	7.32833	0.02331	3.44
'Delta HME1'	118.2436	38.55638	1.08861	1.00047	0.11116	0.18046	7.50694	0.02142	5.375
'Delta HME1'	185.316	48.98302	1.30175	1.03031	0.19684	0.15798	7.39463	0.01908	6.45
'Delta HME1'	166.41	46.17555	1.19917	1.01961	0.04949	0.19061	7.62493	0.01961	6.02
'Delta HME1'	79.46077	31.40118	1.08098	0.98748	0.06483	0.1412	7.02339	0.02274	4.3
'Delta HME1'	108.3514	36.95248	1.14754	1.00287	0.13648	0.20969	7.62705	0.02211	4.945
'Delta HME1'	94.76125	34.26928	1.13973	0.98621	0.12151	0.19762	7.4767	0.01773	4.515
'Delta HME1'	92.0802	33.61675	1.01799	0.97664	0.01857	0.16869	7.45437	0.01824	4.73
'Delta HME1'	95.13105	34.72272	1.18132	1.00854	0.09516	0.22172	7.64987	0.02557	4.515
'Delta HME1'	175.9786	47.58724	1.19044	1.02403	0.38439	0.18363	7.56863	0.02134	6.88
'Delta HME1'	105.2081	36.67878	1.27892	1.01759	0.15249	0.19961	7.63912	0.01514	4.3
'Delta HME1'	75.9939	31.33904	1.15376	1.02845	0.08214	0.17414	7.45646	0.03009	2.58
'Delta HME1'	88.98313	33.34994	1.09557	0.99466	0.10338	0.17999	7.46146	0.02532	4.73
'Delta MCF7'	220.7706	55.35562	1.55442	1.10452	0.22006	0.23494	7.7984	0.0189	6.235
'Delta MCF7'	321.6798	63.43188	1.17474	0.99536	0.1019	0.18775	7.56867	0.0094	9.46
'Delta MCF7'	364.2992	68.85676	1.31672	1.03568	0.63634	0.21367	7.74653	0.01451	10.32
'Delta MCF7'	412.5119	74.7512	1.53143	1.07793	0.26806	0.22283	7.77285	0.01272	8.385
'Delta MCF7'	299.3531	62.4489	1.46022	1.03671	0.23247	0.19278	7.61459	0.00872	8.17
'Delta MCF7'	299.2607	61.77466	1.23697	1.01476	0.15191	0.21092	7.70493	0.01281	8.6
'Delta MCF7'	281.4178	61.27844	1.51356	1.06183	0.45025	0.20439	7.66196	0.01281	7.095
'Delta MCF7'	393.2361	72.541	1.5254	1.06489	0.41698	0.22174	7.74288	0.01196	9.46
'Delta MCF7'	354.4071	70.89453	1.39353	1.12853	0.04232	0.21405	7.75431	0.03838	9.89
'Delta MCF7'	231.9571	54.54851	1.14224	1.02082	0.17835	0.22554	7.75928	0.01723	7.74
'Delta MCF7'	403.2669	72.61195	1.32645	1.04043	0.44288	0.19196	7.65156	0.01032	10.535
'Delta MCF7'	295.5164	62.69013	1.49989	1.0583	0.29218	0.20544	7.72252	0.01388	7.525

**'Delta MCF7'**	407.2423	72.81835	1.35352	1.03614	0.38243	0.209	7.73246	0.01311	10.32
**'Delta MCF7'**	303.8832	62.88234	1.27085	1.03548	0.24223	0.2038	7.71097	0.00964	8.6
**'Delta MCF7'**	431.8802	74.32464	1.12719	1.01787	0.21229	0.2028	7.64229	0.01279	12.04
**'Delta MCF7'**	383.76	71.40967	1.26164	1.05741	0.2799	0.20546	7.70306	0.01577	9.46
**'Delta MCF7'**	257.7968	57.46821	1.2744	1.01945	0.33192	0.20395	7.67634	0.0131	7.525
**'Delta MCF7'**	280.6782	60.39479	1.38234	1.03414	0.75465	0.21255	7.7429	0.01011	6.88
**'Delta MCF7'**	294.6844	62.4274	1.51996	1.05241	0.32392	0.19723	7.68023	0.01055	7.525
**'Delta MCF7'**	381.2176	71.77474	1.56245	1.07538	0.5889	0.22798	7.76838	0.01174	8.17
**'Delta MCF7'**	323.7137	64.95408	1.20801	1.03715	0.5972	0.22083	7.79882	0.01533	8.385
**'Delta MCF7'**	306.518	64.19126	1.55965	1.06976	0.28616	0.22642	7.78153	0.01148	7.74
**'Delta MCF7'**	275.8246	59.1379	1.04319	1.00899	0.242	0.2328	7.6124	0.01339	9.03
**'Delta MCF7'**	421.2022	72.84157	1.18225	1.00244	0.10555	0.2084	7.66414	0.00903	10.105
**'Delta MCF7'**	302.3577	62.48932	1.35091	1.02773	0.17567	0.21168	7.72082	0.01223	7.74
**'Delta MCF7'**	355.9787	72.74912	1.35182	1.1831	0.65963	0.21642	7.76438	0.0247	9.03
**'Delta MCF7'**	315.6705	66.40662	1.38972	1.11168	0.52634	0.22483	7.76389	0.03559	9.03
**'Delta MCF7'**	325.2853	65.65627	1.26678	1.05458	0.31493	0.2015	7.67431	0.01882	8.6
**'Delta MCF7'**	332.82	66.29267	1.49716	1.05078	0.27347	0.20509	7.65546	0.0114	7.31
**'Delta MCF7'**	344.6074	67.22276	1.22023	1.04351	0.19835	0.20156	7.63394	0.01753	9.89
**'Delta MCF7'**	177.8738	49.91999	1.50905	1.11488	0.3236	0.22675	7.76709	0.02533	5.59
**'Delta MCF7'**	214.1142	52.76702	1.41394	1.03483	0.11382	0.20676	7.67946	0.01258	6.235
**'Delta MCF7'**	197.2421	51.12743	1.46185	1.05463	0.16328	0.22114	7.74809	0.01705	5.59
**'Delta MCF7'**	363.1436	68.68648	1.2763	1.03384	0.24761	0.19591	7.67893	0.01282	10.32
**'Delta MCF7'**	203.2513	51.79952	1.18216	1.05053	0.05114	0.21506	7.73347	0.02028	7.31
**'Delta MCF7'**	344.6998	69.48456	1.55164	1.11462	0.28814	0.21284	7.69309	0.03068	8.385
**'Delta MCF7'**	295.7013	63.45854	1.28839	1.08372	0.31392	0.21089	7.73268	0.03732	9.03
**'Delta MCF7'**	255.7167	58.45334	1.45901	1.06329	0.11225	0.23048	7.7639	0.02365	7.31
**'Delta MCF7'**	359.0296	68.25562	1.38775	1.03261	0.66104	0.19997	7.69054	0.01233	9.03
**'Delta MCF7'**	322.4194	63.47101	1.01484	0.99431	0.15617	0.18615	7.59834	0.01036	9.675
**'Delta MCF7'**	233.0665	54.96647	1.13437	1.03159	0.16194	0.2256	7.78773	0.01773	7.525

**'Delta MCF7'**	245.7321	57.30094	1.31978	1.06329	0.19219	0.21814	7.74735	0.03081	8.385
**'Delta MCF7'**	332.1729	65.93706	1.31288	1.04156	0.21616	0.16663	7.48733	0.01332	9.675
**'Delta MCF7'**	462.4349	76.02787	1.065	0.99468	0.43917	0.20313	7.71021	0.00911	11.61
**'Delta MCF7'**	334.1605	65.12092	1.23268	1.00989	0.4468	0.19667	7.65494	0.01095	9.245
**'Delta MCF7'**	392.7738	70.00357	1.07866	0.99286	0.07335	0.20278	7.66268	0.01002	10.535
**'Delta MCF7'**	304.5765	62.86428	1.04958	1.03253	0.50151	0.18211	7.54988	0.00947	10.535
**'Delta MCF7'**	281.7414	61.83142	1.48424	1.07984	0.34333	0.19415	7.62551	0.01375	7.525
**'Delta MCF7'**	279.3839	59.99618	1.10054	1.02526	0.25673	0.17275	7.50706	0.00951	9.03
**'Delta MCF7'**	290.9402	61.12751	1.38978	1.02202	0.28987	0.19163	7.6167	0.00897	8.17
**'Delta MCF7'**	273.7907	58.64168	1.2062	0.9995	0.42815	0.2179	7.75626	0.00953	8.17
**'Delta MCF7'**	473.8987	79.47733	1.51392	1.0607	0.72634	0.20183	7.69711	0.00928	10.32
**'Delta MCF7'**	275.3161	60.5956	1.40039	1.06131	0.23885	0.22736	7.77735	0.01407	7.31
**'Delta MCF7'**	344.8847	67.46614	1.30286	1.05024	0.14	0.20762	7.69952	0.01518	9.03
**'Delta MCF7'**	246.934	56.18251	1.26315	1.01721	0.42429	0.23704	7.80713	0.01312	7.525
**'Delta MCF7'**	230.709	54.47025	1.2023	1.0234	0.12632	0.2067	7.67537	0.01344	7.31
**'Delta MCF7'**	216.4255	52.61738	1.16721	1.01798	0.31272	0.21956	7.72544	0.0114	7.095
**'Delta MCF7'**	265.9787	60.92326	1.45506	1.11048	0.22759	0.21242	7.71894	0.01859	6.45
**'Delta MCF7'**	297.8277	64.95064	1.67545	1.12718	0.23809	0.19813	7.65961	0.03113	7.31
**'Delta MCF7'**	352.9741	67.16041	1.25763	1.01689	0.4999	0.20897	7.74564	0.01229	9.46
**'Delta MCF7'**	221.464	56.11844	1.70263	1.13161	0.09036	0.20471	7.69308	0.02859	6.02
**'Delta MCF7'**	261.9109	58.73929	1.42459	1.04832	0.2842	0.21098	7.71071	0.01358	7.31
**'Delta MCF7'**	411.495	72.04865	1.22137	1.00387	0.21845	0.20021	7.73531	0.00902	10.105
**'Delta MCF7'**	346.179	67.16235	1.25884	1.03691	0.26533	0.2014	7.71082	0.01122	8.6
**'Delta MCF7'**	244.8076	56.09006	1.32315	1.02267	0.07635	0.22975	7.79397	0.01415	7.095
**'Delta MCF7'**	256.6874	57.71374	1.36208	1.03263	0.14887	0.20566	7.72621	0.01403	7.74
**'Delta MCF7'**	346.78	67.00561	1.37579	1.03029	0.28765	0.19794	7.66275	0.01094	9.03
**'Delta MCF7'**	307.6736	63.73073	1.33127	1.0505	0.14515	0.18422	7.58263	0.01276	6.88
**'Delta MCF7'**	345.763	65.75216	1.09348	0.99502	0.07884	0.17019	7.47938	0.01097	9.46
**'Delta MCF7'**	254.2837	57.19602	1.33858	1.02377	0.50678	0.23784	7.82091	0.01203	7.74

'Delta MCF7'	426.1483	75.20485	1.34955	1.05614	0.38828	0.22245	7.78227	0.01412	9.675
'Delta MCF7'	238.1974	56.20014	1.52683	1.05518	0.30793	0.22042	7.78084	0.01435	6.235
'Delta MCF7'	264.1297	60.17936	1.651	1.09111	0.17069	0.1899	7.48268	0.01142	3.655
'Delta MCF7'	441.1714	74.78689	1.0888	1.00887	0.49383	0.23276	7.72154	0.0115	11.825
'Delta MCF7'	411.3101	72.26365	1.15611	1.01032	0.38182	0.20878	7.7067	0.01001	10.535
'Delta MCF7'	407.7045	73.63492	1.2775	1.05831	0.84559	0.22159	7.80134	0.01902	11.18
'Delta MCF7'	344.5612	66.63624	1.21544	1.02552	0.20348	0.19984	7.66509	0.01219	9.245
'Delta MCF7'	397.0728	74.61747	1.33958	1.11584	0.49303	0.19816	7.68743	0.02409	10.105
'Delta MCF7'	383.5288	70.49635	1.38003	1.03116	0.35249	0.19548	7.67499	0.0086	9.245
'Delta MCF7'	355.7938	68.21305	1.37661	1.0407	0.20675	0.22509	7.74223	0.01219	8.815
'Delta MCF7'	206.9493	50.7185	1.17141	0.98914	0.1852	0.21422	7.71076	0.01126	6.665
'Delta MCF7'	351.726	66.66462	1.23034	1.00549	0.49593	0.22617	7.79715	0.0095	9.46
'Delta MCF7'	161.6488	46.3282	1.51654	1.05659	0.1234	0.20246	7.63275	0.01548	5.375
'Delta MCF7'	285.3932	65.57242	1.88292	1.19892	0.22928	0.20936	7.71821	0.01531	7.31
'Delta MCF7'	266.9032	58.3338	1.1802	1.01456	0.19606	0.19845	7.5737	0.01552	8.17
'Delta MCF7'	459.569	76.64148	1.18641	1.01711	0.83349	0.23585	7.86333	0.01065	11.18
'Delta MCF7'	243.1435	56.11328	1.24024	1.03053	0.53927	0.22133	7.75658	0.01553	7.955
'Delta MCF7'	328.6598	65.28647	1.20543	1.03202	0.37847	0.23981	7.75916	0.01387	9.46
'Delta MCF7'	292.6043	60.76287	1.19184	1.00412	0.3529	0.19702	7.64164	0.01248	9.03
'Delta MCF7'	359.3532	67.61191	1.19075	1.01231	0.11254	0.20832	7.70241	0.01056	9.03
'Delta MCF7'	171.0787	53.9994	1.87535	1.35635	0.07367	0.21483	7.68049	0.06611	4.73
'Delta MCF7'	221.7876	53.35182	1.20067	1.0213	0.30189	0.23776	7.74551	0.01741	8.385
'Delta MCF7'	410.1544	72.56938	1.30475	1.02176	0.76927	0.23843	7.86751	0.01004	9.675
'Delta MCF7'	297.689	61.35326	1.13764	1.00624	0.25402	0.21458	7.6655	0.01182	8.6
'Delta MCF7'	239.1682	56.32828	1.27449	1.0557	0.15144	0.20762	7.68425	0.01278	6.665
'Delta MCF7'	365.9171	69.07563	1.28501	1.03766	0.19365	0.18578	7.58842	0.01493	10.105
'Delta MCF7'	227.5657	53.75903	1.20658	1.01062	0.58271	0.21917	7.75357	0.01303	7.525
'Delta MCF7'	238.4286	55.12643	1.21044	1.01427	0.53267	0.2179	7.76256	0.01433	8.17
'Delta MCF7'	144.1296	44.80987	1.57291	1.10862	0.12169	0.19508	7.57924	0.01888	4.73

**'Delta MCF7'**	306.7029	64.75499	1.61392	1.08797	0.29399	0.21176	7.67875	0.01338	7.74
**'Delta MCF7'**	398.968	70.44647	1.11722	0.98985	0.27976	0.22124	7.75215	0.0085	10.32
**'Delta MCF7'**	259.3685	59.39719	1.56958	1.08244	0.34503	0.19416	7.64095	0.0194	7.74
**'Delta MCF7'**	310.5858	63.27536	1.23364	1.02584	0.15832	0.20564	7.7058	0.01249	9.245
**'Delta MCF7'**	424.8078	72.76331	1.06391	0.9918	0.16077	0.18027	7.55596	0.00948	11.61
**'Delta MCF7'**	485.871	78.35718	1.23724	1.0056	0.21305	0.16805	7.38886	0.00868	11.61
**'Delta MCF7'**	333.6983	64.52537	1.0808	0.99288	0.13142	0.19599	7.62517	0.01069	9.46
**'Delta MCF7'**	352.6505	66.20022	1.10588	0.98893	0.43792	0.18377	7.63081	0.00793	9.89
**'Delta MCF7'**	230.5703	55.05376	1.24658	1.04607	0.17761	0.22205	7.77958	0.02158	8.385
**'Delta MCF7'**	211.9416	55.22791	1.30984	1.14523	0.09193	0.22017	7.76149	0.03147	6.88
**'Delta MCF7'**	348.8139	66.76739	1.03101	1.01701	0.52533	0.21681	7.81116	0.0114	9.89
**'Delta MCF7'**	330.3239	65.57629	1.17589	1.03596	0.38975	0.20029	7.68556	0.01557	9.245
**'Delta MCF7'**	273.0973	68.15113	2.14521	1.35338	0.33328	0.23105	7.82896	0.09636	6.235
**'Delta MCF7'**	298.3362	62.64412	1.4081	1.04675	0.10307	0.21969	7.71448	0.01825	7.525
**'Delta MCF7'**	312.3423	63.53852	1.26142	1.02857	0.21787	0.20822	7.72682	0.01199	8.17
**'Delta MCF7'**	364.9926	68.15801	1.27583	1.01284	0.27549	0.21301	7.7436	0.01028	8.815
**'Delta MCF7'**	248.5981	58.11171	1.43287	1.08098	0.07493	0.18132	7.50298	0.01682	8.17
**'Delta MCF7'**	326.1174	65.80935	1.48995	1.0568	0.57	0.2114	7.73292	0.0108	8.385
**'Delta MCF7'**	303.9756	61.33004	1.06355	0.98469	0.78647	0.21699	7.75597	0.00889	9.89
**'Delta MCF7'**	379.2299	70.21341	1.36388	1.03449	0.22068	0.21228	7.74663	0.0093	9.245
**'Delta MCF7'**	373.3593	68.16876	1.05509	0.99045	0.14528	0.23351	7.76476	0.00944	10.105
**'Delta MCF7'**	271.2021	60.25246	1.4602	1.06524	0.03836	0.22288	7.77891	0.01824	6.665
**'Delta MCF7'**	382.1421	69.44715	1.17272	1.00432	0.78693	0.17586	7.60194	0.00958	10.32
**'Delta MCF7'**	259.3685	57.05197	1.11201	0.99865	0.38767	0.21546	7.75398	0.0118	8.385
**'Delta MCF7'**	339.1528	65.98909	1.27941	1.02174	0.60206	0.20229	7.62991	0.01145	9.03
**'Delta MCF7'**	298.3824	61.97719	1.32113	1.02443	0.45544	0.17056	7.45248	0.0127	7.525
**'Delta MCF7'**	322.1883	63.86919	1.21609	1.00754	0.22279	0.21641	7.7375	0.01051	8.6
**'Delta MCF7'**	358.2438	68.79828	1.47476	1.0514	0.25835	0.1993	7.72587	0.0111	8.6
**'Delta MCF7'**	309.6151	62.40246	1.14828	1.00086	0.38015	0.22737	7.82507	0.01122	9.245

**'Delta MCF7'**	297.3654	63.02123	1.53435	1.06285	0.10615	0.20669	7.66851	0.00985	8.385
**'Delta MCF7'**	300.6012	62.59424	1.37154	1.03721	0.33322	0.20199	7.71373	0.01095	7.525
**'Delta MCF7'**	326.2561	67.54827	1.58716	1.11291	0.18807	0.23151	7.78474	0.02108	8.385
**'Delta MCF7'**	338.4595	64.90764	1.08944	0.99055	0.54485	0.2255	7.81116	0.00853	9.675
**'Delta MCF7'**	289.1374	60.40554	1.16896	1.00424	0.76019	0.23313	7.82177	0.01232	8.385
**'Delta MCF7'**	341.0018	65.74872	1.29693	1.00881	0.14713	0.18586	7.5492	0.00847	9.46
**'Delta MCF7'**	260.2005	57.56969	1.17372	1.01361	0.32781	0.16613	7.39542	0.01107	9.03
**'Delta MCF7'**	375.8093	69.34395	1.23893	1.01822	0.37797	0.21175	7.6651	0.01167	9.675
**'Delta MCF7'**	382.743	71.0403	1.48929	1.04928	0.72373	0.21975	7.77525	0.01004	8.815
**'Delta MCF7'**	211.0171	57.35598	1.5405	1.24059	0.15858	0.20892	7.71157	0.05545	6.88
**'Delta MCF7'**	365.2237	68.06556	1.19294	1.00945	0.31566	0.21359	7.72885	0.01077	9.46
**'Delta MCF7'**	370.4009	72.48252	1.24445	1.12872	0.2144	0.18646	7.60084	0.03794	10.105
**'Delta MCF7'**	230.3392	56.29474	1.66224	1.09486	0.4797	0.23207	7.81031	0.01366	5.805
**'Delta MCF7'**	274.6227	58.98482	1.19586	1.00817	0.49434	0.22033	7.76534	0.01082	8.6
**'Delta MCF7'**	385.609	70.8855	1.3187	1.03695	0.13008	0.18136	7.59635	0.01685	9.89
**'Delta MCF7'**	436.6876	73.94925	1.13725	0.99652	0.12409	0.19558	7.58235	0.0085	11.61
**'Delta MCF7'**	398.5057	70.37165	1.09032	0.9889	0.09013	0.22273	7.67371	0.00897	10.965
**'Delta MCF7'**	341.9726	67.25673	1.27453	1.05262	0.23218	0.2096	7.7169	0.01518	8.815
**'Delta MCF7'**	308.3208	65.66874	1.57493	1.11302	0.27431	0.22433	7.73179	0.02585	7.955
**'Delta MCF7'**	315.6705	65.12264	1.50308	1.0691	0.40246	0.22845	7.81092	0.01684	9.245
**'Delta MCF7'**	403.7754	72.90005	1.43077	1.04738	0.28712	0.20821	7.71295	0.01143	9.03
**'Delta MCF7'**	323.8061	66.20194	1.45031	1.07708	0.1407	0.20421	7.62361	0.02055	7.095
**'Delta MCF7'**	327.4579	66.05273	1.15339	1.06027	0.27936	0.17613	7.49001	0.02249	9.46
**'Delta MCF7'**	313.3131	63.00704	1.18812	1.0083	0.0202	0.18155	7.55642	0.01411	9.89
**'Delta MCF7'**	314.6998	62.39343	1.03155	0.9844	0.33076	0.16214	7.3981	0.00975	10.105
**'Delta MCF7'**	242.3115	57.27987	1.44313	1.07751	0.15529	0.21132	7.70975	0.02601	4.73
**'Delta MCF7'**	185.1774	50.0004	1.44367	1.07436	0.19927	0.2037	7.637	0.01741	6.235
**'Delta MCF7'**	363.3747	70.17084	1.57449	1.07832	0.16857	0.21764	7.75294	0.00995	8.385
**'Delta MCF7'**	397.3039	71.03514	1.25061	1.01068	0.15501	0.20566	7.74519	0.01025	9.46

**'Delta MCF7'**	254.1451	60.09594	1.6599	1.13083	0.22609	0.21069	7.70234	0.01558	5.16
**'Delta MCF7'**	420.139	73.4655	1.37634	1.02227	0.07561	0.18227	7.46292	0.00775	9.46
**'Delta MCF7'**	270.0002	58.65759	1.26267	1.01409	0.07163	0.19456	7.64306	0.01184	7.525
**'Delta MCF7'**	453.1437	84.06866	1.73529	1.24114	0.25685	0.21931	7.77592	0.03967	9.245
**'Delta MCF7'**	301.803	64.76015	1.35334	1.10581	0.27337	0.22708	7.79086	0.02187	8.385
**'Delta MCF7'**	283.2206	62.11264	1.1823	1.08399	0.29088	0.21754	7.78045	0.02607	8.17
**'Delta MCF7'**	433.5905	78.16927	1.73367	1.12146	0.87216	0.21114	7.76148	0.01852	9.245
**'Delta MCF7'**	349.3686	66.26085	1.17489	1.00005	0.25624	0.21081	7.72401	0.0097	9.46
**'Delta MCF7'**	317.1035	65.48556	1.26215	1.07617	0.58314	0.21172	7.69932	0.02098	9.03
**'Delta MCF7'**	384.3147	70.09946	1.19801	1.0175	0.19616	0.17061	7.49137	0.00918	9.89
**'Delta MCF7'**	316.6875	63.16141	1.12072	1.00245	0.51374	0.21848	7.71946	0.01183	9.245
**'Delta MCF7'**	334.7152	64.90592	1.24074	1.00157	0.09128	0.19121	7.61965	0.00971	9.245
**'Delta MCF7'**	387.0419	71.53093	1.40463	1.05201	0.18271	0.21049	7.72994	0.01691	9.46
**'Delta MCF7'**	327.9202	69.45597	1.50451	1.17069	0.24868	0.19636	7.65915	0.02769	8.385
**'Delta MCF7'**	400.5396	73.39498	1.55891	1.07023	0.28984	0.21358	7.7289	0.01096	8.815
**'Delta MCF7'**	268.5673	58.97579	1.28254	1.03059	0.19625	0.20193	7.6039	0.01509	7.74
**'Delta MCF7'**	386.6259	70.54408	1.27129	1.02428	0.56844	0.21261	7.69369	0.01345	10.32
**'Delta MCF7'**	428.9218	74.99114	1.30035	1.04335	0.46584	0.22618	7.77189	0.01329	9.245
**'Delta MCF7'**	246.0095	58.0887	1.51675	1.09149	0.25065	0.21406	7.65279	0.02812	8.17
**'Delta MCF7'**	239.954	59.34258	1.81053	1.16787	0.11214	0.20309	7.66677	0.02571	6.02
**'Delta MCF7'**	306.7029	61.95784	1.20134	0.99601	0.15341	0.18388	7.50912	0.01	9.245
**'Delta MCF7'**	286.7799	61.5431	1.19059	1.05099	0.08411	0.19647	7.64707	0.01116	8.17
**'Delta MCF7'**	236.1635	59.52189	1.72011	1.1938	0.19964	0.2235	7.77713	0.03876	6.665
**'Delta MCF7'**	331.2021	64.8526	1.2335	1.01054	0.17009	0.19962	7.62655	0.01118	9.89
**'Delta MCF7'**	335.3624	66.98282	1.33312	1.06464	0.32749	0.2123	7.73955	0.0176	8.17
**'Delta MCF7'**	342.1112	67.37756	1.52545	1.05597	0.19188	0.22064	7.769	0.01096	8.17
**'Delta MCF7'**	380.9402	69.62345	1.13411	1.01262	0.1777	0.22883	7.73334	0.0127	9.675
**'Delta MCF7'**	228.7675	58.14933	1.21498	1.17621	0.15484	0.22034	7.78495	0.04552	7.74
**'Delta MCF7'**	341.2792	69.7703	1.47019	1.13507	0.26384	0.19952	7.67087	0.02586	9.03

**'Delta MCF7'**	346.5026	68.24315	1.4196	1.06955	0.29413	0.20682	7.73364	0.00951	8.385
**'Delta MCF7'**	188.0895	54.25697	2.05862	1.24548	0.24154	0.24226	7.84008	0.02023	5.16
**'Delta MCF7'**	300.0465	64.30306	1.60188	1.09664	0.19844	0.19372	7.63104	0.01652	6.88
**'Delta MCF7'**	296.5334	62.47341	1.46623	1.04739	0.16643	0.20792	7.69081	0.01217	7.74
**'Delta MCF7'**	322.7892	63.92982	1.14429	1.00758	0.04405	0.18577	7.55233	0.01133	9.89
**'Delta MCF7'**	254.6998	59.72657	1.79559	1.11454	0.33216	0.19886	7.63599	0.01308	6.45
**'Delta MCF7'**	253.4517	57.04681	1.27227	1.02178	0.24116	0.22393	7.80739	0.0142	7.955
**'Delta MCF7'**	276.0557	61.37863	1.4601	1.08599	0.35955	0.21477	7.74174	0.02784	8.17
**'Delta MCF7'**	305.9633	65.34667	1.43746	1.11063	0.3356	0.23922	7.83961	0.03415	8.17
**'Delta MCF7'**	256.5025	58.66318	1.39731	1.06765	0.27518	0.2063	7.71499	0.01961	7.525
**'Delta MCF7'**	278.9217	60.53712	1.48074	1.04557	0.56989	0.207	7.70351	0.01179	8.385
**'Delta MCF7'**	250.0773	55.83765	1.08028	0.99213	0.59023	0.21077	7.70421	0.01223	8.815
**'Delta MCF7'**	428.9218	74.04686	1.1312	1.01724	0.56518	0.2145	7.66334	0.01077	11.18
**'Delta MCF7'**	259.4609	59.05061	1.56627	1.06947	0.35341	0.20418	7.7292	0.01526	7.31
**'Delta MCF7'**	309.1528	63.23279	1.21996	1.0292	0.4094	0.22817	7.78557	0.01197	8.6
**'Delta MCF7'**	276.6104	66.22172	2.13993	1.2616	0.45046	0.20754	7.69457	0.02302	7.74
**'Delta MCF7'**	252.1112	57.57657	1.45932	1.04638	0.76785	0.22117	7.77689	0.01446	7.525
**'Delta MCF7'**	333.8832	68.73249	1.71201	1.12595	0.26855	0.20891	7.70485	0.01312	7.095
**'Delta MCF7'**	219.3839	54.50938	1.54544	1.07777	0.12452	0.21665	7.74865	0.01719	6.02
**'Delta MCF7'**	244.1605	55.40894	1.12824	1.00063	0.28575	0.20989	7.74277	0.01418	8.17
**'Delta MCF7'**	406.0866	72.91424	1.41405	1.04183	0.54065	0.20711	7.72631	0.00947	9.245
**'Delta MCF7'**	442.6506	74.75636	1.09704	1.00467	0.61854	0.18012	7.55284	0.00952	10.965
**'Delta MCF7'**	332.1266	68.99221	1.86612	1.14048	0.37358	0.22127	7.76599	0.01346	7.31
**'Delta MCF7'**	427.3964	73.34123	1.15486	1.00151	0.55148	0.20538	7.74235	0.01028	10.32
**'Delta MCF7'**	302.7275	63.74277	1.50223	1.06807	0.46081	0.22091	7.77422	0.01058	7.955
**'Delta MCF7'**	327.3655	65.41977	1.17731	1.04034	0.15644	0.21118	7.69025	0.01843	8.6
**'Delta MCF7'**	370.4472	69.65183	1.48838	1.04215	0.16503	0.17114	7.50231	0.00841	9.03
**'Delta MCF7'**	235.6551	55.64544	1.3255	1.04562	0.03803	0.22996	7.82423	0.02018	7.095
**'Delta MCF7'**	351.5874	67.23867	1.30348	1.02328	0.47425	0.19028	7.61472	0.01118	9.03

**'Delta MCF7'**	404.7461	73.25222	1.4558	1.05499	0.36245	0.20545	7.67429	0.0114	9.675
**'Delta MCF7'**	388.1513	70.40906	1.25199	1.01636	0.10823	0.1934	7.63857	0.01072	9.675
**'Delta MCF7'**	348.7676	68.49255	1.41666	1.07039	0.41006	0.2177	7.71247	0.01463	9.03
**'Delta MCF7'**	245.1312	56.31753	1.38832	1.02962	0.56689	0.19777	7.66908	0.01358	7.31
**'Delta MCF7'**	421.2022	75.63657	1.53648	1.08084	0.37621	0.20521	7.74983	0.01268	7.74
**'Delta MCF7'**	214.4378	54.74975	1.56822	1.11238	0.38536	0.24023	7.85107	0.01445	5.59
**'Delta MCF7'**	292.6505	61.50247	1.37796	1.02855	0.42535	0.2057	7.72184	0.01279	7.74
**'Delta MCF7'**	318.8601	64.21448	1.43127	1.0291	0.0601	0.18785	7.59207	0.00891	7.74
**'Delta MCF7'**	349.7384	68.12791	1.47995	1.05608	0.14552	0.22512	7.70374	0.01214	8.385
**'Delta MCF7'**	386.6721	69.55379	1.18173	0.99561	0.05663	0.20058	7.61804	0.00842	10.105
**'Delta MCF7'**	447.042	78.05876	1.56252	1.08464	0.61119	0.19517	7.63309	0.01236	10.965
**'Delta MCF7'**	306.0557	62.96963	1.29694	1.03098	0.27711	0.21578	7.67854	0.01415	8.6
**'Delta MCF7'**	323.8986	64.50043	1.2419	1.02213	0.02276	0.21702	7.75674	0.01338	8.385
**'Delta MCF7'**	383.2053	69.64452	1.20675	1.00724	0.05399	0.18033	7.50991	0.01086	10.105
**'Delta MCF7'**	445.7939	77.5677	1.51341	1.07403	0.14625	0.19703	7.65001	0.01189	9.46
**'Delta MCF7'**	370.2623	68.2238	1.12695	1.00035	0.20615	0.21649	7.72191	0.01136	9.675
**'Delta MCF7'**	391.8493	70.64556	1.23809	1.01354	0.35089	0.20788	7.72305	0.01189	9.675
**'Delta MCF7'**	262.0958	59.8474	1.666	1.08748	0.17865	0.20066	7.70314	0.01391	6.235
**'Delta MCF7'**	323.991	64.23211	1.30789	1.01335	0.26902	0.1999	7.72186	0.00989	8.815
**'Delta MCF7'**	304.0218	61.90452	1.16576	1.00307	0.11282	0.19087	7.55387	0.01202	9.03
**'Delta MCF7'**	257.011	57.78297	1.22901	1.0338	0.19335	0.22962	7.73035	0.01819	7.74
**'Delta MCF7'**	270.1851	58.74101	1.17084	1.01628	0.25251	0.24056	7.76788	0.01234	8.17
**'Delta MCF7'**	259.877	56.96124	1.07062	0.99353	0.32623	0.20039	7.6904	0.0123	8.6
**'Delta MCF7'**	293.3439	61.1417	1.24849	1.01412	0.27792	0.21297	7.76701	0.00998	8.385
**'Delta MCF7'**	195.8091	49.86495	1.26255	1.01053	0.71704	0.22847	7.77999	0.01305	7.31
**'Delta MCF7'**	376.0404	68.86751	1.17654	1.00365	0.23069	0.19146	7.60793	0.01058	9.675
**'Delta MCF7'**	337.2114	65.44643	1.23975	1.01079	0.4759	0.18677	7.58548	0.01044	9.245
**'Delta MCF7'**	284.0526	59.81515	1.18358	1.00234	0.35672	0.1978	7.66498	0.0119	9.245
**'Delta MCF7'**	282.2036	59.47201	1.20669	0.99736	0.20943	0.2039	7.6295	0.01037	8.385

**'Delta MCF7'**	353.3439	67.39175	1.28476	1.02284	0.22874	0.1934	7.6181	0.01138	9.245
**'Delta MCF7'**	306.2869	63.78577	1.28477	1.05708	0.05704	0.18763	7.66008	0.02587	9.245
**'Delta MCF7'**	461.1868	77.23703	1.32068	1.02935	0.06441	0.18755	7.58413	0.01208	10.535
**'Delta MCF7'**	339.7075	66.95272	1.52074	1.05008	0.21726	0.21631	7.69204	0.00957	8.6
**'Delta MCF7'**	337.8585	65.41418	1.03075	1.00786	0.27381	0.19674	7.62936	0.01216	9.675
**'Delta MCF7'**	329.8616	65.31485	1.26678	1.02916	0.72473	0.22446	7.79269	0.01204	8.815
**'Delta MCF7'**	348.9525	66.07767	1.20289	0.99571	0.0164	0.15896	7.3121	0.00906	9.245
**'Delta MCF7'**	281.464	59.81687	1.15843	1.01161	0.551	0.20473	7.69747	0.01345	8.6
**'Delta MCF7'**	391.2484	71.1822	1.21242	1.03058	0.29427	0.20778	7.69699	0.01248	9.46
**'Delta MCF7'**	319.5072	69.26225	1.44385	1.19482	0.43005	0.21631	7.76072	0.03933	9.89
**'Delta MCF7'**	487.6738	78.65216	1.10754	1.00944	0.27735	0.22383	7.76626	0.01162	11.825
**'Delta MCF7'**	453.3748	80.1907	1.57604	1.12871	0.50227	0.2011	7.66469	0.03417	10.105
**'Delta MCF7'**	240.786	55.34143	1.13915	1.01218	0.45682	0.19647	7.65801	0.01457	7.955
**'Delta MCF7'**	312.1112	65.83773	1.61745	1.10517	0.20624	0.22367	7.77235	0.02117	8.17
**'Delta MCF7'**	319.461	64.98418	1.51294	1.05193	0.16307	0.20901	7.67266	0.01059	7.525
**'Delta MCF7'**	398.968	72.10541	1.40191	1.03702	0.15417	0.21668	7.64747	0.01077	8.385
**'Delta MCF7'**	316.8724	64.13579	1.33271	1.03302	0.31489	0.20016	7.63381	0.01324	8.385
**'Delta MCF7'**	303.3285	61.88302	1.0489	1.00466	0.34707	0.21179	7.66571	0.01338	9.46
**'Delta MCF7'**	263.8523	61.82626	1.81743	1.15286	0.40912	0.2235	7.78355	0.0266	7.525
**'Delta MCF7'**	414.4996	72.27784	1.20631	1.00294	0.36046	0.16009	7.45831	0.00939	9.89
**'Delta MCF7'**	322.3732	64.28715	1.29455	1.02019	0.40751	0.1951	7.69867	0.00923	9.03
**'Delta MCF7'**	314.0989	66.76395	1.32994	1.1293	0.18018	0.21121	7.73311	0.02929	8.17
**'Delta MCF7'**	224.4224	55.24339	1.52802	1.08214	0.10897	0.23461	7.82744	0.01959	6.45
**'Delta MCF7'**	170.7089	48.50099	1.55494	1.09657	0.1728	0.22053	7.7426	0.0186	4.73
**'Delta MCF7'**	276.8415	61.42292	1.63333	1.08448	0.06417	0.20051	7.67938	0.01351	6.88
**'Delta MCF7'**	304.2067	62.71335	1.24657	1.02882	0.43182	0.20912	7.70522	0.01186	7.955
**'Delta MCF7'**	295.2853	61.46506	1.22402	1.01813	0.57807	0.21515	7.75332	0.01481	9.46
**'Delta MCF7'**	317.8431	64.14654	1.33046	1.03021	0.0596	0.2315	7.81553	0.01178	8.17
**'Delta MCF7'**	442.0497	75.69892	1.39541	1.03157	0.50031	0.21633	7.76683	0.00994	9.89

**'Delta MCF7'**	345.6243	65.55135	1.09818	0.98935	0.21002	0.1923	7.64407	0.00941	9.89
**'Delta MCF7'**	225.1158	55.9301	1.69098	1.1058	0.22819	0.22375	7.61073	0.01596	7.095
**'Delta MCF7'**	306.0095	65.12823	1.68218	1.10305	0.32962	0.19822	7.6509	0.01532	7.095
**'Delta MCF7'**	427.6737	74.93094	1.34243	1.04472	0.12692	0.2093	7.72131	0.01627	10.965
**'Delta MCF7'**	376.7338	70.14246	1.11441	1.03924	0.11521	0.18429	7.48724	0.02079	10.535
**'Delta MCF7'**	323.991	63.73245	1.13177	0.99765	0.42945	0.2286	7.82426	0.01184	9.245
**'Delta MCF7'**	266.672	63.24999	1.78686	1.19381	0.55915	0.19432	7.63043	0.05068	7.095
**'Delta MCF7'**	390.1852	71.36022	1.23792	1.03856	0.68478	0.22285	7.77853	0.01252	9.675
**'Delta MCF7'**	276.7953	60.09981	1.41085	1.03843	0.48541	0.20377	7.72435	0.01237	7.095
**'Delta MCF7'**	388.5674	75.31837	1.42371	1.16178	0.28467	0.20075	7.70483	0.05847	9.89
**'Delta MCF7'**	367.6737	67.77918	1.13092	0.99431	0.59651	0.22048	7.8018	0.00971	9.675
**'Delta MCF7'**	256.0865	57.50906	1.37017	1.02772	0.36103	0.21613	7.7531	0.01371	7.525
**'Delta MCF7'**	467.3348	76.9313	1.07509	1.00779	0.43117	0.21848	7.76092	0.0096	12.04
**'Delta MCF7'**	433.8216	74.76539	1.32696	1.02537	0.81407	0.21405	7.76851	0.01002	10.535
**'Delta MCF7'**	278.8754	59.11812	1.11288	0.99729	0.07281	0.21976	7.75877	0.01147	8.385
**'Delta MCF7'**	238.937	55.17803	1.23972	1.014	0.1306	0.24123	7.84185	0.01336	7.525
**'Delta MCF7'**	385.5627	69.70859	1.19696	1.00292	0.12499	0.21073	7.67543	0.00903	10.32
**'Delta MCF7'**	384.8231	70.33252	1.22146	1.02292	0.29936	0.21213	7.74358	0.01152	9.89
**'Delta MCF7'**	442.327	75.89285	1.40643	1.03621	0.16917	0.19599	7.68177	0.01034	9.675
**'Delta MCF7'**	407.5196	71.12931	1.06133	0.98796	0.17884	0.20837	7.67731	0.00777	10.535
**'Delta MCF7'**	263.2976	59.37956	1.51173	1.06566	0.29114	0.20875	7.72742	0.01334	6.45
**'Delta MCF7'**	422.8663	72.47521	1.07327	0.98848	0.18573	0.21735	7.74189	0.00856	11.395
**'Delta MCF7'**	261.5873	56.93114	1.08742	0.98599	0.17374	0.21552	7.75126	0.00945	8.385
**'Delta MCF7'**	264.6381	57.55507	1.20144	0.9961	0.34561	0.21063	7.74275	0.01123	8.17
**'Delta MCF7'**	394.2993	72.72074	1.20987	1.06729	0.11217	0.21555	7.75393	0.02213	9.89
**'Delta MCF7'**	417.7353	72.42361	1.10252	0.99919	0.69123	0.22199	7.767	0.01029	11.18
**'Delta MCF7'**	242.1728	59.62294	1.79125	1.16813	0.17113	0.20984	7.69497	0.04048	6.88
**'Delta MCF7'**	444.9156	75.10509	1.2389	1.00891	0.08105	0.18322	7.51482	0.00896	10.75
**'Delta MCF7'**	295.5627	61.70715	1.22774	1.02521	0.40079	0.20674	7.69083	0.01479	9.245

**'Delta MCF7'**	367.8123	70.54623	1.60471	1.07674	0.25658	0.19682	7.65814	0.0118	7.74
**'Delta MCF7'**	224.3299	54.61043	1.08755	1.05792	0.42711	0.20816	7.71614	0.03346	8.17
**'Delta MCF7'**	343.2206	66.46166	1.26934	1.02414	0.3285	0.19584	7.68971	0.0129	8.815
**'Delta MCF7'**	388.6598	70.70232	1.35174	1.0235	0.2359	0.20685	7.63376	0.00954	9.03
**'Delta MCF7'**	333.0511	65.90352	1.40107	1.03776	0.33366	0.21143	7.73108	0.01044	8.17
**'Delta MCF7'**	318.444	63.80168	1.31443	1.01723	0.1213	0.22781	7.79006	0.01148	8.17
**'Delta MCF7'**	433.1745	74.5147	1.20381	1.02003	0.09402	0.21154	7.72667	0.01202	9.675
**'Delta MCF7'**	281.5103	61.27844	1.55971	1.06148	0.37101	0.22007	7.75391	0.0112	7.31
**'Delta MCF7'**	259.0911	59.78849	1.26162	1.09793	0.03804	0.21196	7.74517	0.02691	7.095
**'Delta MCF7'**	313.8678	64.52365	1.45813	1.05556	0.14138	0.20476	7.64974	0.01236	8.17
**'Delta MCF7'**	384.9618	69.66043	1.16182	1.0031	0.2062	0.20539	7.70944	0.0101	9.89
**'Delta MCF7'**	401.3255	72.33675	1.28565	1.03756	0.05974	0.20788	7.72167	0.01665	10.105
**'Delta MCF7'**	268.9833	60.07143	1.20208	1.06758	0.37248	0.1965	7.65814	0.01955	8.385
**'Delta MCF7'**	319.9232	65.73453	1.55483	1.07481	0.31887	0.1915	7.61793	0.01424	7.955
**'Delta MCF7'**	231.3561	55.64372	1.50212	1.06498	0.34987	0.22639	7.77975	0.01651	6.88
**'Delta MCF7'**	190.7244	49.36529	1.07104	1.01678	0.50785	0.19025	7.60204	0.01809	9.03
**'Delta MCF7'**	263.2514	59.9678	1.59052	1.08707	0.38282	0.20224	7.6581	0.01607	7.525
**'Delta MCF7'**	402.6198	71.4402	1.12466	1.00874	0.21119	0.2056	7.72141	0.00854	10.105
**'Delta MCF7'**	196.3638	50.34483	1.17626	1.02716	0.42449	0.21829	7.7015	0.01507	6.88
**'Delta BJ'**	260.5241	61.31435	1.71977	1.14833	0.24377	0.1404	7.36585	0.02373	3.44
**'Delta BJ'**	203.6674	53.72355	1.4518	1.12771	0.31932	0.12885	7.19602	0.0325	5.805
**'Delta BJ'**	345.347	67.82734	1.5021	1.06009	0.20673	0.13409	7.27351	0.01399	8.385
**'Delta BJ'**	241.803	56.41385	1.30475	1.04737	0.03964	0.14987	7.3843	0.01765	2.58
**'Delta BJ'**	196.1789	50.82514	1.51834	1.04784	0.08533	0.13094	7.14915	0.01348	1.505
**'Delta BJ'**	241.0634	56.58628	1.25354	1.05702	0.14854	0.15641	7.36657	0.0245	5.805
**'Delta BJ'**	187.8122	50.4605	1.6676	1.07887	0.09956	0.15379	7.3733	0.01264	2.365
**'Delta BJ'**	195.9016	52.0573	1.66085	1.10082	0.09431	0.15664	7.42192	0.01944	5.805
**'Delta BJ'**	159.8461	47.08049	1.63493	1.10349	0.33594	0.15803	7.35255	0.01538	5.59
**'Delta BJ'**	174.1758	49.38679	1.50681	1.11436	0.19644	0.14916	7.205	0.0251	1.72

**'Delta BJ'**	243.0973	56.11844	1.38172	1.03091	0.19951	0.11731	7.28103	0.01369	2.15
**'Delta BJ'**	218.4131	52.99105	1.31506	1.0231	0.10363	0.14208	7.30048	0.01439	1.935
**'Delta BJ'**	451.8032	78.59884	1.58806	1.08811	0.34865	0.15216	7.49088	0.01422	10.535
**'Delta BJ'**	222.1111	55.77896	1.72979	1.11471	0.24799	0.1146	7.06679	0.01678	1.72
**'Delta BJ'**	192.8045	51.19322	1.60348	1.08168	0.12479	0.12534	7.12519	0.01465	5.16
**'Delta BJ'**	250.031	58.35379	1.32156	1.08376	0.21109	0.12821	7.21935	0.01762	8.6
**'Delta BJ'**	219.6612	55.46054	1.4528	1.11431	0.26853	0.15696	7.44249	0.03747	7.74
**'Delta BJ'**	195.578	52.61953	1.26346	1.12659	0.15547	0.16336	7.40268	0.03819	7.31
**'Delta BJ'**	253.2668	57.85736	1.48255	1.05179	0.24449	0.14942	7.50365	0.01333	7.31
**'Delta BJ'**	183.6519	48.98474	1.43925	1.03972	0.0349	0.13555	7.18913	0.01634	4.945
**'Delta BJ'**	215.4085	55.01248	1.43986	1.11802	0.21913	0.13135	7.31306	0.03199	7.525
**'Delta BJ'**	543.606	84.0134	1.18561	1.03324	0.25758	0.1334	7.30043	0.01738	6.665
**'Delta BJ'**	306.518	63.88897	1.47437	1.05971	0.16586	0.12378	7.24369	0.01632	8.17
**'Delta BJ'**	222.9432	53.8317	1.33116	1.03436	0.25578	0.16185	7.4248	0.01632	7.31
**'Delta BJ'**	165.6242	46.96288	1.52324	1.05968	0.19178	0.13613	7.26854	0.01836	2.795
**'Delta BJ'**	375.763	71.21466	1.19415	1.07403	0.14814	0.14892	7.31705	0.02786	5.16
**'Delta BJ'**	108.675	37.13759	1.15193	1.00992	0.27637	0.14674	7.12296	0.01632	3.01
**'Delta BJ'**	590.247	89.08439	1.48619	1.06994	0.10155	0.13207	7.44673	0.01428	4.73
**'Delta BJ'**	248.9679	57.4394	1.4893	1.05455	0.01772	0.15591	7.42402	0.01409	6.665
**'Delta BJ'**	261.6335	60.52658	1.72577	1.11427	0.15166	0.13691	7.20283	0.01906	7.74
**'Delta BJ'**	164.4223	47.55198	1.64116	1.09438	0.21355	0.14529	7.32081	0.01984	5.375
**'Delta BJ'**	146.3021	44.26764	1.45996	1.06589	0.08074	0.13472	7.17778	0.02073	4.73
**'Delta BJ'**	132.6658	41.13982	1.24043	1.01521	0.18544	0.14242	7.16202	0.0188	5.805
**'Delta BJ'**	180.6011	49.36357	1.46136	1.0737	0.3194	0.19598	7.65607	0.02178	6.665
**'Delta BJ'**	232.4655	57.97303	1.70962	1.15049	0.34112	0.16491	7.42124	0.02971	4.085
**'Delta BJ'**	257.4733	59.45094	1.50583	1.09238	0.21035	0.15038	7.44582	0.02246	7.525
**'Delta BJ'**	195.2544	53.76957	1.7816	1.17832	0.12165	0.15773	7.38772	0.022	1.72
**'Delta BJ'**	387.9202	71.1134	1.37134	1.03741	0.159	0.12771	7.2816	0.01119	8.6
**'Delta BJ'**	191.4177	51.75673	1.68669	1.11363	0.22736	0.17911	7.54621	0.02656	6.02

**'Delta BJ'**	395.7785	74.31905	1.7206	1.11055	0.09454	0.14296	7.43013	0.01439	9.03
**'Delta BJ'**	205.655	56.82665	2.1967	1.24955	0.2912	0.17958	7.57928	0.02562	5.59
**'Delta BJ'**	186.6103	49.67833	1.28433	1.05242	0.24925	0.17836	7.58523	0.01561	6.02
**'Delta BJ'**	229.5071	55.79164	1.64971	1.07928	0.05319	0.14691	7.41043	0.01174	6.235
**'Delta BJ'**	177.4578	48.86241	1.42564	1.07064	0.14139	0.15389	7.36274	0.01766	6.02
**'Delta BJ'**	145.4239	46.45656	1.97401	1.181	0.19589	0.151	7.32667	0.02268	5.16
**'Delta BJ'**	192.4347	51.51852	1.50537	1.09757	0.30127	0.15418	7.36723	0.01978	10.535
**'Delta BJ'**	160.3545	45.14054	1.13228	1.01121	0.40217	0.18649	7.55472	0.02116	6.88
**'Delta BJ'**	494.5151	85.16172	1.88399	1.16708	0.45757	0.14559	7.43883	0.01483	7.74
**'Delta BJ'**	284.1913	63.62344	1.74099	1.13348	0.24689	0.15823	7.43351	0.01805	6.45
**'Delta BJ'**	176.1173	50.16724	1.62359	1.13718	0.07685	0.16127	7.41273	0.02781	4.085
**'Delta BJ'**	183.6982	51.92573	1.57371	1.16802	0.27856	0.1518	7.30964	0.03426	6.235
**'Delta BJ'**	164.2374	46.32132	1.47627	1.03963	0.10101	0.12406	7.07848	0.0136	1.935
**'Delta BJ'**	259.7845	58.55976	1.33199	1.05045	0.1906	0.13358	7.13727	0.02431	7.525
**'Delta BJ'**	294.7306	65.55285	1.78825	1.16024	0.2441	0.18999	7.63989	0.01938	6.88
**'Delta BJ'**	412.9279	73.3236	1.3407	1.0361	0.30874	0.1411	7.3498	0.01249	3.01
**'Delta BJ'**	407.9356	74.4846	1.21516	1.08226	0.38313	0.15581	7.40835	0.02194	12.04
**'Delta BJ'**	256.7337	58.52257	1.44012	1.06158	0.36302	0.16044	7.49822	0.01717	7.74
**'Delta BJ'**	194.9308	52.48236	1.58102	1.12444	0.1072	0.14365	7.37455	0.02834	6.02
**'Delta BJ'**	134.561	41.90801	1.07682	1.03864	0.10962	0.15796	7.35377	0.03031	5.59
**'Delta BJ'**	153.8368	43.84624	1.06146	0.99448	0.12573	0.16379	7.44884	0.01509	6.45
**'Delta BJ'**	163.1743	49.9774	1.92897	1.21811	0.1009	0.17614	7.50279	0.03394	6.02
**'Delta BJ'**	271.4794	60.11207	1.46634	1.0592	0.10931	0.1416	7.31254	0.01641	4.3
**'Delta BJ'**	261.726	58.28263	1.32712	1.03281	0.06434	0.13766	7.34653	0.01496	7.955
**'Delta BJ'**	284.7922	61.44227	1.40322	1.05486	0.32236	0.14573	7.38903	0.01817	9.03
**'Delta BJ'**	328.5673	65.71841	1.08417	1.04602	0.65433	0.14105	7.22503	0.02175	5.375
**'Delta BJ'**	256.179	58.50816	1.49746	1.06336	0.1206	0.14595	7.33596	0.01546	3.225
**'Delta BJ'**	237.5041	55.81443	1.33827	1.04379	0.10309	0.14369	7.26768	0.01872	2.58
**'Delta BJ'**	358.706	71.95126	1.87102	1.14849	0.19462	0.16923	7.55264	0.01648	5.805

**'Delta BJ'**	269.3993	62.83418	1.93552	1.16623	0.27212	0.14876	7.33765	0.0177	3.655
**'Delta BJ'**	357.6428	68.28271	1.16049	1.03744	0.20877	0.15613	7.39877	0.02446	9.89
**'Delta BJ'**	223.5441	56.19498	1.19528	1.12414	0.03725	0.15016	7.42371	0.03646	7.525
**'Delta BJ'**	227.8893	55.81658	1.65502	1.08791	0.02899	0.16154	7.45789	0.0138	6.02
**'Delta BJ'**	176.672	48.67213	1.42392	1.06705	0.12501	0.15515	7.36993	0.01899	5.59
**'Delta BJ'**	144.5456	46.29832	1.9775	1.18009	0.1877	0.1562	7.36228	0.02067	5.16
**'Delta BJ'**	387.0882	71.07599	1.36555	1.03855	0.14864	0.12808	7.25909	0.0125	8.6
**'Delta BJ'**	188.9216	51.45101	1.67598	1.11506	0.24944	0.17805	7.53559	0.02551	6.235
**'Delta BJ'**	449.0759	78.647	1.63352	1.09606	0.12226	0.15765	7.47807	0.013	9.03
**'Delta BJ'**	214.8076	55.3324	1.79447	1.13423	0.19394	0.13208	7.20251	0.01941	3.225
**'Delta BJ'**	226.5487	56.02599	1.46633	1.10257	0.16903	0.19626	7.66149	0.03808	7.095
**'Delta BJ'**	196.0402	50.57789	1.36724	1.0384	0.16179	0.14443	7.32956	0.01851	8.385
**'Delta BJ'**	472.0497	78.89038	1.49136	1.04918	0.21608	0.13343	7.33405	0.00922	10.75
**'Delta BJ'**	206.5333	52.19598	1.42111	1.04972	0.06435	0.13049	7.1604	0.01694	1.72
**'Delta BJ'**	347.5658	69.88102	1.27847	1.11808	0.26392	0.13703	7.31517	0.02528	4.3
**'Delta BJ'**	225.1158	55.35949	1.58407	1.08335	0.02507	0.14591	7.33577	0.01457	4.515
**'Delta BJ'**	177.1342	49.5575	1.57051	1.10333	0.12243	0.16647	7.48217	0.02518	7.31
**'Delta BJ'**	216.1019	52.71177	1.29735	1.02317	0.10934	0.14337	7.24924	0.01454	8.17
**'Delta BJ'**	164.5148	47.90028	1.7418	1.10984	0.17428	0.14164	7.25737	0.01712	1.72
**'Delta BJ'**	344.0989	67.77574	1.32711	1.06232	0.28516	0.1547	7.41688	0.01742	3.655
**'Delta BJ'**	219.3839	55.30273	1.65691	1.10938	0.06737	0.13131	7.2415	0.01861	6.88
**'Delta BJ'**	316.1328	63.58474	1.14431	1.01772	0.09022	0.14807	7.41442	0.01384	11.18
**'Delta BJ'**	216.3792	54.46294	1.63384	1.09088	0.18383	0.16408	7.50998	0.01639	3.655
**'Delta BJ'**	190.031	50.89802	1.4964	1.08484	0.03601	0.15784	7.40301	0.02026	6.45
**'Delta BJ'**	261.2175	60.56722	1.55351	1.11754	0.11113	0.15218	7.44223	0.02011	4.3
**'Delta BJ'**	342.3886	67.18578	1.26624	1.04912	0.1802	0.16058	7.45624	0.01803	4.515
**'Delta BJ'**	452.0343	79.9327	1.73085	1.12478	0.00602	0.14307	7.42002	0.01511	5.16
**'Delta BJ'**	151.9878	51.2861	2.67	1.37715	0.23211	0.17473	7.46952	0.02085	4.3
**'Delta BJ'**	294.2684	67.61384	1.97099	1.23628	0.1291	0.1482	7.35723	0.03706	6.88

**'Delta BJ'**	209.6304	52.8986	1.46682	1.06224	0.11059	0.15646	7.43401	0.02031	6.45
**'Delta BJ'**	187.8122	49.91999	1.38804	1.05588	0.16865	0.12792	7.14149	0.02049	5.59
**'Delta BJ'**	178.3361	49.86323	1.70476	1.10946	0.26614	0.16698	7.50459	0.02255	5.805
**'Delta BJ'**	310.3547	67.35047	1.85301	1.16309	0.26443	0.15453	7.46286	0.02	9.03
**'Delta BJ'**	214.6689	54.9841	1.38235	1.12072	0.24166	0.15615	7.51337	0.03391	9.03
**'Delta BJ'**	358.4287	70.38047	1.17692	1.09974	0.42468	0.16821	7.53958	0.03232	10.105
**'Delta BJ'**	197.3345	51.55614	1.46283	1.07188	0.12577	0.14191	7.26484	0.01591	6.45
**'Delta BJ'**	217.1651	53.2985	1.25477	1.04095	0.11775	0.12046	7.11568	0.02894	4.515
**'Delta BJ'**	289.5534	62.96791	1.51443	1.08968	0.18023	0.12132	7.07187	0.01509	3.01
**'Delta BJ'**	222.5734	55.06773	1.25528	1.0842	0.34633	0.1058	7.07086	0.02884	8.17
**'Delta BJ'**	320.9864	67.66566	1.84542	1.13512	0.11458	0.13742	7.26873	0.01782	3.01
**'Delta BJ'**	229.5071	55.1991	1.50018	1.05647	0.10749	0.15218	7.45482	0.01664	6.88
**'Delta BJ'**	200.0156	52.04849	1.46751	1.07781	0.15885	0.17194	7.60034	0.02479	6.45
**'Delta BJ'**	373.7291	70.88937	1.34297	1.07003	0.10795	0.18947	7.67677	0.02308	9.46
**'Delta BJ'**	519.0605	80.78797	1.06263	1.00061	0.1655	0.11343	7.27164	0.01057	9.89
**'Delta BJ'**	210.8322	52.68145	1.48547	1.04754	0.16574	0.13768	7.42026	0.01384	3.655
**'Delta BJ'**	261.2637	58.28435	1.38909	1.0347	0.09805	0.13468	7.23895	0.01413	8.17
**'Delta BJ'**	368.0435	70.49807	1.53083	1.0746	0.24571	0.13144	7.2978	0.02018	10.965
**'Delta BJ'**	483.7909	81.74085	1.66171	1.09903	0.11399	0.16228	7.48949	0.01487	9.675
**'Delta BJ'**	332.2191	66.08842	1.21216	1.0462	0.13818	0.13827	7.33079	0.01615	10.75
**'Delta BJ'**	301.387	61.25909	1.09827	0.99085	0.02759	0.13075	7.23835	0.01062	6.02
**'Delta BJ'**	307.4425	62.33129	1.11028	1.00563	0.27499	0.15797	7.48706	0.01408	11.825
**'Delta BJ'**	268.5673	59.75495	1.49644	1.058	0.19734	0.15262	7.36926	0.01341	3.225
**'Delta BJ'**	238.7521	56.46524	1.3157	1.06269	0.15965	0.14357	7.25492	0.02437	2.58
**'Delta BJ'**	189.1989	50.86986	1.59591	1.08841	0.12157	0.15561	7.42775	0.0187	4.3
**'Delta BJ'**	172.4655	46.9044	1.29708	1.01511	0.24669	0.14692	7.32697	0.01712	7.095
**'Delta BJ'**	275.0388	59.0863	1.30929	1.01011	0.11433	0.12625	7.22616	0.01048	8.17
**'Delta BJ'**	284.6073	63.15431	1.74278	1.11519	0.47721	0.12879	7.25466	0.01409	5.805
**'Delta BJ'**	276.3331	60.19742	1.44973	1.04355	0.15326	0.14538	7.30945	0.01272	3.225

**'Delta BJ'**	352.2345	67.9271	1.33306	1.04242	0.19913	0.13398	7.30259	0.01461	9.89
**'Delta BJ'**	246.2868	57.92874	1.59708	1.08427	0.24984	0.15867	7.39264	0.01897	7.74
**'Delta BJ'**	236.3484	56.07415	1.46671	1.05868	0.02023	0.13203	7.23553	0.01578	7.74
**'Delta BJ'**	320.8477	64.66748	1.24804	1.0372	0.23012	0.1413	7.39494	0.01699	3.655
**'Delta BJ'**	194.561	51.17	1.6348	1.07094	0.08234	0.14483	7.38418	0.01382	6.02
**'Delta BJ'**	204.1758	51.91863	1.24974	1.05059	0.1194	0.11612	7.37114	0.0288	4.3
**'Delta BJ'**	204.0834	51.97195	1.45043	1.05322	0.22079	0.14347	7.30533	0.01932	7.095
**'Delta BJ'**	172.1881	47.40406	1.36313	1.03853	0.22777	0.15136	7.35308	0.01819	3.44
**'Delta BJ'**	255.0696	58.6649	1.41612	1.07371	0.1213	0.14959	7.38577	0.01797	8.385
**'Delta BJ'**	200.108	51.30373	1.11657	1.0467	0.41122	0.16036	7.32212	0.01926	0
**'Delta BJ'**	231.4948	55.94967	1.514	1.07608	0.05784	0.13989	7.23839	0.02568	4.085
**'Delta BJ'**	214.7151	56.3199	1.8476	1.17558	0.22176	0.18704	7.60178	0.02313	6.235
**'Delta BJ'**	209.8615	54.4079	1.76733	1.12249	0.27503	0.16527	7.47699	0.01774	5.375
**'Delta BJ'**	313.7291	67.662	1.58963	1.16125	0.05261	0.13857	7.3035	0.03278	2.58
**'Delta BJ'**	247.3962	57.78104	1.55743	1.07391	0.09677	0.16477	7.49419	0.01545	6.235
**'Delta BJ'**	225.994	54.84349	1.33889	1.05912	0.33884	0.1718	7.53507	0.01768	6.665
**'Delta BJ'**	297.4579	65.51609	1.60025	1.14831	0.1666	0.1634	7.44676	0.03639	8.385
**'Delta BJ'**	404.099	73.40336	1.40147	1.06105	0.4035	0.16951	7.56786	0.01465	4.945
**'Delta BJ'**	268.6135	59.65175	1.48085	1.05417	0.27181	0.15144	7.35806	0.01308	6.88
**'Delta BJ'**	287.6582	66.60034	1.77008	1.22706	0.05464	0.15355	7.36062	0.03549	3.01
**'Delta BJ'**	127.9508	41.01727	1.32876	1.04636	0.18871	0.17211	7.21747	0.02191	6.02
**'Delta BJ'**	155.4085	46.764	1.4523	1.1198	0.12317	0.16394	7.41714	0.0286	6.88
**'Delta BJ'**	171.3561	49.06472	1.50893	1.11797	0.03452	0.12465	7.10992	0.02087	6.235
**'Delta BJ'**	205.8862	57.53077	2.24955	1.27927	0.15171	0.1427	7.32983	0.02815	5.59
**'Delta BJ'**	178.0587	50.46222	1.88114	1.13805	0.16414	0.17037	7.46517	0.0196	4.515
**'Delta BJ'**	177.6427	48.87251	1.43294	1.06997	0.25619	0.12837	7.17697	0.02462	6.235
**'Delta BJ'**	177.9663	47.9321	1.04617	1.02732	0.12297	0.15543	7.41027	0.02581	7.525
**'Delta BJ'**	185.9632	49.95031	1.35299	1.06768	0.22721	0.16069	7.43177	0.02567	6.88
**'Delta BJ'**	213.0973	53.77881	1.5272	1.08003	0.278	0.1481	7.40451	0.02227	3.01

'Delta BJ'	444.5458	77.71003	1.5768	1.08101	0.28267	0.13074	7.2714	0.01313	10.965
'Delta BJ'	339.6613	67.89119	1.34284	1.07987	0.03408	0.11795	7.29033	0.023	11.18
'Delta BJ'	183.467	49.01871	1.24037	1.04221	0.05076	0.1193	7.05866	0.02217	6.665
'Delta BJ'	478.5212	78.95122	1.23081	1.03659	0.11165	0.14814	7.34671	0.01644	10.965
'Delta BJ'	263.2514	58.17406	1.29542	1.02301	0.29257	0.15405	7.41652	0.01505	13.975
'Delta BJ'	240.6936	63.8077	2.51004	1.34608	0.24885	0.16918	7.48277	0.02344	7.31
'Delta BJ'	236.8107	55.02689	1.28705	1.01751	0.05879	0.14125	7.32458	0.01538	7.74
'Delta BJ'	370.4009	70.32908	1.45405	1.06264	0.34087	0.1668	7.53056	0.015	9.245
'Delta BJ'	306.518	63.18657	1.32684	1.03653	0.26834	0.15142	7.51439	0.0169	8.385
'Delta BJ'	214.7614	54.3406	1.53953	1.09417	0.34343	0.14893	7.37546	0.01776	6.235
'Delta BJ'	272.8662	60.14603	1.28358	1.055	0.249	0.15942	7.59424	0.02025	4.515
'Delta BJ'	245.6397	56.22164	1.34794	1.024	0.07005	0.13233	7.21909	0.01465	7.74
'Delta BJ'	280.0773	62.22422	1.55764	1.1001	0.29848	0.14748	7.4605	0.02306	2.795
'Delta BJ'	255.9016	59.47201	1.64565	1.09987	0.09534	0.14163	7.34471	0.01774	1.72
'Delta BJ'	206.5333	51.87971	1.41448	1.03704	0.02749	0.15537	7.342	0.01477	4.73
'Delta BJ'	258.3053	57.44671	1.25087	1.01669	0.12359	0.10506	6.91545	0.01498	4.515
'Delta BJ'	314.8385	62.94469	1.1612	1.00143	0.23045	0.16749	7.5062	0.01104	9.245
'Delta BJ'	465.2546	78.18109	1.33611	1.04545	0.14495	0.15644	7.48941	0.01536	10.75
'Delta BJ'	205.3777	52.91623	1.52515	1.08496	0.0986	0.10897	6.86447	0.01551	6.665
'Delta BJ'	444.8232	76.68964	1.29893	1.05215	0.2628	0.11606	7.12996	0.01866	6.45
'Delta BJ'	232.974	54.67837	1.25835	1.02121	0.24709	0.14792	7.39262	0.01582	8.17
'Delta BJ'	217.7198	53.67905	1.47843	1.05318	0.20703	0.16083	7.42392	0.01423	6.45
'Delta BJ'	189.2914	51.17903	1.60392	1.10114	0.20383	0.16254	7.46297	0.02244	4.3
'Delta BJ'	149.1219	43.68241	1.26716	1.01827	0.08184	0.12698	7.18497	0.01975	4.945
'Delta BJ'	150.3237	43.77142	1.08361	1.01425	0.11021	0.12507	7.08147	0.01841	1.72
'Delta BJ'	174.7767	49.27284	1.71914	1.10541	0.09412	0.13928	7.29495	0.01869	5.375
'Delta BJ'	226.2252	54.15033	1.29644	1.03146	0.28551	0.1708	7.49174	0.0139	7.74
'Delta BJ'	239.1219	56.28743	1.4404	1.05437	0.0376	0.11939	7.28109	0.01598	7.525
'Delta BJ'	248.7367	58.88699	1.35787	1.1094	0.37325	0.14109	7.47382	0.02677	7.955

'Delta BJ'	213.9293	52.83259	1.42119	1.0383	0.05019	0.1301	7.22922	0.01364	5.805
'Delta BJ'	220.1235	54.98797	1.5957	1.0931	0.09467	0.12482	7.2221	0.02077	6.45
'Delta BJ'	249.199	59.4045	1.685	1.12689	0.18195	0.18399	7.62551	0.02124	6.665
'Delta BJ'	236.8107	56.87266	1.66182	1.08692	0.27394	0.15829	7.47058	0.01367	2.795
'Delta BJ'	233.0202	54.09142	1.16248	0.9992	0.1024	0.12888	7.20432	0.01312	9.03
'Delta BJ'	337.2576	65.78785	1.26362	1.02122	0.07717	0.16461	7.51632	0.01365	3.225
'Delta BJ'	312.8046	66.98454	1.68112	1.14147	0.11918	0.15068	7.43384	0.02296	6.88
'Delta BJ'	256.179	57.9124	1.39285	1.04181	0.21419	0.14083	7.27465	0.01598	3.01
'Delta BJ'	325.9787	68.42332	1.80409	1.1429	0.10821	0.14628	7.49839	0.01755	6.88
'Delta BJ'	242.1266	57.79523	1.52533	1.09782	0.01203	0.0942	6.79747	0.03304	2.15
'Delta BJ'	310.1698	63.42285	1.19752	1.03201	0.24639	0.13304	7.47498	0.01396	9.89
'Delta BJ'	152.1727	45.9068	1.72462	1.10207	0.1499	0.15491	7.35481	0.01643	5.375
'Delta BJ'	161.0017	45.46261	1.31656	1.02157	0.07287	0.13305	7.13717	0.01582	5.805
'Delta BJ'	234.0834	55.69188	1.39533	1.05439	0.10083	0.14508	7.28886	0.01593	7.74
'Delta BJ'	388.9834	76.17192	2.04187	1.187	0.3817	0.15659	7.46465	0.01394	9.46
'Delta BJ'	228.444	57.35254	1.40653	1.14582	0.17393	0.13878	7.2641	0.02736	3.01
'Delta BJ'	457.8586	78.77299	1.48147	1.07848	0.37174	0.13744	7.27001	0.01256	9.89
'Delta BJ'	385.9325	74.01848	1.79182	1.12969	0.29634	0.14459	7.36402	0.01498	8.17
'Delta BJ'	142.0494	42.21202	1.23441	0.99821	0.03855	0.16556	7.44366	0.01221	5.59
'Delta BJ'	180.37	49.77788	1.33432	1.0932	0.16732	0.12289	7.1615	0.02814	6.88
'Delta BJ'	156.6103	45.54409	1.19169	1.05398	0.12646	0.144	7.25079	0.02811	7.095
'Delta BJ'	163.0356	45.75931	1.18789	1.02204	0.10743	0.16077	7.33252	0.01892	6.02
'Delta BJ'	439.0913	75.41082	1.34587	1.03063	0.12705	0.14393	7.39819	0.01186	9.89
'Delta BJ'	180.7398	48.36081	1.37753	1.02973	0.16261	0.15339	7.35756	0.01437	6.02
'Delta BJ'	280.4471	60.2688	1.2384	1.03068	0.24359	0.15692	7.46558	0.0143	6.665
'Delta BJ'	532.0498	82.39359	1.19282	1.01537	0.63587	0.16239	7.5297	0.01015	12.685
'Delta BJ'	191.0479	50.60455	1.56164	1.06666	0.3139	0.1377	7.16481	0.01736	6.235
'Delta BJ'	179.4455	50.30785	1.70815	1.12235	0.07794	0.11825	7.07092	0.0324	6.02
'Delta BJ'	241.0634	55.41969	1.18487	1.01388	0.16293	0.13069	7.19699	0.01511	5.805

'Delta BJ'	515.3625	81.83373	1.25803	1.03405	0.34644	0.1434	7.42922	0.01223	12.255
'Delta BJ'	236.5796	56.21992	1.49335	1.06315	0.21025	0.13621	7.25769	0.01634	4.3
'Delta BJ'	245.2236	56.15069	1.23228	1.02315	0.13786	0.15946	7.42524	0.01595	8.17
'Delta BJ'	196.4563	51.25729	1.58006	1.06423	0.0865	0.13324	7.1905	0.01415	1.935
'Delta BJ'	275.2699	60.21548	1.48197	1.04821	0.21454	0.15875	7.44362	0.01195	3.44
'Delta BJ'	181.4794	50.22787	1.68196	1.10625	0.24412	0.15233	7.36051	0.02168	6.235
'Delta BJ'	396.1483	74.42225	1.65764	1.1126	0.26131	0.13457	7.34572	0.0146	5.59
'Delta BJ'	222.2036	54.26213	1.44172	1.05447	0.16853	0.17445	7.50501	0.01717	2.15
'Delta BJ'	285.9941	61.96515	1.50986	1.06839	0.11747	0.15533	7.43998	0.01465	7.955
'Delta BJ'	247.4887	63.87263	2.11788	1.31179	0.11339	0.16077	7.46008	0.05323	7.095
'Delta BJ'	229.0911	56.54371	1.76179	1.11058	0.04127	0.13262	7.16498	0.01452	2.58
'Delta BJ'	233.1589	54.59839	1.30825	1.01741	0.08945	0.13076	7.32699	0.01292	2.58
'Delta BJ'	304.4379	63.29664	1.37937	1.04726	0.24819	0.14001	7.30358	0.01348	4.515
'Delta BJ'	332.5427	69.00425	1.64902	1.13945	0.10605	0.14675	7.50989	0.02428	2.58
'Delta BJ'	265.9324	59.36709	1.42846	1.05465	0.07895	0.12721	7.12914	0.01473	2.795
'Delta BJ'	328.5211	67.16062	1.57515	1.09259	0.12296	0.1548	7.44221	0.01986	3.44
'Delta BJ'	220.031	54.31545	1.47042	1.06697	0.20017	0.14949	7.35478	0.01997	3.655
'Delta BJ'	551.4643	86.15243	1.45479	1.07105	0.19448	0.12922	7.27982	0.0177	4.085
'Delta BJ'	205.8399	52.71886	1.47785	1.07447	0.18936	0.18005	7.57491	0.01981	6.45
'Delta BJ'	208.7521	53.84288	1.70134	1.10514	0.07963	0.16778	7.48824	0.01548	6.235
'Delta BJ'	244.2067	55.79508	1.17217	1.01444	0.11985	0.12444	7.13383	0.01602	6.02
'Delta BJ'	273.6058	61.71274	1.70648	1.10768	0.38149	0.15944	7.42165	0.01596	3.01
'Delta BJ'	197.011	53.4232	1.8292	1.15281	0.05606	0.13968	7.19079	0.02158	5.375
'Delta BJ'	245.917	55.54783	1.15958	0.99847	0.11384	0.13353	7.26501	0.01335	7.095
'Delta BJ'	326.857	64.42045	1.21892	1.01037	0.1412	0.14397	7.40792	0.01091	3.01
'Delta BJ'	225.8554	54.02047	1.33815	1.0282	0.22849	0.104	7.14869	0.01591	7.955
'Delta BJ'	557.3811	84.65883	1.26727	1.02325	0.09835	0.15337	7.43305	0.01083	4.515
'Delta BJ'	314.5149	63.53508	1.27641	1.02135	0.13215	0.12498	7.17521	0.0132	9.46
'Delta BJ'	441.7723	75.2027	1.19391	1.01873	0.15835	0.12493	7.36607	0.01464	3.87

'Delta BJ'	239.8153	55.98342	1.44576	1.04	0.13355	0.13217	7.22732	0.01219	2.795
'Delta BJ'	256.6412	60.85016	1.90447	1.14812	0.13096	0.14585	7.38682	0.01525	6.88
'Delta BJ'	234.823	55.67941	1.39114	1.0506	0.19128	0.14888	7.27447	0.01779	6.88
'Delta BJ'	328.0588	65.29722	1.32524	1.03426	0.41869	0.18443	7.60159	0.01307	9.245
'Delta BJ'	607.3041	88.02831	1.14471	1.01538	0.17122	0.13555	7.33061	0.01062	14.835
'Delta BJ'	256.9186	57.21924	1.26933	1.0141	0.08223	0.146	7.40615	0.01384	9.675
'Delta BJ'	449.2146	76.85132	1.36946	1.04626	0.10857	0.12035	7.19942	0.0125	4.3
'Delta BJ'	229.1373	55.85356	1.54183	1.08342	0.01666	0.13935	7.26432	0.02094	8.17
'Delta BJ'	165.7166	49.32637	1.76474	1.16838	0.22562	0.16478	7.56525	0.03003	6.235
'Delta BJ'	211.6643	54.01338	1.47486	1.09684	0.23666	0.18081	7.53477	0.03049	6.45
'Delta BJ'	298.706	63.10293	1.24661	1.06083	0.16425	0.13345	7.27739	0.02017	3.01
'Delta BJ'	284.1451	61.43496	1.26196	1.05702	0.34987	0.15477	7.42617	0.02707	9.675
'Delta BJ'	428.4595	76.06356	1.55665	1.07457	0.15493	0.12769	7.3846	0.01446	9.675
'Delta BJ'	497.6584	80.31303	1.34914	1.03141	0.19244	0.12512	7.31611	0.01102	12.04
'Delta BJ'	235.8862	55.20448	1.25862	1.0281	0.28883	0.1754	7.56486	0.01562	7.955
'Delta BJ'	228.444	53.73409	1.19567	1.0058	0.16473	0.14529	7.30841	0.01534	3.225
'Delta BJ'	252.6196	57.39511	1.27963	1.0377	0.05734	0.12832	7.3201	0.01744	8.385
'Delta BJ'	197.427	53.17423	1.81193	1.13969	0.05115	0.14478	7.2445	0.02019	5.375
'Delta BJ'	344.6998	67.62653	1.42945	1.0558	0.18154	0.14656	7.41549	0.01336	7.31
'Delta BJ'	332.82	66.06154	1.26281	1.04347	0.12922	0.16394	7.50409	0.01639	9.675
'Delta BJ'	336.3793	66.3017	1.35082	1.03994	0.18118	0.1508	7.32642	0.01462	4.3
'Delta BJ'	259.6458	58.38755	1.42386	1.04484	0.08835	0.11585	7.09146	0.01335	3.44
'Delta BJ'	296.9956	62.46266	1.40554	1.0454	0.1186	0.13423	7.33541	0.01442	8.815
'Delta BJ'	162.8969	46.54514	1.33651	1.05834	0.0668	0.14391	7.23852	0.02759	6.235
'Delta BJ'	250.0773	58.88184	1.27159	1.10326	0.20843	0.1515	7.54145	0.02575	3.225
'Delta BJ'	475.8402	79.28168	1.39673	1.05117	0.09767	0.11948	7.24657	0.01417	4.945
'Delta BJ'	223.3592	53.81945	1.34515	1.03196	0.12593	0.14421	7.37799	0.01709	8.17
'Delta BJ'	549.0606	83.78034	1.24174	1.01731	0.17226	0.11824	7.21755	0.01091	12.685
'Delta BJ'	288.9987	62.70088	1.55405	1.08253	0.50532	0.14714	7.41455	0.01868	7.74

**'Delta BJ'**	499.1838	80.16361	1.31225	1.02443	0.31847	0.14476	7.41046	0.01189	12.47
**'Delta BJ'**	296.0711	65.48019	1.71809	1.15243	0.32917	0.12864	7.11491	0.02778	3.01
**'Delta BJ'**	470.2932	79.88841	1.48725	1.07991	0.44727	0.12613	7.34793	0.01357	11.825
**'Delta BJ'**	236.7645	57.52325	1.70734	1.11214	0.17566	0.14818	7.39499	0.01727	3.87
**'Delta BJ'**	205.3315	52.76702	1.34311	1.0791	0.25212	0.13169	7.18777	0.02094	7.31
**'Delta BJ'**	475.1006	79.28899	1.40481	1.053	0.05207	0.10417	7.14563	0.01476	4.085
**'Delta BJ'**	280.0773	60.66848	1.40928	1.04578	0.24383	0.14867	7.31593	0.0156	6.02
**'Delta BJ'**	208.7521	53.15273	1.15963	1.07699	0.25632	0.1288	7.20449	0.03132	8.815
**'Delta BJ'**	463.8217	77.33292	1.32298	1.02605	0.15805	0.1344	7.365	0.01104	12.04
**'Delta BJ'**	231.3099	56.20014	1.63362	1.0866	0.31017	0.15138	7.47799	0.01535	6.88
**'Delta BJ'**	231.3561	57.48562	1.81172	1.13665	0.09002	0.14296	7.36652	0.02303	6.45
**'Delta BJ'**	316.9648	64.08634	1.3149	1.03112	0.09517	0.15395	7.41281	0.01367	2.365
**'Delta BJ'**	184.3915	48.96883	1.36439	1.03488	0.19153	0.14122	7.21386	0.01845	2.58
**'Delta BJ'**	210.5087	53.41417	1.56413	1.07853	0.23286	0.15021	7.37078	0.02065	5.375
**'Delta BJ'**	283.4517	61.61449	1.24267	1.0658	0.11986	0.14535	7.33062	0.02154	2.58
**'Delta BJ'**	519.8464	85.75963	1.81788	1.12585	0.22651	0.13115	7.33912	0.01117	6.235
**'Delta BJ'**	211.803	52.99793	1.50069	1.0553	0.108	0.14512	7.38091	0.01441	6.665
**'Delta BJ'**	407.1036	73.25738	1.40684	1.04903	0.19258	0.12268	7.21121	0.01521	10.535
**'Delta BJ'**	338.9217	67.80262	1.39046	1.0794	0.14052	0.1594	7.51449	0.01768	3.01
**'Delta BJ'**	281.464	59.92179	1.21858	1.01517	0.41194	0.14186	7.32734	0.01536	7.525
**'Delta BJ'**	226.9648	54.78157	1.42581	1.05221	0.21072	0.13796	7.20127	0.01623	7.74
**'Delta BJ'**	167.427	47.08586	1.4925	1.05377	0.19425	0.1509	7.33874	0.01576	5.59
**'Delta BJ'**	282.8508	60.62398	1.24988	1.034	0.1335	0.14242	7.34553	0.01876	2.58
**'Delta BJ'**	147.088	44.75311	1.61348	1.08358	0.39852	0.13164	7.16665	0.01972	6.665
**'Delta BJ'**	270.2776	58.44603	1.2013	1.00575	0.08697	0.12366	7.23841	0.01433	3.225
**'Delta BJ'**	215.2698	52.52149	1.32857	1.01972	0.10411	0.13365	7.21091	0.01356	5.16
**'Delta BJ'**	315.3932	64.16976	1.3865	1.03896	0.15903	0.15634	7.43335	0.01302	3.225
**'Delta BJ'**	269.3993	59.0562	1.33832	1.03021	0.29928	0.14107	7.42775	0.01271	12.04
**'Delta BJ'**	235.2853	55.69725	1.45716	1.04921	0.17069	0.15878	7.45367	0.01452	7.31

Features extracted from nuclei of different cell lines imaged on the mobile fluorescence microscope

Cell line	'Pro. Area(um^2)'	'Perimeter(um)'	'A.R.'	'Shape Factor'	'Centre Mismatch'	'S.D of Normalized Int'	'Entropy'	'Relative concavity'	'LenghtAt0Corr'
'Mobile HME1'	186.9561	86.34774	1.44606	3.1736	0.49874	0.1843	4.90822	0.04304	4.816
'Mobile HME1'	133.6883	68.87	1.32765	2.8233	0.45003	0.20497	5.57224	0.042	7.31
'Mobile HME1'	176.929	67.29216	1.11671	2.03667	0.1241	0.19664	5.42418	0.02999	8.041
'Mobile HME1'	135.3912	62.61497	1.39309	2.30439	0.36887	0.21068	5.5145	0.03745	6.192
'Mobile HME1'	192.32	80.99127	1.17873	2.7142	0.0274	0.18775	4.64659	0.03899	8.643
'Mobile HME1'	129.0805	64.81773	1.44508	2.5901	0.15681	0.19005	4.87046	0.04681	6.579
'Mobile HME1'	93.64815	52.40376	1.15449	2.33354	0.26787	0.16615	5.57693	0.04689	5.676
'Mobile HME1'	104.9345	68.87254	1.10389	3.5972	0.23227	0.14652	4.0469	0.05535	3.01
'Mobile HME1'	82.47834	58.50511	1.08288	3.30246	0.19396	0.17204	4.42838	0.05063	5.633
'Mobile HME1'	118.1271	65.07018	1.11906	2.85236	0.18082	0.1998	5.11936	0.04582	6.321
'Mobile HME1'	129.0084	80.71607	1.20189	4.01876	0.09729	0.13945	3.74183	0.05711	2.709
'Mobile HME1'	87.11194	60.51364	1.49967	3.34518	0.05244	0.16603	4.38792	0.05352	4.945
'Mobile HME1'	213.3432	91.23559	1.33208	3.10485	0.48272	0.20096	5.60498	0.04488	8.385
'Mobile HME1'	107.4509	84.67749	1.3347	5.31026	0.13608	0.14348	3.65329	0.06948	3.612
'Mobile HME1'	254.1099	98.29624	1.36677	3.02581	0.25185	0.21304	5.57521	0.03528	8.858
'Mobile HME1'	129.6131	69.44896	1.29979	2.96123	0.18624	0.1991	5.02041	0.0483	6.321
'Mobile HME1'	110.6719	58.19796	1.0675	2.43539	0.10512	0.22478	5.3822	0.04501	6.106
'Mobile HME1'	79.58281	54.06171	1.27564	2.92247	0.17799	0.15968	4.40864	0.05029	5.805
'Mobile HME1'	133.1169	62.26473	1.07447	2.31761	0.16881	0.21707	5.35798	0.03599	7.095
'Mobile HME1'	100.667	59.90154	1.22319	2.83648	0.09108	0.19134	4.89058	0.05138	5.891
'Mobile HME1'	110.9677	61.39811	1.14568	2.70336	0.22345	0.17729	4.52497	0.04391	6.923
'Mobile HME1'	92.99176	53.80384	1.24934	2.47726	0.07603	0.16881	4.41806	0.05011	2.322
'Mobile HME1'	103.6439	67.58727	1.29302	3.50733	0.02797	0.18862	4.74195	0.05448	5.418
'Mobile HME1'	159.6094	71.40877	1.23548	2.54235	0.29521	0.2438	5.5244	0.03894	6.966

**'Mobile HME1'**	148.9943	50.97882	1.18431	1.38803	0.09938	0.19344	5.49198	0.01823	7.353
**'Mobile HME1'**	103.3794	46.44899	1.21498	1.66077	0.08699	0.18125	4.91934	0.02659	6.536
**'Mobile HME1'**	159.6649	78.42233	1.42801	3.06521	0.2906	0.18264	5.02255	0.05045	6.794
**'Mobile HME1'**	92.10978	60.90873	1.58113	3.20511	0.05633	0.14721	3.69614	0.05652	4.343
**'Mobile HME1'**	83.08482	46.15603	1.37635	2.04045	0.05549	0.20762	4.91222	0.03891	4.945
**'Mobile HME1'**	92.03582	52.85478	1.38493	2.41547	0.32252	0.16021	3.93597	0.04448	6.837
**'Mobile HME1'**	105.1545	55.8312	1.14689	2.35894	0.09375	0.22912	5.08575	0.04098	6.149
**'Mobile HME1'**	86.99915	47.93902	1.73156	2.1021	0.03829	0.17282	4.38195	0.03191	2.15
**'Mobile HME1'**	87.68328	47.00192	1.18017	2.00496	0.03949	0.23347	5.49508	0.03557	5.332
**'Mobile HME1'**	92.43891	43.60437	1.34145	1.6368	0.09078	0.19376	5.2989	0.02977	5.203
**'Mobile HME1'**	120.1758	51.10219	1.39303	1.72923	0.18763	0.20862	5.63064	0.02975	3.956
**'Mobile HME1'**	105.8996	60.77396	1.18493	2.77543	0.09823	0.15292	4.39219	0.04178	3.311
**'Mobile HME1'**	104.6331	52.38995	1.20824	2.08746	0.21152	0.21057	5.41005	0.03511	7.181
**'Mobile HME1'**	133.189	62.2545	1.31912	2.3156	0.03317	0.21519	5.36154	0.03583	6.192
**'Mobile HME1'**	121.605	53.81304	1.19877	1.89502	0.19317	0.21417	5.73984	0.02825	6.364
**'Mobile HME1'**	129.5964	59.48057	1.29748	2.17244	0.13191	0.19016	5.22646	0.03754	6.235
**'Mobile HME1'**	118.7465	55.94296	1.28668	2.0973	0.13746	0.20034	5.50886	0.03563	6.106
**'Mobile HME1'**	113.6451	57.54398	1.34792	2.31867	0.23634	0.23043	5.48037	0.0415	5.977
**'Mobile HME1'**	182.7163	76.93328	1.12905	2.57775	0.37017	0.21178	4.91377	0.03924	8.213
**'Mobile HME1'**	115.3406	68.57636	1.39099	3.24457	0.19901	0.18968	4.92492	0.05154	5.848
**'Mobile HME1'**	108.1536	56.4845	1.23733	2.34751	0.21182	0.19412	5.0289	0.0425	6.622
**'Mobile HME1'**	177.075	67.48768	1.16665	2.04683	0.21159	0.18076	5.09704	0.02977	9.288
**'Mobile HME1'**	95.60809	51.96279	1.25969	2.2474	0.23168	0.17797	4.6199	0.04617	5.676
**'Mobile HME1'**	113.1976	58.96035	1.25443	2.44384	0.33872	0.22888	5.34136	0.04642	6.708
**'Mobile HME1'**	133.1576	69.96595	1.13081	2.92548	0.18308	0.24841	5.80775	0.05364	6.665
**'Mobile HME1'**	113.4713	52.21494	1.17852	1.91203	0.32624	0.21762	5.38008	0.03265	6.235
**'Mobile HME1'**	103.8861	52.44676	1.22621	2.10703	0.25966	0.19882	5.19336	0.03349	5.633
**'Mobile HME1'**	116.2781	59.81807	1.07902	2.44882	0.14317	0.20242	5.31282	0.04139	6.88
**'Mobile HME1'**	115.459	62.17095	1.2115	2.66402	0.09842	0.20427	5.38192	0.0414	7.052

**'Mobile HME1'**	153.4633	78.31538	1.15325	3.18039	0.15974	0.17061	4.72894	0.04701	7.439
**'Mobile HME1'**	123.8238	57.15173	1.47831	2.09916	0.11614	0.17515	5.12705	0.03646	5.676
**'Mobile HME1'**	123.4041	64.65811	1.29674	2.69592	0.1345	0.21545	5.18546	0.04545	5.934
**'Mobile HME1'**	147.2026	64.23529	1.34605	2.2306	0.38016	0.21435	5.76775	0.03668	6.88
**'Mobile HME1'**	111.0509	51.72173	1.2049	1.91696	0.1415	0.20341	5.45676	0.0322	5.762
**'Mobile HME1'**	110.4981	63.66116	1.37744	2.91867	0.11378	0.1557	4.28132	0.04581	1.935
**'Mobile HME1'**	101.4935	59.02253	1.50488	2.73142	0.07687	0.16981	4.5242	0.05035	5.805
**'Mobile HME1'**	73.97849	41.76822	1.33831	1.87662	0.15312	0.21594	5.22307	0.04057	3.268
**'Mobile HME1'**	106.6947	54.40308	1.12974	2.20747	0.12475	0.19191	4.66449	0.03759	6.106
**'Mobile HME1'**	104.6848	55.66096	1.26277	2.35509	0.104	0.21235	4.95653	0.04028	5.719
**'Mobile HME1'**	98.55355	50.5769	1.22996	2.06549	0.12001	0.23451	5.30891	0.03773	5.848
**'Mobile HME1'**	104.1856	61.86823	1.17603	2.9236	0.34963	0.24469	5.37431	0.04769	5.805
**'Mobile HME1'**	115.9489	63.3728	1.08523	2.75632	0.1661	0.17787	4.70304	0.0443	6.579
**'Mobile HME1'**	149.4454	83.88745	1.32735	3.74715	0.04189	0.19903	5.14236	0.05185	6.493
**'Mobile HME1'**	161.1995	62.17293	1.25838	1.90822	0.10752	0.20263	5.10136	0.03049	8.299
**'Mobile HME1'**	99.41333	42.43648	1.20907	1.44153	0.12332	0.18345	5.22476	0.02316	6.149
**'Mobile HME1'**	100.177	50.41088	1.5593	2.0187	0.11508	0.23018	6.05546	0.03841	4.945
**'Mobile HME1'**	112.8925	51.37103	1.1551	1.86021	0.16129	0.17853	5.09234	0.02745	6.02
**'Mobile HME1'**	116.4038	49.35639	1.09408	1.66537	0.19045	0.16497	5.07396	0.02765	6.665
**'Mobile MCF7'**	261.4523	91.445	1.28122	2.54517	0.15481	0.19865	5.35043	0.02902	9.159
**'Mobile MCF7'**	254.6979	110.9749	1.1667	3.84781	0.43482	0.18922	4.88792	0.04057	9.589
**'Mobile MCF7'**	262.3213	110.018	1.82504	3.67184	0.51398	0.1988	5.55944	0.03914	7.482
**'Mobile MCF7'**	356.1063	136.3382	1.98712	4.1538	0.06747	0.20859	5.48221	0.04062	7.31
**'Mobile MCF7'**	338.9513	117.646	1.1763	3.24943	0.05762	0.1978	5.16498	0.0342	10.965
**'Mobile MCF7'**	339.2878	114.6102	1.50558	3.08084	0.64163	0.18894	5.58609	0.04009	10.75
**'Mobile MCF7'**	247.9047	105.5458	1.60772	3.57592	0.39738	0.16203	4.94388	0.04443	8.084
**'Mobile MCF7'**	195.2063	90.94272	1.65145	3.37157	0.06511	0.20405	5.18761	0.0437	6.923
**'Mobile MCF7'**	209.1792	122.3142	1.40956	5.69148	0.01464	0.17194	4.71323	0.0753	8.213
**'Mobile MCF7'**	354.9008	128.5896	1.89842	3.70762	0.03316	0.20332	5.47229	0.04128	7.697

**'Mobile MCF7'**	179.7394	84.50558	1.18775	3.16168	0.11311	0.22004	5.29947	0.04127	7.783
**'Mobile MCF7'**	221.0942	105.6347	1.37661	4.0163	0.27377	0.19505	5.19146	0.04343	8.256
**'Mobile MCF7'**	214.227	100.8367	1.14188	3.77705	0.57244	0.19512	5.09378	0.04225	8.772
**'Mobile MCF7'**	159.3838	95.04428	1.98444	4.51022	0.22379	0.20215	4.88332	0.09449	4.902
**'Mobile MCF7'**	398.5353	126.8198	1.3074	3.21142	0.13768	0.1911	5.21044	0.03123	9.46
**'Mobile MCF7'**	285.7722	104.0043	1.30802	3.01212	0.11931	0.19538	5.36531	0.03228	9.331
**'Mobile MCF7'**	528.4553	174.8179	1.26586	4.60207	0.07023	0.15669	4.78812	0.04009	5.246
**'Mobile MCF7'**	315.273	128.9405	2.16618	4.19645	0.13198	0.20639	5.19427	0.07164	5.633
**'Mobile MCF7'**	270.0113	110.9304	1.64705	3.62668	0.33552	0.21932	5.55133	0.04029	7.74
**'Mobile MCF7'**	316.2844	124.2671	2.31899	3.8853	0.17555	0.21287	5.33967	0.073	6.407
**'Mobile MCF7'**	363.31	136.0569	1.89534	4.05465	0.09245	0.20845	5.60848	0.04665	7.912
**'Mobile MCF7'**	408.9822	145.1522	1.21833	4.09952	0.54845	0.19251	5.311	0.03824	11.954
**'Mobile MCF7'**	235.631	104.7139	1.23285	3.70311	0.16428	0.19239	5.25759	0.03815	9.159
**'Mobile MCF7'**	252.5863	90.99991	1.46102	2.60893	0.41476	0.18462	5.30028	0.03036	9.116
**'Mobile MCF7'**	253.9435	111.7956	1.84781	3.91654	0.18452	0.20502	5.20649	0.04472	7.654
**'Mobile MCF7'**	310.6486	137.0846	1.34	4.81391	0.40972	0.18485	4.98125	0.04728	10.32
**'Mobile MCF7'**	248.1691	104.8683	1.23876	3.5264	0.16354	0.1997	5.48412	0.04238	9.589
**'Mobile MCF7'**	312.7343	106.6288	1.28827	2.8931	0.23782	0.19439	5.24961	0.03512	9.933
**'Mobile MCF7'**	250.2252	107.2562	1.36936	3.65851	0.02674	0.18709	4.96208	0.03875	8.342
**'Mobile MCF7'**	220.3564	88.06039	1.27602	2.80044	0.19543	0.17623	5.28282	0.03532	8.944
**'Mobile MCF7'**	209.8985	106.3697	1.76119	4.2896	0.14462	0.17413	4.72935	0.04981	7.009
**'Mobile MCF7'**	186.4883	86.24385	1.27947	3.17391	0.18147	0.19804	5.4535	0.03905	7.654
**'Mobile MCF7'**	214.4822	110.0518	2.23991	4.49359	0.19675	0.17855	5.05555	0.08433	5.59
**'Mobile MCF7'**	281.9115	105.1713	1.31425	3.12228	0.22018	0.21996	5.5132	0.03579	9.159
**'Mobile MCF7'**	238.0976	96.79988	1.36338	3.13173	0.0651	0.17181	4.79458	0.03576	8.385
**'Mobile MCF7'**	322.0644	114.4523	1.33957	3.23666	0.663	0.18276	5.06963	0.03756	9.288
**'Mobile MCF7'**	236.3743	124.2458	1.41711	5.197	0.14143	0.17313	4.5711	0.06006	7.697
**'Mobile MCF7'**	205.5607	93.81164	1.19678	3.40693	0.24767	0.19242	5.00334	0.03989	9.546
**'Mobile MCF7'**	324.6733	164.4881	2.2011	6.63151	0.90793	0.1704	4.71423	0.0856	7.396

**'Mobile MCF7'**	320.2024	132.0015	1.92665	4.33035	0.21073	0.17121	4.87674	0.06414	6.02
**'Mobile MCF7'**	361.7328	113.1316	1.08351	2.81559	0.26871	0.17072	5.2444	0.0306	12.986
**'Mobile MCF7'**	385.4333	117.4278	1.23928	2.84697	0.09762	0.16706	5.0616	0.02665	6.45
**'Mobile MCF7'**	319.8899	108.1863	1.15746	2.91161	0.10454	0.18277	5.19456	0.02998	10.922
**'Mobile MCF7'**	229.8769	101.7093	1.22209	3.5811	0.46982	0.21461	5.15232	0.03398	8.385
**'Mobile MCF7'**	211.2593	89.11582	1.33754	2.99146	0.22736	0.19679	5.16697	0.03616	8.342
**'Mobile MCF7'**	422.0324	139.3823	1.24461	3.66319	0.33577	0.16771	4.63632	0.03206	11.18
**'Mobile MCF7'**	347.8986	147.4768	1.23144	4.9749	0.24196	0.14623	4.40179	0.04208	9.933
**'Mobile BJ'**	207.7315	64.65802	1.56664	1.60152	0.06105	0.18803	5.90002	0.01939	7.396
**'Mobile BJ'**	698.2416	151.6447	1.31339	2.62083	0.08052	0.16141	5.49171	0.02252	14.835
**'Mobile BJ'**	278.2394	82.36082	1.50601	1.94005	0.45904	0.14947	5.59139	0.02188	9.675
**'Mobile BJ'**	227.3068	82.33808	1.55335	2.37344	0.28289	0.16206	4.9057	0.03836	7.869
**'Mobile BJ'**	190.1771	74.95928	1.3489	2.35116	0.11114	0.17998	5.42472	0.04943	7.525
**'Mobile BJ'**	368.1581	108.3484	1.31271	2.53747	0.42745	0.16475	5.3686	0.02533	10.492
**'Mobile BJ'**	185.2938	69.88463	1.62872	2.09745	0.24828	0.21659	6.44068	0.08728	6.235
**'Mobile BJ'**	160.8796	62.26512	1.36846	1.91769	0.47621	0.22687	6.39166	0.02871	6.622
**'Mobile BJ'**	319.9769	96.66206	1.31104	2.32372	0.15773	0.13041	4.31387	0.03071	10.019
**'Mobile BJ'**	269.4437	74.16167	1.38228	1.62436	0.23292	0.17966	5.74405	0.02836	9.116
**'Mobile BJ'**	338.0693	86.50581	1.28992	1.76147	0.18207	0.18132	5.7731	0.01845	10.879
**'Mobile BJ'**	312.5975	96.0952	1.53021	2.35076	0.43244	0.20964	5.82466	0.03438	7.568
**'Mobile BJ'**	291.6132	81.74807	1.42233	1.82364	0.66963	0.16056	5.95341	0.02204	9.503
**'Mobile BJ'**	171.1712	63.38359	1.05051	1.86773	0.16642	0.17906	6.1331	0.028	7.912
**'Mobile BJ'**	254.6776	80.59653	1.42987	2.0297	0.17506	0.16898	5.73476	0.02322	8.428
**'Mobile BJ'**	283.6366	77.45745	1.47862	1.68327	0.17192	0.19468	6.09984	0.01803	8.127
**'Mobile BJ'**	181.3499	63.32636	1.27289	1.75971	0.18705	0.18669	6.20114	0.02187	7.267
**'Mobile BJ'**	175.753	72.83129	1.83174	2.40173	0.63286	0.22159	6.17074	0.03521	6.149
**'Mobile BJ'**	196.3287	76.58098	1.69382	2.3771	0.17754	0.24385	6.53399	0.02999	6.407
**'Mobile BJ'**	162.4291	64.07344	1.87053	2.01133	0.12883	0.22364	6.46425	0.02597	5.461
**'Mobile BJ'**	349.5516	113.5283	1.62143	2.93418	0.3519	0.17455	5.12872	0.04723	7.095

**'Mobile BJ'**	339.8665	102.5692	1.31411	2.46329	0.65322	0.13287	4.34738	0.0285	12.814
**'Mobile BJ'**	213.6372	70.67226	1.40202	1.86042	0.31357	0.20001	6.15323	0.03299	7.74
**'Mobile BJ'**	194.3355	79.33341	1.86567	2.57721	0.65146	0.16196	5.58465	0.04027	6.106
**'Mobile BJ'**	190.9851	73.93583	1.56009	2.27772	0.14607	0.18437	5.49425	0.03474	6.837
**'Mobile BJ'**	277.8955	76.83231	1.47587	1.69043	0.65893	0.13931	4.25118	0.01894	2.709
**'Mobile BJ'**	343.3057	87.85979	1.11915	1.78933	0.14568	0.17674	6.07915	0.01929	11.352
**'Mobile BJ'**	249.6686	87.26691	1.76315	2.42731	0.52115	0.16565	5.49606	0.03087	5.461
**'Mobile BJ'**	233.7783	74.11901	1.44267	1.87002	0.23695	0.1868	5.98033	0.02494	7.31
**'Mobile BJ'**	140.3594	59.97816	1.58911	2.03955	0.18631	0.2227	6.30482	0.03578	5.59
**'Mobile BJ'**	163.1484	67.28859	1.87099	2.20846	0.09175	0.18445	6.23687	0.03484	5.848
**'Mobile BJ'**	188.2763	70.3594	1.47929	2.09237	0.12889	0.16684	6.10726	0.02871	8.084
**'Mobile BJ'**	312.7251	96.883	1.516	2.38849	0.40234	0.16223	6.16732	0.02885	9.589
**'Mobile BJ'**	176.6017	66.07101	1.77474	1.96706	0.36154	0.19202	6.19949	0.02815	6.278
**'Mobile BJ'**	226.9703	73.8854	1.96973	1.91398	0.08844	0.21966	6.41625	0.04032	6.192
**'Mobile BJ'**	200.7792	75.3799	1.62686	2.25207	0.22561	0.19239	5.8733	0.03288	6.579
**'Mobile BJ'**	287.6378	102.7453	1.47115	2.92057	0.38514	0.18725	5.1603	0.03615	9.159
**'Mobile BJ'**	224.733	83.00518	1.39421	2.43968	0.39598	0.13908	4.6255	0.04332	7.826
**'Mobile BJ'**	284.2708	79.96078	1.4674	1.78983	0.43208	0.19792	6.23205	0.02303	8.557
**'Mobile BJ'**	217.1077	83.05394	1.43183	2.52834	0.26276	0.15032	5.62388	0.03628	8.385
**'Mobile BJ'**	239.6341	76.53235	1.52509	1.94505	0.65708	0.1495	5.11493	0.02614	8.213
**'Mobile BJ'**	217.3093	71.15343	1.54836	1.85397	0.64143	0.19664	6.21912	0.02626	7.697
**'Mobile BJ'**	218.0285	68.82808	1.41452	1.72905	0.31628	0.18068	5.68081	0.02183	7.181
**'Mobile BJ'**	168.0205	80.14241	1.33033	3.04195	0.34685	0.24482	6.53185	0.05075	6.536
**'Mobile BJ'**	354.6142	136.9132	1.52152	4.20654	0.86386	0.23509	6.23688	0.04561	9.202
**'Mobile BJ'**	319.938	95.04139	1.54954	2.24672	0.34227	0.15415	5.60945	0.03039	8.729

Features extracted from nuclei of BJ cells treated with TNFα and imaged on the mobile fluorescence microscope

'Pro. Area(um^2)'	'Perimeter(um)'	'A.R.'	'Shape Factor'	'Centre Mismatch'	'S.D of Normalized Int'	'Entropy'	'Relative concavity'	'LenghtAt0Corr'
207.7315	64.65802	1.56664	1.60152	0.06105	0.18803	5.90002	0.01939	7.396
698.2416	151.6447	1.31339	2.62083	0.08052	0.16141	5.49171	0.02252	14.835
278.2394	82.36082	1.50601	1.94005	0.45904	0.14947	5.59139	0.02188	9.675
227.3068	82.33808	1.55335	2.37344	0.28289	0.16206	4.9057	0.03836	7.869
190.1771	74.95928	1.3489	2.35116	0.11114	0.17998	5.42472	0.04943	7.525
368.1581	108.3484	1.31271	2.53747	0.42745	0.16475	5.3686	0.02533	10.492
185.2938	69.88463	1.62872	2.09745	0.24828	0.21659	6.44068	0.08728	6.235
160.8796	62.26512	1.36846	1.91769	0.47621	0.22687	6.39166	0.02871	6.622
319.9769	96.66206	1.31104	2.32372	0.15773	0.13041	4.31387	0.03071	10.019
269.4437	74.16167	1.38228	1.62436	0.23292	0.17966	5.74405	0.02836	9.116
338.0693	86.50581	1.28992	1.76147	0.18207	0.18132	5.7731	0.01845	10.879
312.5975	96.0952	1.53021	2.35076	0.43244	0.20964	5.82466	0.03438	7.568
291.6132	81.74807	1.42233	1.82364	0.66963	0.16056	5.95341	0.02204	9.503
171.1712	63.38359	1.05051	1.86773	0.16642	0.17906	6.1331	0.028	7.912
254.6776	80.59653	1.42987	2.0297	0.17506	0.16898	5.73476	0.02322	8.428
283.6366	77.45745	1.47862	1.68327	0.17192	0.19468	6.09984	0.01803	8.127
181.3499	63.32636	1.27289	1.75971	0.18705	0.18669	6.20114	0.02187	7.267
175.753	72.83129	1.83174	2.40173	0.63286	0.22159	6.17074	0.03521	6.149
196.3287	76.58098	1.69382	2.3771	0.17754	0.24385	6.53399	0.02999	6.407
162.4291	64.07344	1.87053	2.01133	0.12883	0.22364	6.46425	0.02597	5.461
349.5516	113.5283	1.62143	2.93418	0.3519	0.17455	5.12872	0.04723	7.095
339.8665	102.5692	1.31411	2.46329	0.65322	0.13287	4.34738	0.0285	12.814
213.6372	70.67226	1.40202	1.86042	0.31357	0.20001	6.15323	0.03299	7.74
194.3355	79.33341	1.86567	2.57721	0.65146	0.16196	5.58465	0.04027	6.106
190.9851	73.93583	1.56009	2.27772	0.14607	0.18437	5.49425	0.03474	6.837
277.8955	76.83231	1.47587	1.69043	0.65893	0.13931	4.25118	0.01894	2.709
343.3057	87.85979	1.11915	1.78933	0.14568	0.17674	6.07915	0.01929	11.352
249.6686	87.26691	1.76315	2.42731	0.52115	0.16565	5.49606	0.03087	5.461
233.7783	74.11901	1.44267	1.87002	0.23695	0.1868	5.98033	0.02494	7.31
140.3594	59.97816	1.58911	2.03955	0.18631	0.2227	6.30482	0.03578	5.59
163.1484	67.28859	1.87099	2.20846	0.09175	0.18445	6.23687	0.03484	5.848
188.2763	70.3594	1.47929	2.09237	0.12889	0.16684	6.10726	0.02871	8.084
312.7251	96.883	1.516	2.38849	0.40234	0.16223	6.16732	0.02885	9.589
176.6017	66.07101	1.77474	1.96706	0.36154	0.19202	6.19949	0.02815	6.278
226.9703	73.8854	1.96973	1.91398	0.08844	0.21966	6.41625	0.04032	6.192
200.7792	75.3799	1.62686	2.25207	0.22561	0.19239	5.8733	0.03288	6.579
287.6378	102.7453	1.47115	2.92057	0.38514	0.18725	5.1603	0.03615	9.159
224.733	83.00518	1.39421	2.43968	0.39598	0.13908	4.6255	0.04332	7.826
284.2708	79.96078	1.4674	1.78983	0.43208	0.19792	6.23205	0.02303	8.557

217.1077	83.05394	1.43183	2.52834	0.26276	0.15032	5.62388	0.03628	8.385
239.6341	76.53235	1.52509	1.94505	0.65708	0.1495	5.11493	0.02614	8.213
217.3093	71.15343	1.54836	1.85397	0.64143	0.19664	6.21912	0.02626	7.697
218.0285	68.82808	1.41452	1.72905	0.31628	0.18068	5.68081	0.02183	7.181
168.0205	80.14241	1.33033	3.04195	0.34685	0.24482	6.53185	0.05075	6.536
354.6142	136.9132	1.52152	4.20654	0.86386	0.23509	6.23688	0.04561	9.202
319.938	95.04139	1.54954	2.24672	0.34227	0.15415	5.60945	0.03039	8.729
389.0962	106.8293	2.00863	2.33407	0.65327	0.16013	4.77154	0.03531	8.17
301.1614	73.1375	1.13223	1.41342	0.21132	0.12493	4.63725	0.01675	10.32
240.2868	85.91692	1.48308	2.44466	0.0914	0.15142	4.99374	0.03253	7.826
236.1487	76.46479	1.27492	1.97028	0.40671	0.15774	4.9918	0.02545	8.6
163.5644	58.35646	1.74783	1.65684	0.48393	0.13237	5.61327	0.02826	6.45
130.1104	56.63659	1.65563	1.96188	0.11609	0.12736	5.13322	0.03567	3.182
270.6585	94.64966	1.41939	2.63394	0.1869	0.13997	3.92826	0.03241	10.621
314.9457	94.19516	1.47843	2.24188	0.43676	0.15542	4.81902	0.02829	11.653
237.2729	74.63858	1.84496	1.8684	0.33896	0.16782	5.3984	0.02713	7.869
199.4331	65.07409	1.48938	1.6897	0.47346	0.17943	5.93151	0.02299	6.751
290.9457	96.03508	1.23562	2.52254	0.53545	0.14612	5.0668	0.03634	9.804
243.7999	85.02902	1.51888	2.35989	0.06481	0.16673	5.02645	0.02901	7.74
132.8414	62.05554	1.47287	2.30684	0.14576	0.13118	5.01444	0.03956	6.364
308.8015	96.36511	1.13148	2.39304	0.39001	0.13239	5.06813	0.02901	12.599
295.9509	90.88755	1.75654	2.22116	0.18315	0.15874	4.87084	0.02737	8.256
230.2375	74.20897	1.83697	1.90339	0.26433	0.1436	5.33339	0.02554	3.096
222.9987	75.1431	1.36149	2.01496	0.29783	0.11739	4.037	0.02883	8.213
179.5823	67.93329	1.58981	2.04499	0.66489	0.16952	5.49862	0.03021	4.128
121.1502	60.53794	1.3165	2.40725	0.1883	0.18325	5.51542	0.06152	5.805
284.7312	94.64876	1.36932	2.50372	0.20766	0.14849	4.40747	0.02998	9.46
210.5826	65.69966	1.43868	1.63115	0.35204	0.17607	5.50924	0.02307	7.181
134.0211	56.98205	1.83202	1.92794	0.32524	0.18804	5.64699	0.02999	5.117
98.9141	59.26299	3.28926	2.82552	0.28	0.18756	5.77115	0.07304	3.354
363.5504	161.8884	1.27373	5.73663	0.28053	0.16139	3.7895	0.06905	4.343
185.1052	79.43123	1.44757	2.7124	0.21137	0.16883	4.67408	0.03779	6.966

Features extracted from nuclei of BJ cells treated with CytoD and imaged on the mobile fluorescence microscope

'Pro. Area(um^2)'	'Perimeter(um)'	'A.R.'	'Shape Factor'	'Centre Mismatch'	'S.D of Normalized Int'	'Entropy'	'Relative concavity'	'LenghtAt0Corr'
207.7315	64.65802	1.56664	1.60152	0.06105	0.18803	5.90002	0.01939	7.396
698.2416	151.6447	1.31339	2.62083	0.08052	0.16141	5.49171	0.02252	14.835
278.2394	82.36082	1.50601	1.94005	0.45904	0.14947	5.59139	0.02188	9.675
227.3068	82.33808	1.55335	2.37344	0.28289	0.16206	4.9057	0.03836	7.869
190.1771	74.95928	1.3489	2.35116	0.11114	0.17998	5.42472	0.04943	7.525
368.1581	108.3484	1.31271	2.53747	0.42745	0.16475	5.3686	0.02533	10.492
185.2938	69.88463	1.62872	2.09745	0.24828	0.21659	6.44068	0.08728	6.235
160.8796	62.26512	1.36846	1.91769	0.47621	0.22687	6.39166	0.02871	6.622
319.9769	96.66206	1.31104	2.32372	0.15773	0.13041	4.31387	0.03071	10.019
269.4437	74.16167	1.38228	1.62436	0.23292	0.17966	5.74405	0.02836	9.116
338.0693	86.50581	1.28992	1.76147	0.18207	0.18132	5.7731	0.01845	10.879
312.5975	96.0952	1.53021	2.35076	0.43244	0.20964	5.82466	0.03438	7.568
291.6132	81.74807	1.42233	1.82364	0.66963	0.16056	5.95341	0.02204	9.503
171.1712	63.38359	1.05051	1.86773	0.16642	0.17906	6.1331	0.028	7.912
254.6776	80.59653	1.42987	2.0297	0.17506	0.16898	5.73476	0.02322	8.428
283.6366	77.45745	1.47862	1.68327	0.17192	0.19468	6.09984	0.01803	8.127
181.3499	63.32636	1.27289	1.75971	0.18705	0.18669	6.20114	0.02187	7.267
175.753	72.83129	1.83174	2.40173	0.63286	0.22159	6.17074	0.03521	6.149
196.3287	76.58098	1.69382	2.3771	0.17754	0.24385	6.53399	0.02999	6.407
162.4291	64.07344	1.87053	2.01133	0.12883	0.22364	6.46425	0.02597	5.461
349.5516	113.5283	1.62143	2.93418	0.3519	0.17455	5.12872	0.04723	7.095
339.8665	102.5692	1.31411	2.46329	0.65322	0.13287	4.34738	0.0285	12.814
213.6372	70.67226	1.40202	1.86042	0.31357	0.20001	6.15323	0.03299	7.74
194.3355	79.33341	1.86567	2.57721	0.65146	0.16196	5.58465	0.04027	6.106
190.9851	73.93583	1.56009	2.27772	0.14607	0.18437	5.49425	0.03474	6.837
277.8955	76.83231	1.47587	1.69043	0.65893	0.13931	4.25118	0.01894	2.709
343.3057	87.85979	1.11915	1.78933	0.14568	0.17674	6.07915	0.01929	11.352
249.6686	87.26691	1.76315	2.42731	0.52115	0.16565	5.49606	0.03087	5.461
233.7783	74.11901	1.44267	1.87002	0.23695	0.1868	5.98033	0.02494	7.31
140.3594	59.97816	1.58911	2.03955	0.18631	0.2227	6.30482	0.03578	5.59
163.1484	67.28859	1.87099	2.20846	0.09175	0.18445	6.23687	0.03484	5.848
188.2763	70.3594	1.47929	2.09237	0.12889	0.16684	6.10726	0.02871	8.084
312.7251	96.883	1.516	2.38849	0.40234	0.16223	6.16732	0.02885	9.589
176.6017	66.07101	1.77474	1.96706	0.36154	0.19202	6.19949	0.02815	6.278
226.9703	73.8854	1.96973	1.91398	0.08844	0.21966	6.41625	0.04032	6.192
200.7792	75.3799	1.62686	2.25207	0.22561	0.19239	5.8733	0.03288	6.579
287.6378	102.7453	1.47115	2.92057	0.38514	0.18725	5.1603	0.03615	9.159
224.733	83.00518	1.39421	2.43968	0.39598	0.13908	4.6255	0.04332	7.826
284.2708	79.96078	1.4674	1.78983	0.43208	0.19792	6.23205	0.02303	8.557

217.1077	83.05394	1.43183	2.52834	0.26276	0.15032	5.62388	0.03628	8.385
239.6341	76.53235	1.52509	1.94505	0.65708	0.1495	5.11493	0.02614	8.213
217.3093	71.15343	1.54836	1.85397	0.64143	0.19664	6.21912	0.02626	7.697
218.0285	68.82808	1.41452	1.72905	0.31628	0.18068	5.68081	0.02183	7.181
168.0205	80.14241	1.33033	3.04195	0.34685	0.24482	6.53185	0.05075	6.536
354.6142	136.9132	1.52152	4.20654	0.86386	0.23509	6.23688	0.04561	9.202
319.938	95.04139	1.54954	2.24672	0.34227	0.15415	5.60945	0.03039	8.729
174.932	63.92234	1.17035	1.85877	0.50568	0.16588	5.15852	0.02795	8.084
110.5332	65.05569	2.15116	3.04697	0.37863	0.20726	6.38514	0.06323	4.214
130.4987	61.48346	1.96166	2.30516	0.07487	0.23203	6.31351	0.04537	4.945
176.1505	62.55025	1.43508	1.76752	0.48119	0.18956	5.48696	0.02832	6.579
269.7358	87.33403	1.29026	2.25019	0.75221	0.14946	4.96774	0.02964	9.331
275.7802	112.6197	1.33252	3.65978	1.04297	0.17252	5.19011	0.04859	10.578
241.7715	83.53249	1.24483	2.29666	0.27825	0.18736	5.788	0.03381	8.987
193.0596	99.00096	2.21751	4.03996	0.27755	0.18992	5.58127	0.05176	6.493
174.7767	71.88744	1.82887	2.35295	0.23239	0.18797	5.08413	0.03864	3.225
223.0412	82.88465	1.3924	2.45106	0.26965	0.19432	5.42195	0.03285	7.869
226.1401	82.02542	2.19163	2.36761	0.05388	0.22765	6.20837	0.03909	6.665
144.0815	74.308	2.35509	3.04967	0.10942	0.18528	5.04953	0.08877	4.816
134.5573	55.53897	1.51019	1.82423	0.18894	0.21599	6.17617	0.04416	5.676
86.94922	56.01262	2.3246	2.87142	0.24752	0.19859	6.05865	0.06153	3.397
162.7379	62.52467	1.62276	1.91163	0.23148	0.23794	6.74713	0.03346	5.762
161.6766	66.36117	1.87665	2.16756	0.24817	0.18855	5.65331	0.03269	6.063
91.34615	47.13239	1.16901	1.93526	0.09953	0.21667	5.79033	0.03807	5.16
254.8458	91.94247	1.9845	2.63964	0.23143	0.20604	6.31851	0.0356	6.665
106.1123	49.05672	1.54342	1.80477	0.06529	0.22837	6.33784	0.03386	4.73
154.1696	64.68017	1.58971	2.1594	0.14828	0.19556	5.94396	0.03712	6.278
290.0489	103.3605	1.49659	2.93108	0.32336	0.14079	4.46331	0.03937	9.159
196.1382	77.45444	1.36065	2.434	0.77577	0.1936	6.09029	0.03429	7.482
373.8789	122.6025	1.5075	3.19932	0.32468	0.11905	3.90693	0.03428	9.718
125.3622	53.63962	1.44785	1.8264	0.37449	0.16481	5.48137	0.03409	5.805
149.4602	65.30358	1.64593	2.27059	0.54603	0.19669	5.93024	0.0419	5.848
162.9172	67.75398	2.15077	2.24229	0.37043	0.17577	5.1887	0.03915	5.547
116.6053	58.902	1.36442	2.36773	0.49345	0.20254	5.43228	0.0491	5.547
125.3862	61.38882	1.51116	2.39177	0.42923	0.19583	5.64868	0.04382	5.289

Features extracted from nuclei of BJ cells under compressive load and imaged on the mobile fluorescence microscope

'Pro. Area(um^2)'	'Perimeter(um)'	'A.R.'	'Shape Factor'	'Centre Mismatch'	'S.D of Normalized Int'	'Entropy'	'Relative concavity'	'LenghtAt0Corr'
207.7315	64.65802	1.56664	1.60152	0.06105	0.18803	5.90002	0.01939	7.396
698.2416	151.6447	1.31339	2.62083	0.08052	0.16141	5.49171	0.02252	14.835
278.2394	82.36082	1.50601	1.94005	0.45904	0.14947	5.59139	0.02188	9.675
227.3068	82.33808	1.55335	2.37344	0.28289	0.16206	4.9057	0.03836	7.869
190.1771	74.95928	1.3489	2.35116	0.11114	0.17998	5.42472	0.04943	7.525
368.1581	108.3484	1.31271	2.53747	0.42745	0.16475	5.3686	0.02533	10.492
185.2938	69.88463	1.62872	2.09745	0.24828	0.21659	6.44068	0.08728	6.235
160.8796	62.26512	1.36846	1.91769	0.47621	0.22687	6.39166	0.02871	6.622
319.9769	96.66206	1.31104	2.32372	0.15773	0.13041	4.31387	0.03071	10.019
269.4437	74.16167	1.38228	1.62436	0.23292	0.17966	5.74405	0.02836	9.116
338.0693	86.50581	1.28992	1.76147	0.18207	0.18132	5.7731	0.01845	10.879
312.5975	96.0952	1.53021	2.35076	0.43244	0.20964	5.82466	0.03438	7.568
291.6132	81.74807	1.42233	1.82364	0.66963	0.16056	5.95341	0.02204	9.503
171.1712	63.38359	1.05051	1.86773	0.16642	0.17906	6.1331	0.028	7.912
254.6776	80.59653	1.42987	2.0297	0.17506	0.16898	5.73476	0.02322	8.428
283.6366	77.45745	1.47862	1.68327	0.17192	0.19468	6.09984	0.01803	8.127
181.3499	63.32636	1.27289	1.75971	0.18705	0.18669	6.20114	0.02187	7.267
175.753	72.83129	1.83174	2.40173	0.63286	0.22159	6.17074	0.03521	6.149
196.3287	76.58098	1.69382	2.3771	0.17754	0.24385	6.53399	0.02999	6.407
162.4291	64.07344	1.87053	2.01133	0.12883	0.22364	6.46425	0.02597	5.461
349.5516	113.5283	1.62143	2.93418	0.3519	0.17455	5.12872	0.04723	7.095
339.8665	102.5692	1.31411	2.46329	0.65322	0.13287	4.34738	0.0285	12.814
213.6372	70.67226	1.40202	1.86042	0.31357	0.20001	6.15323	0.03299	7.74
194.3355	79.33341	1.86567	2.57721	0.65146	0.16196	5.58465	0.04027	6.106
190.9851	73.93583	1.56009	2.27772	0.14607	0.18437	5.49425	0.03474	6.837
277.8955	76.83231	1.47587	1.69043	0.65893	0.13931	4.25118	0.01894	2.709
343.3057	87.85979	1.11915	1.78933	0.14568	0.17674	6.07915	0.01929	11.352
249.6686	87.26691	1.76315	2.42731	0.52115	0.16565	5.49606	0.03087	5.461
233.7783	74.11901	1.44267	1.87002	0.23695	0.1868	5.98033	0.02494	7.31
140.3594	59.97816	1.58911	2.03955	0.18631	0.2227	6.30482	0.03578	5.59
163.1484	67.28859	1.87099	2.20846	0.09175	0.18445	6.23687	0.03484	5.848
188.2763	70.3594	1.47929	2.09237	0.12889	0.16684	6.10726	0.02871	8.084
312.7251	96.883	1.516	2.38849	0.40234	0.16223	6.16732	0.02885	9.589
176.6017	66.07101	1.77474	1.96706	0.36154	0.19202	6.19949	0.02815	6.278
226.9703	73.8854	1.96973	1.91398	0.08844	0.21966	6.41625	0.04032	6.192
200.7792	75.3799	1.62686	2.25207	0.22561	0.19239	5.8733	0.03288	6.579
287.6378	102.7453	1.47115	2.92057	0.38514	0.18725	5.1603	0.03615	9.159
224.733	83.00518	1.39421	2.43968	0.39598	0.13908	4.6255	0.04332	7.826
284.2708	79.96078	1.4674	1.78983	0.43208	0.19792	6.23205	0.02303	8.557

217.1077	83.05394	1.43183	2.52834	0.26276	0.15032	5.62388	0.03628	8.385
239.6341	76.53235	1.52509	1.94505	0.65708	0.1495	5.11493	0.02614	8.213
217.3093	71.15343	1.54836	1.85397	0.64143	0.19664	6.21912	0.02626	7.697
218.0285	68.82808	1.41452	1.72905	0.31628	0.18068	5.68081	0.02183	7.181
168.0205	80.14241	1.33033	3.04195	0.34685	0.24482	6.53185	0.05075	6.536
354.6142	136.9132	1.52152	4.20654	0.86386	0.23509	6.23688	0.04561	9.202
319.938	95.04139	1.54954	2.24672	0.34227	0.15415	5.60945	0.03039	8.729
119.5656	51.99693	1.32394	1.79945	0.2129	0.15239	4.64014	0.0337	6.235
203.9743	70.58484	1.51582	1.94374	0.12691	0.19205	6.32199	0.02512	7.138
194.0932	69.97063	1.84291	2.0073	0.13381	0.1623	6.13701	0.02814	6.235
238.3047	66.84432	1.31091	1.49206	0.08269	0.12434	5.31734	0.02435	9.03
171.8886	75.40351	1.7272	2.63224	0.11155	0.15403	5.31902	0.04091	6.235
225.1472	74.9137	1.78533	1.98356	0.13711	0.12791	4.79013	0.03531	7.31
115.5348	58.27506	1.3762	2.33907	0.19683	0.19234	5.95682	0.03626	5.504
109.5181	47.45669	1.68179	1.63644	0.15944	0.15235	6.11725	0.03266	4.343
95.36772	41.026	1.47648	1.40445	0.1337	0.20408	6.11946	0.02329	5.633
265.5312	80.3679	1.62404	1.93571	0.10185	0.14034	5.28468	0.0241	8.643
150.773	62.09166	1.5103	2.03485	0.33318	0.19698	6.36556	0.03295	5.676
366.8416	88.08993	1.0578	1.68331	0.22457	0.16902	6.19106	0.01858	11.911
262.9796	88.92981	1.60844	2.39311	0.1147	0.14465	4.69916	0.03259	2.193
167.5342	65.1656	1.719	2.01708	0.25727	0.12849	5.02542	0.02964	7.095
248.3189	70.81378	1.21101	1.607	0.5679	0.15845	4.77803	0.01876	10.535
203.4917	68.02763	1.14202	1.80973	0.38804	0.14866	5.1436	0.02629	8.6
131.3123	50.26666	1.59322	1.53125	0.35734	0.17862	5.66864	0.02487	5.719
485.3699	105.2739	1.81041	1.81701	0.60327	0.15665	5.67614	0.02419	5.891
215.1422	80.00352	1.50082	2.36746	0.42319	0.1397	4.35669	0.03613	7.697
130.1493	54.60759	1.48322	1.82328	0.37227	0.19647	5.95001	0.02791	5.977
102.9708	53.06763	1.81048	2.17638	0.0377	0.15822	4.65492	0.0395	4.73
171.4319	60.05926	1.79969	1.6744	0.04818	0.13874	4.8339	0.02815	6.364
309.9978	77.22877	1.34245	1.53105	0.09964	0.12017	4.0852	0.01551	3.526
170.537	69.67982	1.90071	2.26561	0.16252	0.11787	4.61014	0.03772	6.149
210.0353	64.43043	1.70898	1.57282	0.53174	0.13218	5.26593	0.02023	6.751
280.8871	97.172	1.595	2.6751	0.16948	0.08583	4.11439	0.03436	8.643
160.41	68.62465	1.70428	2.33625	0.08276	0.18252	5.20769	0.03587	6.536
147.8682	56.83409	1.85775	1.73833	0.21043	0.18865	6.1011	0.02358	5.633
161.475	60.83657	1.73575	1.82396	0.45546	0.15398	5.90683	0.02593	5.762
168.6362	57.81986	1.48171	1.57759	0.1886	0.15987	5.85794	0.0206	6.493
186.2387	61.70788	1.37406	1.62705	0.18638	0.19649	6.17566	0.0233	7.31
172.4119	57.73029	1.45358	1.53826	0.15945	0.13917	4.81601	0.02373	7.052
164.8032	63.50829	1.26311	1.94754	0.09344	0.16477	6.12975	0.02996	6.88
146.389	55.35713	1.29544	1.66582	0.05851	0.17955	6.12363	0.02848	6.02
106.8186	62.6065	1.42397	2.92	0.0736	0.24559	6.79986	0.05242	5.375
152.7644	58.89207	1.35691	1.80668	0.10946	0.13972	5.08125	0.02823	6.579
172.9111	55.79418	1.52858	1.43267	0.22218	0.10435	4.01633	0.01843	7.009
246.0908	80.77081	1.5628	2.10962	0.25786	0.18076	5.43918	0.02501	7.611
292.1069	110.1818	1.20905	3.30726	0.30558	0.11736	5.16778	0.03745	4.085

116.0562	51.77505	1.073	1.83807	0.03264	0.17952	6.1681	0.03055	6.536
163.5921	57.22414	1.46612	1.59289	0.25803	0.16965	5.56545	0.02123	6.794
153.0417	59.1723	1.48937	1.82061	0.08043	0.15984	5.23504	0.02949	6.364
205.0837	64.44995	1.75833	1.61177	0.19457	0.11685	5.15252	0.02171	6.88
241.5922	71.57488	1.40071	1.68744	0.32263	0.13138	6.00585	0.02106	8.514
185.4103	67.42757	1.8721	1.95133	0.19873	0.08503	4.6513	0.0308	5.762
249.04	69.58273	1.87573	1.54712	0.29389	0.11795	5.40205	0.02589	7.396
202.6412	65.9952	1.56097	1.71036	0.2006	0.12756	4.15992	0.02716	5.332
147.5465	52.67048	1.4739	1.49622	0.14846	0.13043	5.28653	0.02445	6.192

Features extracted from nuclei of MCF7 cells treated with TNFα and imaged on the mobile fluorescence microscope

'Pro. Area(um^2)'	'Perimeter(um)'	'A.R.'	'Shape Factor'	'Centre Mismatch'	'S.D of Normalized Int'	'Entropy'	'Relative concavity'	'LenghtAt0Corr'
330.7436	109.3236	1.03714	2.87559	0.67126	0.16059	5.04664	0.02902	11.567
430.8059	160.1103	1.61165	4.7353	0.98898	0.13209	4.09587	0.03731	10.922
363.1473	139.738	1.5028	4.27894	1.08364	0.16519	5.00151	0.03578	10.105
348.1981	173.6771	1.21479	6.89365	1.03933	0.1387	4.1996	0.06169	12.083
228.627	96.43051	1.1375	3.23662	0.156	0.18187	4.95119	0.04234	8.901
258.8489	109.9625	2.35853	3.71734	0.36421	0.1336	4.73351	0.04351	6.794
204.4162	133.1835	2.22923	6.90519	0.36625	0.14771	3.97436	0.07715	5.719
237.835	124.5796	1.11299	5.19288	0.95328	0.16121	4.03666	0.04654	10.019
148.998	91.8839	1.91375	4.50909	0.07277	0.13782	4.50244	0.06006	5.375
232.8094	127.2568	1.7595	5.53543	0.47631	0.12799	3.57569	0.05323	5.934
196.0661	105.6986	1.38986	4.53447	0.56247	0.15979	4.43466	0.04997	11.524
346.29	142.4825	1.24757	4.66523	0.43427	0.12328	3.7958	0.04061	10.75
227.7617	104.1635	1.99675	3.79088	0.40182	0.14638	4.65325	0.04475	6.794
289.6181	135.0952	1.47599	5.01469	0.17159	0.15656	4.35859	0.04583	8.643
301.4628	107.6518	1.23776	3.05914	0.86402	0.18935	5.26749	0.03552	10.019
255.3377	153.9359	1.62512	7.38508	0.20644	0.1394	3.86691	0.06425	7.697
221.2883	139.7471	1.82576	7.02291	0.07595	0.12429	3.68355	0.06049	2.881
314.4262	150.5402	1.49666	5.73557	0.27911	0.16024	4.34121	0.06224	6.966
351.2102	126.8207	1.3352	3.64421	0.49916	0.14431	4.94077	0.03239	9.933
225.3432	122.3907	1.65623	5.28984	0.45002	0.15564	4.69321	0.05437	7.611
242.9993	133.5163	1.94216	5.83786	0.68241	0.1617	4.30766	0.07169	8.041
315.5855	103.8097	1.38324	2.71737	0.76966	0.18357	5.37422	0.02935	9.804
467.7009	143.9647	1.8271	3.52642	0.11307	0.12366	4.04854	0.03095	6.622
291.9553	123.8097	1.30121	4.17814	0.50577	0.11845	4.09355	0.04363	6.708
437.8303	159.8423	1.3728	4.64374	1.58177	0.12502	4.13223	0.04003	0
325.4517	139.1508	1.48389	4.73451	0.62964	0.14358	4.59287	0.04372	10.062
356.7535	108.7734	1.45437	2.63917	0.72235	0.18615	5.67867	0.02928	10.578
242.1376	95.49823	1.31704	2.99722	0.45232	0.18103	5.7065	0.03849	6.88
195.1841	100.8203	1.83492	4.14421	0.25449	0.19057	5.61493	0.11125	6.235
399.5153	106.6777	1.14789	2.26675	0.62022	0.1932	5.82527	0.0235	12.685
274.2289	132.2974	1.84288	5.07901	0.37352	0.17744	5.33045	0.15656	4.816
357.3451	124.4956	1.22302	3.45152	0.48156	0.19192	5.89618	0.0421	11.782
203.7875	85.63003	1.55417	2.86329	0.11211	0.20001	5.64158	0.0474	7.224
253.4517	96.3323	1.47558	2.91366	0.6461	0.20473	5.48459	0.03766	8.17
156.8229	86.93658	1.79413	3.83518	0.1536	0.19855	5.34431	0.05433	5.762
353.1775	115.9307	2.19697	3.02826	0.61713	0.17309	5.16739	0.039	8.643
275.2662	104.239	1.17996	3.14122	0.17042	0.19379	5.75776	0.03806	9.847
440.9588	121.8037	1.45094	2.6774	0.63494	0.13047	4.97153	0.03141	11.782
281.4289	104.8339	1.28693	3.1076	0.67387	0.18011	4.84729	0.03805	10.879

288.6215	108.677	2.1664	3.25639	0.51346	0.18986	5.40651	0.03518	7.611
357.5448	122.7923	1.81873	3.35584	0.55482	0.20013	5.73364	0.05258	8.127
320.3725	119.656	1.98876	3.55634	0.86652	0.14764	5.41301	0.03747	10.105
416.1637	121.6714	1.44659	2.83076	0.13139	0.14703	4.6493	0.02563	10.449
244.1586	89.94245	1.34808	2.63662	0.16604	0.18697	5.51342	0.04282	8.041
241.7494	85.79988	1.24911	2.42325	0.33182	0.16641	5.4949	0.03107	9.116
335.2256	102.9376	1.24023	2.51537	0.52794	0.17988	5.70296	0.03181	8.987
257.0443	108.7456	2.0856	3.66105	0.53108	0.17859	5.4114	0.04196	7.138
264.1833	121.8413	2.27642	4.47171	0.26852	0.20575	5.91517	0.05099	6.321
212.3558	87.95723	1.43929	2.89914	0.2543	0.18203	5.10822	0.05191	4.515
237.6557	97.02795	2.10208	3.15236	0.83864	0.17256	5.44298	0.06026	5.934
446.6796	141.4945	1.29153	3.56675	1.19688	0.18095	5.29886	0.03038	11.954
377.5196	115.9784	1.84198	2.83534	1.16555	0.21569	5.83603	0.0331	9.675
266.2689	120.4552	1.42887	4.33631	0.43731	0.18272	4.5089	0.04531	8.815
128.4057	65.60832	1.56066	2.66762	0.07853	0.19158	5.66931	0.04278	6.278
309.9904	104.3505	1.33423	2.79531	0.58059	0.18112	5.38189	0.03844	9.589
498.9656	134.8021	1.21874	2.8981	0.15137	0.14536	5.04174	0.02642	8.901
449.3958	156.4138	1.37881	4.33223	0.83974	0.14886	4.63779	0.03856	5.203
329.6397	104.8754	1.49085	2.6552	0.06292	0.18711	5.71109	0.03793	9.374
377.9005	138.3714	1.26528	4.03186	0.48572	0.17612	4.88695	0.05448	10.277
201.2507	80.95434	2.00699	2.59139	0.41005	0.19859	5.76195	0.03793	5.848

Features extracted from nuclei of MCF7 cells treated with CytoD and imaged on the mobile fluorescence microscope

'Pro. Area(um^2)'	'Perimeter(um)'	'A.R.'	'Shape Factor'	'Centre Mismatch'	'S.D of Normalized Int'	'Entropy'	'Relative concavity'	'LenghtAt0Corr'
420.8361	130.9468	1.18748	3.2424	0.364	0.15255	5.06548	0.02854	9.288
389.1442	127.3612	1.39415	3.31707	0.25275	0.17277	5.10214	0.03466	10.406
326.2579	114.7754	1.60734	3.21312	0.10832	0.16745	4.80277	0.0342	10.019
439.0802	155.8495	1.55207	4.40207	0.95098	0.15358	4.79072	0.04248	4.687
363.4875	130.942	1.86595	3.75369	0.42622	0.13668	4.38898	0.05934	6.149
304.8483	100.0781	1.34947	2.61447	0.09224	0.17041	5.27072	0.03008	9.976
355.0357	109.807	1.06436	2.70258	0.22878	0.18228	5.73016	0.02513	11.825
317.5824	135.894	1.94606	4.62737	0.27624	0.18009	5.36545	0.05172	7.826
239.2625	101.2841	2.42819	3.41191	0.09611	0.19934	5.22785	0.04626	6.106
347.7932	111.9204	1.27667	2.86608	0.27612	0.18731	5.46406	0.02995	6.063
298.277	148.7767	1.47735	5.90528	0.04143	0.15523	4.40926	0.0579	9.546
364.6524	144.3783	1.31288	4.54899	0.38024	0.16269	5.26505	0.04094	11.223
336.1242	119.1263	1.40804	3.35974	0.17321	0.14906	4.36094	0.03838	2.709
242.391	96.95795	2.75396	3.08632	0.23366	0.22066	5.85348	0.09564	4.902
236.3041	105.2461	2.30471	3.73019	0.64606	0.22318	5.95443	0.10275	6.063
342.9248	118.2582	1.47948	3.24529	0.79949	0.17585	5.2035	0.03687	9.46
423.0882	123.4005	1.02391	2.86413	0.7435	0.17187	5.47195	0.02971	12.599
220.1142	97.92239	1.86212	3.46662	0.48918	0.16922	5.42988	0.04205	6.923
294.7195	117.0978	1.60466	3.70236	0.4054	0.172	4.96714	0.04049	8.729
323.3938	108.473	1.71119	2.89535	0.95807	0.16973	5.23242	0.03044	8.428
320.0508	115.2741	1.42597	3.30396	0.52748	0.2078	5.76013	0.0342	9.331
259.644	111.5743	2.00012	3.8154	0.77178	0.18888	5.7501	0.05891	7.224
271.0634	97.3892	1.28806	2.78446	0.19492	0.1635	5.35598	0.03262	9.202
272.6351	100.5279	1.11683	2.94973	0.21159	0.21486	5.73964	0.03498	9.718
381.6687	121.9928	1.14667	3.10293	0.48424	0.18211	5.65058	0.02784	12.212
235.6939	92.93908	1.7905	2.91634	0.85067	0.19919	5.46115	0.03446	9.159
199.5681	83.10154	1.20783	2.7537	0.67586	0.22391	5.73571	0.03932	8.686
233.3401	105.0596	2.23724	3.76419	0.30722	0.17262	5.18222	0.07464	5.633
407.1295	154.6477	1.25072	4.6746	0.31878	0.19692	5.47623	0.03899	11.567
208.8926	83.64768	1.63247	2.66548	0.3327	0.22968	6.19429	0.04013	6.837
285.0936	97.21014	1.11513	2.6377	0.297	0.22016	6.16888	0.03042	10.105
360.4736	105.3876	1.37541	2.45186	0.39323	0.18182	5.67693	0.0299	10.277
285.9275	122.6505	1.49816	4.18671	0.15194	0.15309	5.11316	0.04894	9.073
199.2076	90.76298	1.53945	3.2908	0.11023	0.1626	4.84354	0.03926	7.181
437.4734	123.5717	1.51835	2.77764	1.1478	0.22301	6.4366	0.02965	11.137
254.3984	86.77508	1.36179	2.35541	0.67182	0.1943	5.84037	0.03303	8.299
286.7318	96.43214	1.68533	2.58082	0.32555	0.17474	5.28688	0.03153	8.944
289.9066	110.6696	1.53059	3.36193	0.81633	0.22003	6.40921	0.07404	8.256
382.8281	111.9343	1.79081	2.60443	0.81553	0.22181	6.19575	0.0251	9.073

209.5842	93.03613	1.64501	3.28651	0.44847	0.21696	6.06486	0.11173	4.73
356.3541	109.3936	1.59311	2.67234	0.30317	0.1767	5.54319	0.04506	8.944
276.4477	107.9373	1.43036	3.35367	0.78455	0.20145	5.85876	0.03758	9.073
281.3531	91.44728	1.49678	2.36527	0.28698	0.18413	5.60948	0.02921	8.686
367.326	113.317	1.18512	2.78182	0.58128	0.17673	5.65039	0.03222	11.481
243.3044	104.7222	2.1084	3.58689	0.07121	0.22048	6.1397	0.04245	6.45
265.1059	92.5418	1.25261	2.57067	0.79277	0.2126	6.09975	0.03054	8.944
198.2535	97.58605	1.34527	3.82248	0.43997	0.22052	5.65383	0.05053	7.74
218.4057	87.39182	1.69152	2.78271	0.39338	0.2115	5.63103	0.03784	6.708
190.2011	82.03794	1.41323	2.81583	0.20915	0.20268	5.68657	0.05492	7.353
181.5663	98.32032	2.10042	4.23683	0.39675	0.22295	6.07721	0.13312	4.859
222.701	95.72032	2.57413	3.27398	0.62397	0.18234	5.22138	0.09796	5.848
286.4249	92.62927	1.04161	2.38383	0.4477	0.19816	5.62209	0.02687	10.492
230.1136	72.5855	1.07385	1.822	0.15416	0.22252	6.20305	0.02537	9.546
211.6476	78.86608	1.02171	2.33861	0.72632	0.22372	6.2977	0.03236	9.331
236.5389	84.81737	1.25763	2.42023	0.20825	0.16611	4.69668	0.03336	7.869
240.5253	89.01095	1.54013	2.6213	0.72537	0.20018	5.68361	0.03413	7.611
355.3334	102.6871	1.34135	2.36149	0.41626	0.19994	5.9037	0.02603	10.191
219.6409	89.44168	1.43745	2.89839	0.32171	0.18429	5.27291	0.04109	10.191
202.6541	89.14932	1.40672	3.12084	0.18481	0.2158	5.48584	0.04908	7.525
266.1673	99.55394	1.41784	2.96314	0.2594	0.21055	5.76286	0.03925	7.181
387.4524	113.6259	1.61967	2.65171	0.89003	0.20615	6.25029	0.02798	9.46
248.7497	92.80711	1.6325	2.75544	0.38433	0.19992	5.72575	0.04076	8.385
233.0609	82.97228	1.69266	2.35064	0.12991	0.24431	6.48894	0.03131	6.966
176.1875	83.68196	2.17884	3.16285	0.27169	0.21353	6.06654	0.09839	5.418
248.4723	88.96158	1.17322	2.53464	0.53023	0.20753	6.05822	0.02893	9.159
250.5894	90.57073	1.35268	2.60497	0.7126	0.20704	5.85564	0.03016	8.643
264.7287	102.2194	1.36377	3.14091	0.26791	0.17745	5.54105	0.03978	7.998
202.4359	90.93017	1.89287	3.25026	0.07529	0.1821	5.41916	0.05843	6.364
271.9731	94.16785	1.6608	2.59459	0.52864	0.21878	6.15048	0.03233	7.998
305.2422	91.6539	1.09039	2.19002	0.67398	0.23914	6.29821	0.02571	10.406
376.4009	119.9086	1.21602	3.03977	0.49834	0.16378	5.37035	0.03179	11.61
152.6146	79.63643	1.7133	3.30687	0.28531	0.22319	5.63557	0.07881	5.891
247.4184	116.5359	1.87138	4.36795	0.59998	0.20205	5.69704	0.06171	7.525

Features extracted from nuclei of MCF7 cells under compressive load and imaged on the mobile fluorescence microscope

'Pro. Area(um^2)'	'Perimeter(um)'	'A.R.'	'Shape Factor'	'Centre Mismatch'	'S.D of Normalized Int'	'Entropy'	'Relative concavity'	'LenghtAt0Corr'
330.7436	109.3236	1.03714	2.87559	0.67126	0.16059	5.04664	0.02902	11.567
430.8059	160.1103	1.61165	4.7353	0.98898	0.13209	4.09587	0.03731	10.922
363.1473	139.738	1.5028	4.27894	1.08364	0.16519	5.00151	0.03578	10.105
348.1981	173.6771	1.21479	6.89365	1.03933	0.1387	4.1996	0.06169	12.083
228.627	96.43051	1.1375	3.23662	0.156	0.18187	4.95119	0.04234	8.901
258.8489	109.9625	2.35853	3.71734	0.36421	0.1336	4.73351	0.04351	6.794
204.4162	133.1835	2.22923	6.90519	0.36625	0.14771	3.97436	0.07715	5.719
237.835	124.5796	1.11299	5.19288	0.95328	0.16121	4.03666	0.04654	10.019
148.998	91.8839	1.91375	4.50909	0.07277	0.13782	4.50244	0.06006	5.375
232.8094	127.2568	1.7595	5.53543	0.47631	0.12799	3.57569	0.05323	5.934
196.0661	105.6986	1.38986	4.53447	0.56247	0.15979	4.43466	0.04997	11.524
346.29	142.4825	1.24757	4.66523	0.43427	0.12328	3.7958	0.04061	10.75
227.7617	104.1635	1.99675	3.79088	0.40182	0.14638	4.65325	0.04475	6.794
289.6181	135.0952	1.47599	5.01469	0.17159	0.15656	4.35859	0.04583	8.643
301.4628	107.6518	1.23776	3.05914	0.86402	0.18935	5.26749	0.03552	10.019
255.3377	153.9359	1.62512	7.38508	0.20644	0.1394	3.86691	0.06425	7.697
221.2883	139.7471	1.82576	7.02291	0.07595	0.12429	3.68355	0.06049	2.881
314.4262	150.5402	1.49666	5.73557	0.27911	0.16024	4.34121	0.06224	6.966
351.2102	126.8207	1.3352	3.64421	0.49916	0.14431	4.94077	0.03239	9.933
225.3432	122.3907	1.65623	5.28984	0.45002	0.15564	4.69321	0.05437	7.611
242.9993	133.5163	1.94216	5.83786	0.68241	0.1617	4.30766	0.07169	8.041
315.5855	103.8097	1.38324	2.71737	0.76966	0.18357	5.37422	0.02935	9.804
467.7009	143.9647	1.8271	3.52642	0.11307	0.12366	4.04854	0.03095	6.622
291.9553	123.8097	1.30121	4.17814	0.50577	0.11845	4.09355	0.04363	6.708
437.8303	159.8423	1.3728	4.64374	1.58177	0.12502	4.13223	0.04003	0
325.4517	139.1508	1.48389	4.73451	0.62964	0.14358	4.59287	0.04372	10.062
263.2273	103.3993	1.27782	3.23217	0.55749	0.17249	5.3931	0.04078	9.417
415.8845	153.0389	1.59676	4.48148	0.1738	0.19046	5.8036	0.04	10.32
201.0381	114.5428	1.60577	5.19335	0.09203	0.19485	5.82684	0.04842	7.267
198.9653	94.44954	1.37757	3.5679	0.2496	0.20249	5.85095	0.0428	7.095
189.3838	94.4185	1.41892	3.74595	0.07554	0.21411	6.02828	0.04619	7.181
264.2369	109.63	1.68438	3.61956	0.52934	0.20831	5.82045	0.04533	6.923
153.2821	78.43002	1.04545	3.19347	0.43144	0.16094	5.70613	0.04718	7.611
246.0353	107.8858	1.60002	3.76462	0.22037	0.19946	5.86904	0.03828	8.213
236.5703	91.7869	1.56098	2.83394	0.29682	0.18746	5.91773	0.03109	8.858
251.342	97.41327	1.22077	3.00443	0.10379	0.17165	5.53821	0.04278	10.75
280.2067	104.6564	1.25768	3.11059	0.10667	0.18618	5.58848	0.03615	10.191
134.3502	76.77267	1.22163	3.49112	0.2132	0.18172	5.87839	0.06433	6.794
122.5203	67.84807	1.24618	2.9899	0.06092	0.21129	6.08759	0.04352	6.02

253.2686	108.0363	1.23236	3.66731	0.32023	0.18896	5.887	0.03647	9.03
195.9737	91.9223	1.23373	3.43111	0.19928	0.1845	5.70991	0.04159	8.127
150.1758	89.59304	1.62229	4.25342	0.14299	0.17538	5.61417	0.06092	6.02
223.8547	111.6981	2.07772	4.43522	0.05184	0.17771	5.81801	0.06914	5.676
182.8901	77.78859	1.45465	2.63288	0.5659	0.19108	5.70943	0.03852	7.353
281.0369	114.7226	1.51123	3.7267	0.60106	0.1277	4.85572	0.04459	8.901
163.03	89.02011	1.25731	3.86811	0.18265	0.18108	5.89201	0.04272	7.095
160.8149	91.50989	1.28101	4.14381	0.079	0.17015	5.16732	0.04799	7.267
123.197	77.74452	1.11841	3.90418	0.13272	0.2289	5.96077	0.05046	6.622
188.1302	98.34104	1.14741	4.09073	0.16424	0.17951	5.3287	0.03789	8.299
202.3805	105.3577	1.83134	4.36469	0.23441	0.17789	5.63756	0.049	5.676
293.795	114.843	1.23817	3.57236	0.40847	0.17908	5.68705	0.03157	9.976
294.3793	113.8199	1.41153	3.50203	0.42851	0.19421	5.80718	0.03346	9.116
245.4622	115.1728	1.40634	4.30037	0.32086	0.13519	4.80485	0.04535	9.159
221.6526	84.92556	1.11049	2.58937	0.24485	0.16858	5.71713	0.03558	9.46
197.5065	88.15912	1.55076	3.13143	0.46872	0.2049	5.95357	0.03535	7.138
